# Clinical potential of the gut microbiome in oncology: a scoping review of treatment response, toxicity and biomarker development

**DOI:** 10.1007/s00520-026-10918-1

**Published:** 2026-06-20

**Authors:** João Daniel de Souza Menezes, Matheus Querino da Silva, Emerson Roberto dos Santos, Stela Regina Pedroso Vilela Torres de Carvalho, Mikaell Alexandre Gouvea Faria, Rita de Cássia Helú Mendonça Ribeiro, Júlio César André

**Affiliations:** FAMERP (Faculdade de Medicina de São José Do Rio Preto), São José Do Rio Preto, Brazil

**Keywords:** Microbiome, Tumor biomarkers, Precision medicine, Cancer, Scoping review

## Abstract

**Purpose:**

This scoping review aimed to systematically map and critically describe the current evidence on the role of the gut microbiome as a biomarker in oncology, including microbiome-based predictive models and microbial signatures associated with treatment response, toxicity and disease course, and to identify methodological gaps and challenges for clinical translation in precision medicine.

**Methods:**

This scoping review was conducted following the Joanna Briggs Institute (JBI) guidelines for scoping reviews and reported according to the PRISMA Extension for Scoping Reviews (PRISMA-ScR). The search was performed in five electronic databases (PubMed/MEDLINE, Web of Science, Scopus, SciELO, and LILACS) using a structured PICO strategy. Studies involving adult cancer patients undergoing systemic oncological therapies (including chemotherapy, immunotherapy and combined regimens), with gut microbiome analysis and the investigation, development or validation of microbiome-based biomarkers or predictive models, were included.

**Results:**

The literature demonstrates that specific microbial taxa significantly influence the efficacy of immunotherapies (e.g., AUCs up to 0.88 for ICI response prediction) and chemotherapies, and modulate toxicity (e.g., mucositis reduction from 47.1% to 25% with probiotics). Microbiome-based predictive models often outperform clinical markers (e.g., AUC of 0.88 vs. 0.50 in urothelial carcinoma), and variations in microbiota composition can predict disease progression. The literature mapping of the 20 included studies demonstrates that specific microbial taxa significantly influence the efficacy of immunotherapies (e.g., AUCs up to 0.88 for ICI response prediction) and chemotherapies. Regarding toxicity, while the review focuses on baseline biomarkers, exploratory intervention-based data were addressed, showing that a probiotic cocktail reduced Grade 3–4 oral mucositis from 47.1% to 25%. Furthermore, microbiome-based predictive models demonstrated enhanced discriminatory accuracy in predicting patient outcomes compared to standard clinical classification markers alone (e.g., achieving an Area Under the Curve [AUC] of 0.88 vs. 0.50 for clinical factors in urothelial carcinoma), though these models remain in early exploratory stages.

**Conclusion:**

Overall, the evidence suggests a growing interest and potential for microbiome-based predictive models in oncology; however, their clinical translation remains limited by methodological heterogeneity, insufficient external validation, and incomplete mechanistic understanding.

**Supplementary Information:**

The online version contains supplementary material available at 10.1007/s00520-026-10918-1.

## Introduction

Cancer, a significant global health problem, remains one of the leading causes of morbidity and mortality worldwide [1–3]. Despite notable therapeutic advances, including the development of targeted chemotherapies, innovative immunotherapies, and targeted therapies, the inherent heterogeneity in patient response, the intrinsic toxicity of treatments, and the emergence of resistance mechanisms continue to pose persistent challenges [4, 5]. Such obstacles underscore the urgent need for innovative biomarkers and precision medicine strategies that enable more effective patient selection and optimization of therapeutic regimens [6].

In this scenario, the gut microbiome, a complex ecosystem composed of trillions of microorganisms inhabiting the human gastrointestinal tract, has emerged as a critical factor in modulating host physiology, metabolism, and the immune system. Its multifaceted influence extends to health and disease, with increasing recognition of its role in chronic pathologies. More recently, the literature has highlighted the complex interaction between the microbiome and cancer, not only in carcinogenesis and tumor progression but, crucially, in modulating the response to various oncological treatment modalities [6, 7], comprehensively encompassing both therapeutic efficacy and treatment-induced toxicity profiles across chemotherapy and immunotherapy regimens [6–8].

Understanding how the gut microbiome interacts with anti-cancer therapies has evolved rapidly. Initially focused on identifying associations between microbial profiles and clinical outcomes, current research is advancing towards the development of sophisticated predictive models. These models aim to unravel the mechanisms by which the microbiome can directly influence the efficacy of chemotherapeutics and immunotherapeutics, mitigate adverse events, and even predict the disease trajectory. The literature demonstrates that this modulation occurs through immunological, metabolic interactions, and even drug biotransformation, offering a unique understanding of host-treatment interactions that traditional biomarkers may not capture [9–12].

The need for deeper investigation into the predictive role of the microbiome is justified by the incessant search for more personalized, effective, and less morbid therapeutic approaches for oncological patients. The literature points to the microbiome as an accessible and non-invasive source of biomarkers, with the potential to integrate information about the host's internal environment, its immunological and metabolic response to treatment. In this context of rapid scientific evolution and the growing understanding of the microbiome's relevance in oncology, a comprehensive and systematic synthesis of existing knowledge becomes fundamental.

It is for this reason that this review adopts a scoping review methodology, an ideal format for mapping the extent and type of available literature in a rapidly evolving research front. This approach allows for the identification and categorization of studies that address the clinical potential of gut microbiome-based biomarkers in oncology, including both formal predictive models and microbial signatures associated with treatment response, toxicity and disease trajectory. The main objective of this review is, therefore, to systematically mapcomprehensively map and characterize the existing literature on the role of the gut microbiome as a biomarker in oncological treatment response practice, with three specific aims: (i) to identify and summarize microbiome-based predictive models and microbial signatures associated with treatment response, toxicity and prognosis in adult cancer patients; (ii) to describe the methodological approaches used for microbiome profiling and model development, including performance metrics and validation strategies; and (iii) to highlight methodological limitations, sources of bias, and key challenges for the clinical translation of gut microbiome biomarkers and predictive models in precision oncology. Given the heterogeneity of cancer types, therapeutic modalities, microbiome assessment techniques, and analytical approaches, a scoping review was deemed the most appropriate methodology to map the existing evidence, clarify key concepts, and identify knowledge gaps, rather than to quantitatively synthesize outcomes or assess intervention effectiveness.

## Materials and methods

### Study design

This scoping review was conducted following the Joanna Briggs Institute (JBI) guidelines for scoping reviews and reported according to the PRISMA Extension for Scoping Reviews (PRISMA-ScR) [13, 14]. Consistent with the objectives of a scoping review, this study did not aim to formally compare predictive performance between models, assess causality, or evaluate the methodological quality or risk of bias of individual studies. Instead, the focus was on mapping the breadth, characteristics, and reported clinical potential of microbiome-based predictive models, as well as identifying methodological patterns, knowledge gaps, and translational challenges [13].

### Guiding question

The guiding question defined for this review was: “What is the clinical potential of gut microbiome–based biomarkers and predictive models in oncological treatment response practice?” To adequately structure the search strategy, the PICO framework was used, where the population (P) comprises adult patients with a cancer diagnosis undergoing systemic or loco-regional oncological therapies (including chemotherapy, immunotherapy and combined regimens), the intervention/exposure (I) refers to gut microbiome analysis for the identification, development or validation of biomarkers and predictive models, there is no specific comparison (C) as the review predominantly includes observational studies and secondary analyses, and the outcome (O) encompasses oncological treatment response, therapeutic effectiveness, toxicity, prognosis and clinical applicability, as shown in Table 1 [13].

**Table 1:** PICO structure for the guiding question

Component	Description
P (population)	Adult patients (≥ 18 years) with a cancer diagnosis undergoing systemic or loco-regional oncological therapies (including chemotherapy, immunotherapy and combined regimens), the intervention/exposure
I (intervention/exposure)	Gut microbiome analysis for the development of predictive models
C (comparison)	Not applicable (observational studies)
O (outcome)	Oncological treatment response, therapeutic effectiveness, clinical applicability

In line with the exploratory nature of a scoping review, we did not restrict inclusion only to studies reporting fully specified predictive models with formal metrics (e.g., AUC). We also considered studies that identified microbiome signatures associated with relevant clinical outcomes (response, toxicity, progression), provided they included a clearly defined adult cancer population, gut microbiome profiling, and an explicit link to oncological treatment.

### Search strategy

The search was performed in five electronic databases: PubMed/MEDLINE, Web of Science, Scopus, SciELO, and LILACS. The search strategy was developed using controlled descriptors (MeSH terms) and keywords related to the gut microbiome, chemotherapy treatment, and predictive models. For each database, the strategy was adapted considering its indexing specificities and syntax, as shown in Table 2.

**Table 2:** Search strategy

Database	Search strategy
PubMed/MEDLINE	(((“Gastrointestinal Microbiome”[Mesh] OR “Gut Microbiota”[tiab] OR “Intestinal Microbiome”[tiab] OR “Microbiota”[tiab]) AND (“Antineoplastic Agents”[Mesh] OR “Chemotherapy”[tiab] OR “Cancer Treatment”[tiab] OR “Oncology”[tiab]) AND (“Predictive Models”[tiab] OR “Biomarkers”[tiab] OR “Treatment Response”[tiab] OR “Therapeutic Efficacy”[tiab])))
Web of Science	TS = ((“gut microbiome” OR “intestinal microbiota” OR “gastrointestinal microbiome”) AND (“chemotherapy” OR “cancer treatment” OR “antineoplastic”) AND (“predictive model*” OR “biomarker*” OR “treatment response” OR “clinical application”))
Scopus	TITLE-ABS-KEY((“gut microbiome” OR “intestinal microbiota”) AND (“chemotherapy” OR “cancer treatment”) AND (“predictive” OR “biomarker” OR “response” OR “efficacy”))
SciELO	(“microbioma intestinal” OR “microbiota intestinal”) AND (“quimioterapia” OR “tratamento oncológico”) AND (“preditivo” OR “biomarcador” OR “resposta”)
LILACS	(“microbioma intestinal” OR “microbiota gastrointestinal”) AND (“antineoplásicos” OR “quimioterapia”) AND (“modelos preditivos” OR “biomarcadores”)

### Eligibility criteria

Inclusion and exclusion criteria were established to ensure that only studies relevant to the guiding question were included in the review. We included studies involving adult cancer patients undergoing systemic or loco-regional oncological therapies (including chemotherapy, immunotherapy, targeted therapies, chemoradiotherapy and combined regimens), in which the gut microbiome was profiled and explicitly linked to treatment response, toxicity, prognosis or diagnostic/staging performance, through either biomarker identification or the development/validation of predictive models. The inclusion criteria are defined in Table 3.

**Table 3:** Inclusion and exclusion criteria

Inclusion criteria	Exclusion criteria
Studies involving adult patients (≥ 18 years) with cancer	Studies in pediatric populations (< 18 years)
Gut/intestinal microbiome analysis	Studies focused only on oral, cutaneous, or other site microbiomes
Chemotherapy treatment (monotherapy or combined)	Studies exclusively with radiotherapy or surgery
Development or validation of predictive models	Purely descriptive studies without shedding light on the predictive component
Assessment of therapeutic response/effectiveness	Studies focused only on toxicity or adverse effects
Observational studies, clinical trials, cohort studies, and literature reviews	Case reports, letters to the editor, editorials
No language exclusion	-
2015–2025	Publications prior to 2015
Full text available	Abstracts only or incomplete texts
Studies with discussed clinical applicability	Purely in vitro experimental studies

Although chemotherapy was initially defined as the primary therapeutic context of interest, the scope was expanded a priori to include studies evaluating immunotherapy, targeted therapies and combined treatment modalities (e.g., chemoimmunotherapy or chemoradiotherapy), provided that gut microbiome biomarkers or predictive models were explicitly linked to treatment response, toxicity, prognosis or diagnostic performance. This decision reflects the integrative role of the microbiome across oncological therapeutic strategies and aligns with the exploratory nature of a scoping review.

To ensure a comprehensive overview of the literature landscape, review and meta-analysis articles were tracked in our supplementary material to cross-reference and prevent missing primary sources. However, to maintain data integrity and prevent redundancy or the artificial duplication of patient cohorts within our qualitative synthesis, these secondary review articles were strictly segregated and excluded from the direct mapping of primary study results.

### Article selection process

The selection process followed a systematic and reproducible flow. Initially, all references identified in the databases were imported into EndNote software for automatic duplicate removal, followed by manual verification. Screening by title and abstract was performed by two independent reviewers using the Rayyan platform, which allows for blinded assessment. Agreement between reviewers was evaluated by Cohen's Kappa coefficient, and disagreements were resolved by consensus or by a third reviewer. Articles selected in the first screening were subjected to full-text reading by two independent reviewers, using a standardized eligibility form. All reasons for exclusion were recorded. Additionally, a manual search was performed in the references of the included articles and a citation search of the selected studies to identify additional relevant studies [13, 14].

### Data extraction

Data extraction was performed by two independent reviewers using a standardized instrument developed specifically for this review. The instrument was pre-tested on five articles for calibration and necessary adjustments. Disagreements in extraction were resolved by consensus or by a third reviewer. The instrument used for data extraction is presented in Table 4.

**Table 4:** Data extraction instrument

Category	Extracted variables	Details
A. Study identification	A1. Main author (year)	First author and year of publication
	A2. Title	Full article title
	A3. Journal	Journal name and impact factor (if applicable)
	A4. Country/region	Location(s) where the study was conducted
	A5. Funding	Declared funding source and type (public, private, industrial)
	A6. Conflicts of interest	Authors’ declaration of conflicts (yes/no, which ones)
B. Methodological design	B1. Study type	Prospective/retrospective/cross-sectional/cohort/clinical trial/meta-analysis/review
	B2. Follow-up duration	Patient follow-up time (if longitudinal)
	B3. Study center(s)	Single-center/multicenter
	B4. Recruitment period	Start and end dates of data collection/recruitment
	B5. Ethical approval	Ethics committee and approval number, registration on platform (e.g., ClinicalTrials.gov)
C. Study population	C1. Sample size	Total number of participants (patients, controls)
	C2. Age	Mean/median and standard deviation/range (with age groups)
	C3. Sex	Male/female distribution (%)
	C4. Cancer type	Primary location, histology (e.g., colorectal, breast, lung, melanoma, gastric, esophageal, urothelial)
	C5. Tumor stage	Classification (e.g., I, II, III, IV, locally advanced, metastatic)
	C6. Performance status	ECOG/Karnofsky (mean/range/distribution)
	C7. Relevant comorbidities	Pre-existing medical conditions, concomitant medications (e.g., PPIs, antibiotics)
	C8. Previous treatments	Surgery, radiotherapy, previous chemotherapy, targeted therapies
D. Chemotherapy protocol	D1. Chemotherapy regimen	Drugs used, dosages, frequency (e.g., FOLFOX, TP, MVAC)
	D2. Line of treatment	First/Second/Subsequent line, neoadjuvant/adjuvant
	D3. Treatment intent	Curative/palliative/neoadjuvant/adjuvant
	D4. Number of cycles	Planned vs. completed cycles, or treatment duration
	D5. Combined therapies	Immunotherapy, targeted therapy, concomitant radiotherapy
	D6. Dose modifications	Rate of reduction, delays, suspensions due to toxicity
E. Microbiome analysis	E1. Sample type	Feces, rectal swabs, biopsies (which site), blood
	E2. Timing of collection	Pre-treatment (baseline), during treatment, post-treatment, multiple time points
	E3. Sequencing technique	16S rRNA (regions V1-V2, V3-V4, V4, V5-V6), shotgun metagenomics, qPCR
	E4. Sequencing platform	Illumina (MiSeq, HiSeq, NovaSeq), ion torrent, others
	E5. Sequencing depth	Average number of reads per sample
	E6. Bioinformatic pipeline	Software and tools used (e.g., QIIME, DADA2, Bowtie2, Kraken, MetaPhlAn)
	E7. Reference database	Greengenes, SILVA, RDP, GTDB, NCBI NR, others
	E8. Quality control	Host DNA removal, read filtering, OTU/ASV clustering
F. Predictive model	F1. Model type	Machine learning classifier, regression, risk score, microbial signature, network model
	F2. Algorithm(s) used	Random forest, SVM, logistic/linear regression, LASSO, deep learning, LEfSe, CoxPH
	F3. Predictor variables	Microbial taxa (genera, species, strains), abundance, diversity (alpha, beta), metabolites, microbial genes, clinical factors
	F4. Feature selection method	Univariate analysis, LASSO, recursive feature elimination, SHAP values
	F5. Model validation	Internal validation (cross-validation, hold-out), external validation (independent cohorts)
	F6. Performance metrics	AUC, sensitivity, specificity, accuracy, PPV, NPV, F1-score, Brier score
	F7. Training set size	Number of samples used to develop the model
	F8. Test set size	Number of samples used to test the model
	F9. Model interpretability	Most important features identified, taxa weight, visualizations (e.g., SHAP, cladograms, heatmaps)
	F10. Model availability	Code, software, web platform available for use
G. Evaluated outcomes	G1. Primary study outcome	Objective response rate (ORR), pathological complete response (pCR), progression-free survival (PFS/SLP), overall survival (OS/SG), disease control rate (DCR)
	G2. Response criteria	RECIST (specific version), TRG (specific version), pathological criteria
	G3. Evaluation time	Response evaluation intervals (e.g., 6 weeks, 12 months)
	G4. Secondary outcomes	Toxicity (irAEs, mucositis, diarrhea, neutropenia), quality of life (QoL), microbiome alteration, FMT implantation
	G5. Associated clinical/laboratory biomarkers	NLR, albumin, LDH, CEA, PD-L1 status, TMB, MSI
	G6. Associated lifestyle/medication factors	Smoking, diet, use of PPIs/antibiotics
	G7. Associated host genetic factors	RAS, BRAF mutation, MMR status, HLA
	G8. Intervention results (if applicable)	Effects of probiotics/FMT on response or toxicity
H. Main results and key findings	H1. Key microbial taxa/species	Specific microorganisms identified as significantly associated with response/resistance/toxicity (e.g., *Fusobacterium*, *Bacteroides*, *Akkermansia*, *Roseburia*, *Lactobacillus*). Include metrics like HR, OR, LDA score
	H2. Direction of association	Clearly indicated if enriched in responders/non-responders, or associated with higher/lower toxicity/PFS/OS
	H3. Predictive model performance	Numerical values of performance metrics (AUC, Sens, Spec, Acc) for the primary outcome, with 95% CI when available
	H4. Microbial diversity findings	Significant differences in alpha/beta diversity between response/toxicity groups
	H5. Microbial functional/metabolic findings	Metabolic pathways or metabolites (e.g., SCFAs, nucleotides, bile acids, tryptophan) identified as mediators
	H6. Preclinical/in vitro model findings	Validation results in animal or cellular models that corroborate or elucidate mechanisms
	H7. Insights into findings: unique contribution	What is the main contribution of this article to the guiding question? What new insight does it offer on the clinical potential of the microbiome in oncological treatment response? What is the “big discovery” or “turning point” of the study? (Concise and impactful synthesis)
	H8. Validation status (internal/external)	Was the model validated internally? Externally? With what results?
I. Article discussion/implications	I1. Authors’ interpretation	Contextualization of findings, comparison with existing literature, justifications for consistencies/inconsistencies
	I2. Proposed biological mechanisms	How the microbiome influences host physiology, drug metabolism, immune response (molecular pathways, cellular interactions). Include results from functional validations
	I3. Correlations with other biomarkers	How the microbiome interacts with PD-L1, TMB, NLR, inflammation markers
	I4. Impact of interventions (if applicable)	How interventions (diet, probiotics, FMT) acted on proposed mechanisms and treatment response
	I5. Consistency/inconsistency of findings	Harmony or divergence with other studies, reasons for variability
	I6. Transferability/generalization	Discussion on the applicability of findings to other populations, cancer types, therapeutic regimens, geographical regions
	I7. Causality vs. association	Level of evidence for causality discussed
	I8. Implications for treatment	Potential implications of findings for stratification and therapeutic management, precision medicine, development of new therapies
J. Potential for clinical application and barriers	J1. Potential for implementation in practice	Feasibility of using models/biomarkers in routine clinical practice (e.g., screening, prognosis, monitoring)
	J2. Advantages	Non-invasive, cost-effective, complementary to other biomarkers
	J3. Barriers to implementation	Microbiota heterogeneity, lack of standardization (collection, analysis), regulatory challenges, cost, need for robust external validation
	J4. Cost-effectiveness	Economic analysis or discussion on financial impact
	J5. Recommendations for future research	Need for randomized clinical trials, longitudinal studies, external validation, high-resolution metagenomics, functional studies
	J6. Potential for personalization	Contribution to precision medicine, patient stratification
K. Limitations and bias of the original study	K1. Limitations declared by authors	Sample size, non-randomized design, specific population, lack of long-term data, uncontrolled confounding factors (diet, medications)
	K2. Selection bias	Restrictions in inclusion/exclusion criteria, recruitment type
	K3. Confounding bias	Uncontrolled variables (lifestyle, diet, use of OTC medications), ethnicity
	K4. Generalization	Applicability of results to other populations, cancer types, or treatment regimens
	K5. Reproducibility	Availability of data/protocols for replication

### Analysis and data synthesis

The extracted data were analyzed through narrative synthesis, considering the expected heterogeneity among studies. A descriptive analysis of the characteristics of the included studies was performed, presenting frequencies and proportions of the main variables. The findings were mapped by cancer type, chemotherapy protocol, and characteristics of the developed predictive models (Supplemental Material S1–S21). Special attention was given to discussing clinical applicability, identifying gaps in current knowledge, and formulating recommendations for future research [13, 14].

## Results

This scoping review is based on a robust set of 20 scientific articles exploring the intricate relationship between the gut microbiome and cancer, as detailed in Table 5. These studies were published over a decade, extending from 2015 to 2025, which attests to the dynamic and growing relevance of the research field. The dating of the articles, in fact, demonstrates the evolution of knowledge, with significant publications such as Feng et al. (2015) [15] already mapping microbiome development in the adenoma-carcinoma sequence in colorectal cancer in 2015, up to more recent articles such as Kim et al. (2025) [16] and Gazzaniga and Kasper (2025) [17] that deepen understanding of specific mutations and in comprehensive reviews. The effervescence of research is particularly visible between 2021 and 2024, a period that concentrates most of the publications included in this sample (check individual details of each article in supplemental material Tables S1–S20, with data extraction for each of the 20 articles).

**Table 5:** Summary of articles included in the review

Title	Lead author (year)	Country	Study design	Objective	Main results (statistical data)	Conclusion
The gut microbiome and cancer response to immune checkpoint inhibitors	Gazzaniga FS and Kasper (2025)	USA	Review	To explore the relationship between the gut microbiome and response to immune checkpoint inhibitors (ICIs), investigating bacteria associated with response, mechanisms of action, and clinical translation of microbiome-based therapies	Presents several bacterial species (e.g., *Bifidobacterium*, *Akkermansia muciniphila*, *Bacteroides fragilis*) that promote ICI response in preclinical models. In patients, responder microbiomes differ from non-responders, and Fecal Microbiota Transplants (FMTs) from responders transfer the response. Antibiotics can abrogate the antitumor effects of ICIs	The gut microbiome and immunotherapy is a rapidly expanding field. Understanding specific bacteria and their mechanisms is crucial for developing safe and effective immunotherapies, moving beyond FMTs, which are a temporary solution
The role of gut microbiota and metabolites in cancer chemotherapy	Li S et al. (2024)	China (Hong Kong)	Review	To elaborate on the role of the gut microbiome and its metabolites in the efficacy and adverse effects of chemotherapy, and to discuss the clinical potential of modulating the microbiome for cancer treatment	*Fusobacterium nucleatum* promotes chemoresistance (TLR4/MYD88-dependent; BIRC3 upregulation). *Akkermansia muciniphila* enhances doxorubicin (DOX) efficacy. *Lactobacillus* and *Bifidobacterium* reduce gastrointestinal (GI) toxicity and neuropathy. Butyrate increases 5-fluorouracil (5-FU) efficacy via GPR109a-AKT. Urolithin A increases cytotoxicity via drug transporter downregulation. Secondary bile acids like ursodeoxycholic acid (UDCA) can remodel the microbiome	The gut microbiome is a crucial and modifiable aspect of anticancer therapeutic response. Microbiome modulation (diet, probiotics, FMT, targeted antibiotics, engineered bacteria, phagotherapy) has great potential to optimize chemotherapy
A gut microbial signature for combination immune checkpoint blockade across cancer types	Gunjur A et al. (2024)	Australia, USA, UK, Netherlands, Spain	Cohort study, meta-analysis	To discover a strain-level gut microbiome signature associated with response to combination immune checkpoint inhibition (ICI) (ipilimumab + nivolumab) in a diverse cohort of rare cancers and validate its generalizability	Strain-level microbial signatures (AUC = 0.73 for RvsP, AUC = 0.70 for PFS12) outperform clinical predictors (AUC = 0.56 for RvsP, AUC = 0.65 for PFS12) and higher taxonomic levels. *Faecalibacterium* sp. and *Bifidobacterium longum* associated with better response. External generalization better for concordant ICI regimens (anti-PD-1 or anti-PD-1 + anti-CTLA-4 combination) (median AUC = 0.65 for combination) than discordant (median AUC = 0.51 for anti-PD-1 monotherapy)	Strain-level gut microbial signatures are more predictive of combination ICI response than clinical factors. ICI regimen specificity is crucial, suggesting that microbiome biomarkers should be tailored to the treatment, not just the cancer type
Crosstalk between the gut microbiome and clinical response in locally advanced thoracic esophageal squamous cell carcinoma during neoadjuvant camrelizumab and chemotherapy	Xu L et al. (2022)	China	Clinical trial, prospective cohort study	To explore the interaction between the gut microbiome and clinical response, as well as adverse events (AEs), in patients with locally advanced Esophageal Squamous Cell Carcinoma (ESCC) undergoing neoadjuvant camrelizumab and chemotherapy	Alpha diversity of the microbiome decreased after neoadjuvant therapy and surgery. Pseudomonadales enriched in pathological non-responders (non-pCR and non-MPR). Barnesiellaceae, *Pyramidobacter*, *Butyricimonas*, *Prevotella*, *Barnesiella*, and *Odoribacter* enriched in pathological responders (MPR). Patients with ≥ Grade 3 AEs showed enrichment of *Succiniclasticum*, *Nakamurella*, *Rhizobium*, *Granulicella*, *Staphylococcus*, among others. *Phascolarctobacterium*, Odoribacteraceae, Synergistes, *Butyricimonas*, *Odoribacter*, and *Anaerotruncus* associated with Grade 1–2 AEs. *Lactobacillales*, *Abiotrophia*, and *Phascolarctobacterium* enriched in patients with PD-L1 CPS ≥ 10. *Fusobacterium* enriched in patients with clinical stage III-IVa	Gut microbiome diversity and composition change during neoadjuvant camrelizumab and chemotherapy in ESCC. Taxonomic features of the microbiome can be potential biomarkers to predict pathological response and adverse events
Role of gut microbiome in neoadjuvant chemotherapy response in urothelial carcinoma: a multi-institutional prospective cohort evaluation	Bukavina L et al. (2024)	USA	Prospective cohort study	To examine gut microbiome variation in urothelial carcinoma (UC) patients and investigate compositional differences between responders and non-responders to neoadjuvant chemotherapy (NAC)	UC patients showed higher prevalence of *Prevotella* (4.35% vs. 0.17%) and *Porphyromonas* (1.37% vs. 0.02%) and lower abundance of *Faecalibacterium* (4.66% vs. 6.19%) versus controls (*p* < 0.05). Alpha diversity was higher in the UC group (*p* < 0.001). No significant differences in alpha/beta diversity between responders (CR) and non-responders (NR) to NAC. NR showed higher abundance of *Bacteroides* (26.95% vs. 18.93%) and *Pseudomonas* (2.18% vs. 1.67%) (*p* < 0.05). Random forest model for CR achieved an AUC of 0.88, with *Oscillibacter*, *Fusicatenibacter*, and *Bacteroides* as the most important predictors	The gut microbiome is associated with NAC response in UC, with increased *Bacteroides* being a negative prognostic factor. Microbiome-based predictive models outperform clinical variables in predicting CR, suggesting the potential for a complex microbial signature
A phase II randomized clinical trial and mechanistic studies using improved probiotics to prevent oral mucositis induced by concurrent radiotherapy and chemotherapy in nasopharyngeal carcinoma	Xia C et al. (2021)	China	Randomized clinical trial, mechanistic study	To evaluate the efficacy of an improved probiotic cocktail in preventing oral mucositis (OM) induced by concurrent chemoradiotherapy (CCRT) in nasopharyngeal carcinoma (NPC) patients and investigate underlying mechanisms	The probiotic cocktail significantly reduced OM severity. Grade 3–4 OM rates: 47.1% in placebo vs. 25% in probiotic group (*p* < 0.01). The probiotic cocktail significantly increased the reduction rate of CD3 + T cells (75.5% vs. 81%, *p* < 0.01), CD4 + (64.53% vs. 79.53%, *p* < 0.01), and CD8 + (75.59% vs. 62.36%, *p* < 0.01) compared to placebo. In rats, the cocktail attenuated inflammation (IL-6, IL-1β, TNF-α), apoptosis (Bax/Bcl-2), and restored intestinal barrier integrity (ZO-1, Claudin-1), normalizing dysbiosis	The modified probiotic cocktail effectively reduces the severity of CCRT-induced OM in NPC patients by modulating gut dysbiosis and strengthening the immune response
Grow with the challenge—microbial effects on epithelial proliferation, carcinogenesis, and cancer therapy	von Frieling J et al. (2018)	Germany	Review	To provide an overview of how bacterial signals and signatures can influence epithelial homeostasis, carcinogenesis, and the success of cancer therapy	The gut microbiome affects cell proliferation and cell death, being crucial for tissue homeostasis. Dysbiosis (e.g., *Fusobacterium nucleatum*, *Bacteroides fragilis*) promotes carcinogenesis and chemoresistance (gemcitabine, 5-FU). Specific microbiomes (e.g., *Lactobacillus johnsonii*, *Enterococcus hirae*, *Barnesiella intestinihominis*) are essential for cyclophosphamide and oxaliplatin efficacy. *Bifidobacterium* promotes anti-PD-L1 response	The microbiome is a crucial regulator of tissue homeostasis, carcinogenesis, and cancer therapy efficacy. Modulating its composition and activity represents a promising approach to prevent carcinogenesis and enhance the success of chemotherapy and immunotherapy
The relationship between gut microbiome features and chemotherapy response in gastrointestinal cancer	Li N (2021)	China	Prospective cohort study	To identify specific bacteria associated with chemotherapy response in advanced gastrointestinal (GI) cancer patients, monitoring fecal microbiome changes	GI cancer patients showed higher abundance of *Bacteroides fragilis*, *Escherichia coli*, *Akkermansia muciniphila*, *Clostridium hathewayi*, and *Alistipes finegoldii*, and lower abundance of *Faecalibacterium prausnitzii*, *Roseburia faecis*, *Clostridium clostridioforme*, *Blautia producta*, *Bifidobacterium adolescent*, and *Butyricicoccus pullicaecorum*, compared to healthy controls. The abundance of *Roseburia faecis* in the NR group significantly decreased after chemotherapy, while it increased in the R group (*p* = 0.024). In esophageal cancer (EC), reduced *R. faecis* abundance was associated with disease progression (PD). The ROC model for *R. faecis* abundance variation to differentiate PD/non-PD had an AUC = 0.818 (sensitivity = 75.0%, specificity = 93.9%)	The change in *Roseburia faecis* abundance can be a predictor of chemotherapy efficacy in GI cancer patients, suggesting that the microbiome profile has prognostic value
Gut microbiome with RAS mutation and chemotherapy response in patients with advanced or metastatic colorectal cancer: a pilot, exploratory study	Kim JH et al. (2025)	South Korea	Pilot and exploratory prospective cohort study	To investigate gut microbiome characteristics in relation to lifestyle factors, clinicogenomic factors, and chemotherapy responses in metastatic colorectal cancer (mCRC) patients	Lifestyle factors: smokers had higher prevalence of *Actinomyces* and *Solobacterium*. RAS mutation: beta diversity (Bray–Curtis *P* = 0.042, weighted UniFrac *P* = 0.047) differed. *Holdemanella*, *Anaerostipes*, and *Collinsella* enriched in RAS-mutated patients. Chemotherapy response: responders had more *Lactobacillus* spp. at baseline. Disease control: *Bifidobacterium* significantly increased in non-PD, *Bacteroides* increased in PD (*P* = 0.2065, not significant for *Bacteroides*)	The gut microbiome is associated with smoking history, RAS mutation status, and chemotherapy response in mCRC. There is potential for the microbiome to act as a predictive biomarker of response and prognosis, with its composition shaped by host factors and genetic mutations
Gut microbiome model predicts response to neoadjuvant immunotherapy plus chemoradiotherapy in rectal cancer	Yang Z et al. (2024)	China	Prospective, Multicenter Cohort Study	To develop and validate a species-level gut microbiome predictive model for pathological Complete Response (pCR) to neoadjuvant immunoradiotherapy (nICRT) in Locally Advanced Rectal Cancer (LARC) patients	The pCR rate was 42.4% (14/33). *Lactobacillales* and *Eubacterium* increased after nICRT. Responders (Rs) showed higher abundance of *Lachnospiraceae bacterium* and *Blautia wexlerae* at baseline. Non-responders (NRs) showed higher abundance of *Bacteroides*, *Prevotella*, and *Porphyromonas*. The species-level predictive model (SPEED) achieved an AUC of 98.80% (95% CI 95.67%–100%) in training and 77.78% (95% CI 65.42%–88.29%) in validation	The SPEED model can predict pCR to nICRT in LARC with high robustness and accuracy based on baseline species-level gut microbiome, aiding individualized patient management
Gut microbiota modulation of efficacy and toxicity of cancer chemotherapy and immunotherapy	Chrysostomou D et al. (2023)	UK	Review	To review current knowledge on oncological pharmacomicrobiomics, describing how the multifaceted functions of the gut microbiome influence treatment response in various cancer types	Microbial enzymes (e.g., β-glucuronidases) transform chemotherapeutics (irinotecan) into toxic forms. Intratumoral Gram-negative bacteria (γ-proteobacteria) inactivate gemcitabine. Intratumoral *Bifidobacterium* increases type I IFN. Cyclophosphamide depends on Gram-positive bacteria (*Lactobacillus johnsonii*, *Enterococcus hirae*) for Th17/Th1 immune responses. Microbiome impacts ICIs: *Bacteroides fragilis*, *B. thetaiotaomicron* mediate anti-CTLA-4; *Bifidobacterium* enhances anti-PD-L1. Microbial metabolites (SCFAs, inosine) influence ICI efficacy/toxicity	The gut microbiome possesses a vast enzymatic arsenal that profoundly influences the efficacy and toxicity of chemotherapy and immunotherapy. Microbiome manipulation (diet, probiotics, antibiotics, FMT) is a promising strategy to optimize oncological outcomes and reduce toxicity
Gut microbiota-mediated nucleotide synthesis attenuates the response to neoadjuvant chemoradiotherapy in rectal cancer	Teng H et al. (2023)	China	Prospective cohort study, meta-analysis, in vitro/in vivo functional validation	To decipher the underlying mechanism of heterogeneous response in Locally Advanced Rectal Cancer (LARC) patients to neoadjuvant chemoradiotherapy (nCRT), focusing on gut microbiome-derived metabolites	Fecal microbial diversity decreased after nCRT (*p* < 0.001). Nucleotide synthesis mediated by *Bacteroides vulgatus* and DNA repair were enriched in non-responders. Nucleoside supplementation or gavage with *B. vulgatus* protected tumor cells (HCT116, MC38) from 5-FU and irradiation in vitro/in vivo. Increased serum uric acid levels were a prognostic marker of worse response (*p* = 0.0016; AUC = 0.732)	Gut microbiota-mediated nucleotide synthesis attenuates nCRT response in LARC. Serum uric acid is a potential prognostic biomarker. Deciphering communication between cancer cells and gut microorganisms is crucial to understand therapeutic resistance
AENEAS: a randomized phase iii trial of aumolertinib versus gefitinib as first-line therapy for locally advanced or metastatic non–small-cell lung cancer with EGFR exon 19 deletion or L858R mutations	Lu S et al. (2022)	China	Randomized, double-blind phase III clinical trial	To evaluate the efficacy and safety of aumolertinib compared to gefitinib as first-line therapy for locally advanced or metastatic Non–Small-Cell Lung Cancer (NSCLC) with activating EGFR mutations (exon 19 deletion or L858R)	Significantly longer PFS with aumolertinib vs. gefitinib (HR = 0.46; 95% CI 0.36 to 0.60; *p* < 0.0001). Median PFS: 19.3 months (aumolertinib) vs. 9.9 months (gefitinib). ORR and DCR similar between groups (ORR 73.8% vs. 72.1%; DCR 93.0% vs. 96.7%). Median duration of response (DoR), 18.1 months (aumolertinib) vs. 8.3 months (gefitinib) (HR = 0.38; *p* < 0.0001). Grade ≥ 3 adverse events (AEs) similar (36.4% vs. 35.8%)	Aumolertinib is a well-tolerated and effective third-generation EGFR tyrosine kinase inhibitor, which can serve as a first-line treatment option for EGFR-mutated NSCLC
Fecal microbiota transplant overcomes resistance to anti–PD-1 therapy in melanoma patients	Davar D et al. (2021)	USA	Clinical trial, prospective cohort study	To evaluate the safety and efficacy of fecal microbiota transplant (FMT) derived from responders, along with anti-PD-1, in PD-1 refractory melanoma patients, to overcome resistance	FMT was well-tolerated. Objective response (OR) in 3/15 patients (20%), and durable stable disease (SD) in 3/15 patients (20%). Median PFS of 14.0 months and median OS of 14.0 months in responders (OR + SD). FMT induced persistent disruption of the gut microbiome. Responders exhibited increased taxa previously associated with response (e.g., Firmicutes, Actinobacteria), greater CD8 + T cell activation, and decreased frequency of IL-8 expressing myeloid cells	FMT and anti-PD-1 overcame anti-PD-1 resistance in a subset of PD-1 refractory melanoma patients, reprogramming the tumor microenvironment and inducing favorable clinical and immunological responses
Intestinal microbiota signatures of clinical response and immune-related adverse events in melanoma patients treated with anti-PD-1	McCulloch JA et al. (2022)	USA	Cohort Study, Meta-analysis	To evaluate novel and published melanoma cohorts to identify microbial signatures predicting clinical response and immune-related adverse events (irAEs) to anti-PD-1 therapy	Baseline microbial composition was associated with PFS at ~ 1 year after treatment initiation (distance = 0.32, *p* = 0.006). Taxa associated with better response include Actinobacteria and the Lachnospiraceae family. Unfavorable taxa include Bacteroidetes and Proteobacteria, correlated with systemic inflammation (elevated NLR). Two microbial signatures (*Lachnospiraceae* spp. and *Streptococcus* spp.) were associated with favorable/unfavorable clinical outcomes and distinct irAEs. Machine learning (ML) models consistently predicted outcomes (AUC > 0.79 in some combinations)	Gut microbial signatures predict clinical response and irAEs to anti-PD-1 therapy in melanoma. There is a strong association between unfavorable taxa (Gram-negatives) and inflammation, and the distinction between favorable/unfavorable microbiotypes may explain discrepancies between cohorts
Gut OncoMicrobiome Signatures (GOMS) as next-generation biomarkers for cancer immunotherapy	Thomas AM et al. (2023)	Italy, France, Canada, USA	Systematic review, meta-analysis, mega-analysis	To discuss how GOMS are shared among cancer subtypes and inflammatory diseases, and to summarize knowledge on their effect on cancer-related states and their utility as biomarkers in cancer immunotherapy, focusing on ICIs	Variations in the gut microbiome (GOMS) are associated with aging, chronic inflammation, and medication use (e.g., PPIs, antibiotics). Pan-cancer GOMS (e.g., enrichment of *Enterocloster*, *Hungatella*, *Clostridium*, and depletion of Lachnospiraceae, Oscillospiraceae) differ from healthy individuals. *A. muciniphila* (< 4.8%) associated with inflamed tumor microenvironment and better outcomes in NSCLC (ORR and OS). ICI responders (meta-analysis of 808 patients) had enrichment of Lachnospiraceae, Ruminococcaceae, *Bifidobacterium*, and depletion of *Enterocloster*, Veillonellaceae. SCFA, Kyn/Trp, L-Arginine, and TMAO levels are microbial metabolites that influence ICI response	GOMS represent promising and non-invasive predictive biomarkers for cancer immunotherapy. Patient stratification based on gut dysbiosis and microbiome-centric interventions can optimize ICI efficacy
Gut microbiome development along the colorectal adenoma–carcinoma sequence	Feng Q et al. (2015)	Austria, China	Cross-sectional metagenomic-wide association study (MGWAS)	To identify microbial genes, strains, and functions enriched in fecal samples from patients with advanced adenoma and colorectal carcinoma (CRC) compared to healthy controls, and to explore the impact of risk factors like diet	Significantly higher gene and genus richness in adenoma and carcinoma versus control (*p* < 0.005). Carcinoma-characteristic MLGs (metagenomic linkage groups): *Bacteroides*, *Parabacteroides*, *Alistipes putredinis*, *Bilophila wadsworthia*, *Lachnospiraceae bacterium*, *Escherichia coli* enriched. Control-characteristic MLGs: *Bifidobacterium animalis*, *Streptococcus thermophilus*. Random forest classifier for carcinoma achieved AUC of 96%; for adenoma, AUC of 87.38%. Red meat consumption positively correlated with bacteria enriched in carcinoma	The gut microbiome undergoes profound changes along the colorectal adenoma–carcinoma sequence. Fecal microbial signatures have great potential for early and non-invasive diagnosis of adenoma and colorectal carcinoma. Diet exerts a significant influence on cancer-associated microbiota composition
The influence of the gut microbiome on cancer, immunity, and cancer immunotherapy	Gopalakrishnan V et al. (2018)	USA	Review	To provide a comprehensive understanding of the intricate relationship between the microbiome, immunosurveillance, and cancer, and how these factors, including gut microbiota, influence immunotherapy response	The microbiome modulates immunity (local, systemic) and intestinal homeostasis. Dysbiosis (e.g., in hematopoietic stem cell transplantation (HSCT)) associated with lower survival and higher toxicity (e.g., graft-versus-host disease (GVHD)). *Blautia* abundance associated with reduced GVHD lethality. In ICIs, the microbiome influences response: *Bacteroides fragilis* enhances anti-CTLA-4 action; *Bifidobacterium* increases anti-PD-1. In melanoma patients treated with anti-PD-1, higher microbial diversity and abundance of Clostridiales, Ruminococcaceae, and *Faecalibacterium* associated with response	The microbiome is a crucial regulator of tissue homeostasis, carcinogenesis, and cancer therapy efficacy. Modulating its composition and activity represents a promising approach to prevent carcinogenesis and enhance the success of chemotherapy and immunotherapy
Impact of the gut microbiome on response and toxicity to chemotherapy in advanced esophageal cancer	Li N et al. (2024)	China	Prospective cohort study	To identify specific gut bacteria associated with chemotherapy (TP regimen: paclitaxel and cisplatin) response and toxicity in advanced esophageal squamous cell carcinoma (ESCC) patients	*Bacteroides plebeius* was significantly higher in partial responders (PR) (*p* = 0.043) and overall responders (R) (*p* = 0.045) at baseline. *B. ovatus* predicted PR with 83.3% sensitivity and 69.6% specificity (AUC = 0.790, *p* = 0.031). *B. plebeius* predicted R with AUC = 0.865 (*p* = 0.041). *B. plebeius* and *B. uniformis* were predictors of Grade 3–4 toxicity. Multiparametric model (*B. plebeius* + *B. uniformis*) for toxicity achieved AUC = 0.825, *p* = 0.011 (sensitivity = 85.7%, specificity = 77.3%)	The abundance of *Bacteroides plebeius* and *Bacteroides ovatus* is associated with TP chemotherapy efficacy in ESCC. *B. plebeius* and *B. uniformis* can be indicators of Grade 3–4 toxicity, suggesting a promising role as predictive biomarkers
Regorafenib plus toripalimab in patients with metastatic colorectal cancer: a phase Ib/II clinical trial and gut microbiome analysis	Wang F et al. (2021)	China	Phase Ib/II clinical trial, prospective cohort study	To evaluate the tolerability, safety, preliminary efficacy, and gut microbiome related to efficacy of regorafenib plus toripalimab for refractory Metastatic Colorectal Cancer (mCRC) patients with Microsatellite Stability (MSS)/Proficient Mismatch Repair (pMMR)/Low Microsatellite Instability (MSI-L)	Objective response rate (ORR) of 15.2% and disease control rate (DCR) of 36.4%. Median PFS of 2.1 months and median OS of 15.5 months. Patients with liver metastasis had lower ORR (8.7% vs. 30.0% without). *Fusobacterium* significantly increased in non-responders. Patients with high *Fusobacterium* abundance had shorter PFS (2.0 vs. 5.2 months; *p* = 0.002)	The combination of regorafenib plus toripalimab shows manageable safety and preliminary efficacy in refractory mCRC. The gut microbiome, especially *Fusobacterium* abundance, can be a predictive biomarker of response and survival

Geographically, scientific production is remarkably diverse and collaborative. China, including Hong Kong, stands out with a significant presence in 11 of the 20 articles [15, 16, 18–25], highlighting the country's significant investment in this research area. The United States of America (USA) also plays a central role, contributing to 7 articles [12, 17, 26–30], often in international collaborations. Other countries contributing individually or in consortia to this sample include Australia [12, 30], the United Kingdom [30, 31], Germany [32], Italy [28], Canada [28], and South Korea [16], in addition to participations in meta-analyses that aggregate data from the Netherlands, Spain, and Austria [15, 30].

The methodology of the studies is equally varied, reflecting the complexity of the theme. Six of the 20 articles, representing 30% of the sample, are review studies or systematic reviews, such as the work by Gazzaniga and Kasper (2025) [17] on the response to immune checkpoint inhibitors and the review by Chrysostomou et al. (2023) [31] on the modulation of chemotherapy and immunotherapy efficacy and toxicity. These provide a consolidated view of existing knowledge. Primary research is substantially represented by 8 articles (40% of the sample) classified as prospective cohort studies, such as the study by Xu et al. (2022) [20] on esophageal squamous cell carcinoma and the evaluation by Bukavina et al. (2024) [27] on urothelial carcinoma. Four articles (20%) are clinical trials, ranging from Phase Ib/II/III, such as the study by Xia et al. (2021) [19] on probiotics in oral mucositis and the AENEAS trial by Lu et al. (2022) [24] on aumolertinib in lung cancer. Finally, two articles (10%) consist of meta-analyses or mega-analyses, such as that by Gunjur et al. (2024) [30] on microbial signatures at the strain-level and the work by McCulloch et al. (2022) [12] in melanoma patients, demonstrating the effort to synthesize large volumes of data.

The investigation into the mechanisms and impacts of the microbiome is carried out at different levels of complexity. All 20 articles in the sample (100%) include the analysis of human patient data, highlighting the direct clinical relevance of the research. The scale of these cohorts is impressive, with meta-analysis and mega-analysis studies, such as that by Thomas et al. (2023) [28], which covered data from thousands of patients and controls, while prospective cohort studies vary, for example, from 15 patients in Kim et al. (2025) [16] to 142 patients in Bukavina et al. (2024) [27] and 429 patients in Lu et al.'s Phase III trial (2022) [24]. To deepen the elucidation of biological mechanisms, 6 articles (30%) integrated animal models into their investigations [19, 22, 25, 27, 29, 32], using rats, mice, and even Drosophila. Furthermore, 2 articles (10%) employed in vitro studies with cellular models for functional validation [19, 25].

The types of cancer studied cover a wide range of malignancies, reflecting the universality of the microbiome-host interaction. Colorectal cancer (CRC) is the most frequently addressed, being investigated in 10 articles (e.g., Feng et al., 2015; Kim et al., 2025; Li et al., 2021; Teng et al., 2023; Wang et al., 2021) [15, 16, 18, 23, 25]. Melanoma appears in 8 articles (e.g., Davar et al., 2021; McCulloch et al., 2022; Thomas et al., 2023; Gopalakrishnan et al., 2018; Gunjur et al., 2024) [12, 26, 28–30], and non-small cell lung cancer (NSCLC) in 6 articles (e.g., Lu et al., 2022; Thomas et al., 2023; Gunjur et al., 2024) [24, 28, 30]. Other notable cancers include esophageal squamous cell carcinoma (ESCC) in 4 articles (e.g., Xu et al., 2022; Li et al., 2024) [18, 20], urothelial carcinoma in 3 articles (e.g., Bukavina et al., 2024; Gunjur et al., 2024) [27, 30], and hepatocellular carcinoma in 3 articles (e.g., Thomas et al., 2023; Gunjur et al., 2024) [28, 30].

Regarding microbiome analysis techniques, the sample employs the most advanced approaches. 16S rRNA sequencing is a prevalent technique, used in 11 articles (e.g., Xu et al., 2022; Bukavina et al., 2024; Li et al., 2021; Kim et al., 2025; Yang et al., 2024; Li et al., 2024; Wang et al., 2021; Xia et al., 2021; Teng et al., 2023) [16, 18–21, 23, 25, 27, 33], while metagenomic (shotgun) sequencing, which offers higher resolution at the species level and allows for functional analysis, is employed in 9 articles (e.g., Feng et al., 2015; Gunjur et al., 2024; McCulloch et al., 2022; Thomas et al., 2023; Gopalakrishnan V, 2018; Davar D, 2021) [12, 15, 26, 28–30]. Advanced bioinformatics is ubiquitous, with 8 articles using machine learning algorithms (e.g., Random Forest, SVM) and LEfSe analyses for the construction of predictive models and identification of biomarkers (e.g., Bukavina et al., 2024; Gunjur et al., 2024; Yang et al., 2024; McCulloch et al., 2022) [12, 21, 27, 30]. Furthermore, 4 articles delve into metatranscriptomics and metabolite analysis (e.g., Li et al., 2024; Teng et al., 2023; Chrysostomou et al., 2023) [22, 25, 31, 33], to capture the functional activity and products resulting from the microbiome-host-treatment interaction.

## Discussion

This discussion synthesizes the findings of 20 scientific articles published between 2015 and 2025, providing an in-depth analysis of the potential for clinical application of predictive models based on the gut microbiome in the practice of oncological treatment response. The examined literature, characterized by its methodological and geographical diversity, demonstrates that the gut microbiome exerts a multifaceted and clinically relevant influence on modulating the efficacy and toxicity of various oncological therapeutic modalities.

Among these 20 articles, a subset directly developed or validated microbiome-based predictive models with formal performance metrics (e.g., AUC, sensitivity, specificity), whereas others primarily described microbial signatures associated with treatment response, toxicity, prognosis or diagnostic accuracy without specifying a full predictive model. For clarity, in the synthesis below we distinguish, whenever possible, between (i) studies that propose or validate quantitative predictive models and (ii) studies that identify clinically relevant microbiome signatures.

One of the pillars of this analysis lies in the ability of the gut microbiome to influence therapeutic efficacy in oncology. In the context of immunotherapy, particularly with immune checkpoint inhibitors (ICIs), specific microbial compositions have been shown to modulate host response. Reviews such as Gazzaniga and Kasper (2025) [17] and Gopalakrishnan et al. (2018) [29] consolidate the association of taxa such as *Bifidobacterium*, *Akkermansia muciniphila*, *Faecalibacterium*, *Ruminococcaceae*, and *Lachnospiraceae* with more favorable ICI outcomes in various cancer types. The granularity of the analysis is crucial; Gunjur et al. (2024) [30], in a multicenter study that included 106 patients in a discovery cohort and 364 in a meta-analysis, evidenced that microbial signatures at the strain-level present superior predictive power for the response to combined ICIs. Their model achieved an AUC of 0.73 for Response vs. Progression (RvsP) and 0.70 for 12-month Progression-Free Survival (PFS12), in contrast to AUCs of 0.56 for RvsP and 0.65 for PFS12 obtained with clinical factors. However, the generalization of these biomarkers demonstrated greater robustness for concordant ICI regimens, with an average AUC of 0.65, compared to discordant regimens, which showed an average AUC of 0.51, indicating significant specificity to the treatment regimen. Additionally, microbiome modulation through Fecal Microbiota Transplantation (FMT) was investigated as a strategy to overcome ICI resistance. Davar et al. (2021) [26], in a Phase II clinical trial with 15 melanoma patients refractory to PD-1, observed that FMT resulted in objective response in 20% (3/15) of patients and disease stability in another 20% (3/15), with a median PFS of 14 months for responders. In contrast, the use of antibiotics has been associated with abrogating the anti-tumor effects of ICIs, as reviewed by Gazzaniga and Kasper (2025) [17], highlighting the delicate ecological interdependence.

In the area of chemotherapy and chemoradiotherapy, the microbiome also plays a fundamental role. Li et al. (2024) [33], in their review, indicate that *Fusobacterium nucleatum* can promote chemoresistance, while *Akkermansia muciniphila* can improve the efficacy of certain chemotherapeutic agents. The negative impact of *Fusobacterium* is corroborated by Wang et al. (2021) [23], who, in a Phase Ib/II clinical trial with 32 patients with metastatic colorectal cancer, observed a significantly shorter PFS (2.0 months vs. 5.2 months; *p* = 0.002) in patients with high abundance of this taxon. In urothelial carcinoma, Bukavina et al. (2024) [27] documented that the elevation of Bacteroides correlated with a worse response to neoadjuvant chemotherapy, while the microbiome-based predictive model achieved an AUC of 0.88, outperforming clinical factors (AUC = 0.50). In summary, the gut microbiome presents transformative potential for precision oncology, offering new approaches for patient stratification, therapy optimization, and toxicity minimization. Predictive models demonstrate superiority over traditional clinical markers. In summary, the gut microbiome presents a promising avenue for clinical biomarker development, offering potential approaches for patient stratification. However, these findings must be interpreted with caution; as noted in the cited literature, such conclusions are currently exploratory and often supported by limited models derived from small cohorts (e.g., n = 33 patients), requiring large-scale prospective validation before claiming broad clinical superiority. The complexity of the microbiome's impact is further illustrated by the identification of nucleotide synthesis mediated by *Bacteroides vulgatus* as an attenuating factor of the response to chemoradiotherapy in LARC, a mechanism elucidated by Teng et al. (2023) [25]. Longitudinal microbial dynamics also prove relevant; Li et al. (2021) [18], in a prospective cohort study with 130 gastrointestinal cancer patients, identified the variation in *Roseburia faecis* abundance as a predictor of efficacy, with an ROC model achieving an AUC = 0.818 to differentiate disease progression.

Beyond efficacy, the microbiome demonstrates a crucial role in modulating treatment toxicity. The mitigation of adverse events (AEs) induced by chemotherapy represents a significant advance for patient quality of life. Xia et al. (2021) [19], in a Phase II clinical trial with 70 patients, documented that a probiotic cocktail reduced the severity of Grade 3–4 oral mucositis (OM) from 47.1% to 25% (p < 0.01). This intervention study was integrated into our scoping framework as a functional proof-of-concept to complement observational data, maintaining a clear distinction between interventional and observational evidence during synthesis. Patients undergoing TP chemotherapyTP chemotherapy (Taxane and Platinum-based regimen, specifically Paclitaxel and Cisplatin) exhibited distinct profiles. This toxicity was further characterized by '’oralization’the ectopic translocation and enrichment of oral cavity-associated bacteria (such as Fusobacterium and Veillonella species) into the gut microbiota ecosystem. In ESCC, Li et al. (2024) [33] identified *Bacteroides plebeius* and *Bacteroides uniformis* as predictors of Grade 3–4 toxicity to TP chemotherapy, with a multiparametric model achieving an AUC of 0.825 to predict severe toxicity. In the context of immunotherapy, McCulloch et al. (2022) [12] observed that *Streptococcus* spp. were associated with immune-related adverse events (irAEs) and worse PFS in melanoma patients, and that the use of proton pump inhibitors (PPIs) correlated with the "oralization" of the microbiome and a worse prognosis (HR = 3.62, *p* = 0.0073 for PFS).

The emergence of the microbiome as a predictive and prognostic biomarker constitutes one of the greatest clinical potentials. Predictive models microbiome-based on the microbiome are demonstrating superiority over traditional clinical markers. Yang et al. (2024) [21], in a multicenter prospective cohort study, developed the SPEED (Species-level Gut Microbiome Prediction) model, which achieved an AUC of 98.80% in the training cohort and 77.78% in the validation cohort to predict pathological complete response (pCR) to neoadjuvant immunoradiotherapy in LARC. The variation in microbial composition can also predict the course of the disease, as illustrated by Kim et al. (2025) [16] in mCRC, who observed that chemotherapy responders had more *Lactobacillus* spp. and that *Bifidobacterium* increased in patients without progression, while *Bacteroides* tended to increase with disease progression. In diagnosis and staging, Feng et al. (2015) [15] demonstrated that classifiers based on Metagenomic Linkage Groups (MLGs) achieved AUCs of 96% for carcinoma and 87.38% for adenoma, indicating a potential for non-invasive early detection.

The biological mechanisms underlying these interactions are multifaceted, involving immunomodulation through the activation of T cells, NK cells, and immune signaling pathways, as well as drug metabolism and microbial metabolites that can activate or inactivate chemotherapeutics or influence inflammatory and DNA repair processes [22, 25, 31, 32].

Despite the undeniable and promising potential, the clinical translation of microbiome-based predictive models still faces gaps and challenges. One of the main ones is the lack of standardization and reproducibility. Methodological heterogeneity among studies, including sample collection, DNA extraction, and bioinformatic analyses, contributes to inconsistency in the identification of microbial biomarkers, as observed by McCulloch et al. (2022) [12] and Gunjur et al. (2024) [30]. This highlights the urgent need for multicenter studies with harmonized protocols and rigorous incorporation of confounding factors such as diet and comorbidities [16, 33].

Another critical gap is the need for greater elucidation of causal mechanisms and functional validation. Many current findings establish only associations, and the transition from correlation to causality requires robust functional validation in preclinical and in vitro models [27, 33]. Strain-level resolution is fundamental, as different strains can have divergent functions, and the role of non-bacterial microorganisms (fungi, viruses) is still insufficiently explored [22, 28].

Multimodal data integration and understanding the spatio-temporal dynamics of the microbiome are also crucial aspects. More comprehensive predictive models should incorporate tumor and host genomic data, clinical information, and microbial metabolites [28]. The variation of the microbiome over time and across different anatomical compartments requires more longitudinal studies [20, 25].

Finally, clinical translation and regulatory challenges are substantial barriers. The development of live biotherapeutic products (LBPs) and their standardization, the cost-effectiveness of high-resolution metagenomics, and the need for rapid and accessible tests for routine clinical practice represent significant challenges that need to be addressed for the full potential of the microbiome in oncology to be realized [17, 31].

### Methodological fragilities and sources of bias in current evidence

Despite the promising results reported across studies, several methodological fragilities limit the robustness and generalizability of current microbiome-based predictive models. Many studies relied on relatively small sample sizes in relation to the high dimensionality of microbiome data, increasing the risk of overfitting, particularly in machine learning approaches. External validation was infrequently performed, and when present, often demonstrated reduced predictive performance. Additionally, important confounding factors, such as diet, antibiotic and proton pump inhibitor use, lifestyle habits, and geographic variability, were inconsistently controlled or reported. These limitations underscore that most available evidence remains associative rather than causal, reinforcing the need for rigorously designed, multicenter, longitudinal studies with harmonized protocols.

In summary, the gut microbiome presents transformative potential for precision oncology, offering new approaches for patient stratification, therapy optimization, and toxicity minimization. Future research, focused on standardization, causal elucidation, and multimodal data integration, will pave the way for the effective implementation of these predictive models in clinical practice.

### Specific challenges in microbiome-based predictive modeling

Beyond general methodological issues, several challenges are specific to the development of robust microbiome-based predictive models in oncology. Many studies face an unfavorable ratio between sample size and the high dimensionality of microbiome features, which increases the risk of overfitting, particularly in machine learning approaches. Internal validation is often limited to simple train–test splits or cross-validation, and external validation in independent cohorts remains rare; when performed, a marked drop in performance is frequently observed. Few studies report model calibration or compare microbiome-only models against models including established clinical and molecular predictors. Moreover, model interpretability is seldom addressed, despite its importance for clinical adoption, especially when complex algorithms (e.g., random forests, support vector machines, deep learning) are used. Future studies should adhere to reporting guidelines for prediction models (e.g., TRIPOD), transparently describe feature selection and validation strategies, and prioritize multicenter designs with harmonized protocols and predefined external validation cohorts.

## Conclusion

This scoping review, by synthesizing the literature comprising 20 recent studies, shows that the gut microbiome holds substantial but still emergent clinical potential as a biomarker in oncology. The analyzed literature indicates that microbiome-based predictive models and microbial signatures can improve patient stratification, support the optimization of therapeutic interventions, and help anticipate treatment-related toxicities by identifying microbial patterns correlated with favorable or adverse clinical outcomes. However, this promise is accompanied by critical gaps that require systematic attention. The evidence reveals notable methodological heterogeneity across studies, from sample collection and processing to sequencing and bioinformatic pipelines, which compromises the standardization and reproducibility of microbial biomarkers and poses challenges for translation into clinical practice. Most available data remain associative rather than causal, underscoring the need for robust functional validation and mechanistic studies, preferably at the strain-level, to deepen our understanding of microbiome functionality. Furthermore, the integration of microbiome data with other molecular and clinical biomarkers, in multimodal and longitudinal study designs, is fundamental for developing comprehensive and clinically applicable predictive models that move beyond simple correlations. Collectively, these considerations emphasize that the gut microbiome is a promising component of precision oncology, but its routine clinical implementation will depend on methodological standardization, rigorous validation, and clear demonstration of added value over existing clinical tools.

## Supplementary Information

Below is the link to the electronic supplementary material.

Tables S1–S20: Contain the detailed data extraction for each of the 20 articles included in the review, offering comprehensive information on their characteristics, types of cancer analyzed, oncological treatment modalities, methodologies, and results, as referenced in the Results section for individual study consultation. These additional materials aim to ensure the transparency and reproducibility of the review’s findings, enabling readers to access the complete analytical data foundation.

ESM 1 (254 KB DOCX)

## Data Availability

No datasets were generated or analyzed during the current study.
