# Supplementary material for: Clinical potential of the gut microbiome in oncology: a scoping review of treatment response, toxicity and biomarker development
Source: Support Care Cancer. 2026 Jun 20;34(7):676. doi: 10.1007/s00520-026-10918-1 (PMC13283132; doi:10.1007/s00520-026-10918-1)
Supplement: Supplementary file 1 — (254 KB DOCX) [file 520_2026_10918_MOESM1_ESM.docx]

Table S1. Article 1: The gut microbiome and cancer response to immune checkpoint inhibitors

| Category | Extracted Variables | Detailing |
| --- | --- | --- |
| A. Study Identification | A1. Lead Author (Year) | Gazzaniga FS (2025) |
|  | A2. Title | The gut microbiome and cancer response to immune checkpoint inhibitors |
|  | A3. Journal | The Journal of Clinical Investigation. |
|  | A4. Country/Region | USA (Boston, Massachusetts) |
|  | A5. Funding | NIH National Cancer Institute (1K22CA258960-01, 1R21CA287104), Cancer Research Institute CLIP grant (CRI5506), Victoria’s Secret Global Fund for Women’s Cancers Career Development Award, Pelatonia and AACR (24-20-73-GAZZ), Mass General Cancer Krantz Family Center for Cancer Research Award, Department of Defense grant (HT94252310226). Type: Public, Competitive. |
|  | A6. Conflicts of interest | Yes, FSG and DLK are inventors on provisional US patent applications (62/776767 and 62/817,231) and FSG on an international application (PCT/US19/25460). |
| B. Methodological Design | B1. Study type | Review |
|  | B2. Follow-up duration | Not applicable (review) |
|  | B3. Study center(s) | Not applicable (review) |
|  | B4. Recruitment period | Not applicable (review) |
|  | B5. Ethical approval | Not applicable (review) |
| C. Study Population | C1. Sample size | Not applicable (review of previous studies) |
|  | C2. Age | Not applicable |
|  | C3. Sex | Not applicable |
|  | C4. Cancer type | Advanced cutaneous melanoma, Non-Small Cell Lung Cancer (NSCLC), Renal Cell Carcinoma (RCC), Hepatocellular Carcinoma (HCC), Thoracic Carcinoma, Urothelial Cancer, Gastrointestinal Cancers, Hepatobiliary Cancers. |
|  | C5. Tumor stage | Advanced, Treatment-resistant. |
|  | C6. Performance status | Not applicable |
|  | C7. Relevant comorbidities | Not applicable |
|  | C8. Previous treatments | Previous antibiotics (ABT), Previous ICI treatment. |
| D. Chemotherapy Protocol | D1. Chemotherapy regimen | Programmed Cell Death Protein 1 (PD-1) blocking antibodies, Programmed Cell Death Ligand 1 (PD-L1), Cytotoxic T-Lymphocyte Associated Protein 4 (CTLA-4), and Lymphocyte Activation Gene 3 (LAG3). |
|  | D2. Treatment line | Not specified (various ICI treatment contexts) |
|  | D3. Treatment intent | Immunotherapy (cancer treatment). |
|  | D4. Number of cycles | Not specified. |
|  | D5. Combination therapies | Possible combination with chemotherapies, although the focus is on microbiome and ICIs. |
|  | D6. Dose modifications | Not specified. |
| E. Microbiome Analysis | E1. Sample type | Feces (Humans and murine models), Patient samples (exact type not specified, but mentions Fecal Microbiota Transplants (FMTs)). |
|  | E2. Timing of collection | Baseline, after treatment (FMT context), before/after antibiotic use. |
|  | E3. Sequencing technique | 16S rRNA sequencing (for genus/family resolution), Metagenomic analysis (for species-level resolution and genes). |
|  | E4. Sequencing platform | Not specified (review of multiple studies). |
|  | E5. Sequencing depth | Not specified (review). |
|  | E6. Bioinformatic pipeline | Not specified (review). |
|  | E7. Reference database | Not specified (review). |
|  | E8. Quality control | Not specified. |
| F. Predictive Model | F1. Model type | Not applicable (review discusses associations, not a specific predictive model developed in this article). |
|  | F2. Algorithm(s) used | Not applicable. |
|  | F3. Predictive variables | Microbial taxa (genera, species, strains), microbial metabolites (e.g., GMDP, inosine, I3A, ICA, TMA, TMAO, butyrate), diets (e.g., high-fiber). |
|  | F4. Feature selection method | Preclinical and clinical association studies. |
|  | F5. Model validation | FMTs from responders to mice and patients. |
|  | F6. Performance metrics | Not applicable (qualitative discussion of associations). |
|  | F7. Training set size | Not applicable. |
|  | F8. Test set size | Not applicable. |
|  | F9. Model interpretability | Cladograms and molecular pathway diagrams (Figures 1 and 2 in the article). |
|  | F10. Model availability | Not applicable. |
| G. Outcomes Assessed | G1. Primary study outcome | Response to ICIs (antitumor efficacy). |
|  | G2. Response criteria | Not specified (review of multiple studies). |
|  | G3. Evaluation time | Not specified (review). |
|  | G4. Secondary outcomes | Survival (in melanoma), Toxicity (adverse events, gut dysbiosis), Antitumor immunity (CD8+ T cells). |
|  | G5. Associated clinical/laboratory biomarkers | Not specified (focus on microbiome). |
|  | G6. Associated lifestyle/medication factors | Antibiotic use, diet (high-fiber), probiotics. |
|  | G7. Associated host genetic factors | Not specified (focus on microbiome). |
|  | G8. Intervention results (if applicable) | FMTs from responders promote ICI response. Probiotics have variable effects (benefits in CRC, but worse outcomes in melanoma with some types). |
| H. Key Results and Findings | H1. Key microbial taxa/species | Promoters of ICI Response in mice: *Bifidobacterium breve*, *Bifidobacterium longum*, *Akkermansia muciniphila*, *Alistipes indistinctus*, *Enterococcus hirae*, *Enterococcus faecium*, *Enterococcus durans*, *Enterococcus mundtii*, *Coproobacillus cateniformis*, *Erysipelatoclostridium ramosum*, *Lactobacillus gallinarium*, *Lactobacillus rhamnosus GG*, *Roseburia intestinalis*, *Faecalibacterium prausnitzii*, *Bacteroides fragilis*, *Bacteroides thetaiotaomicron*, *Burkholderia cepacia*, *Bifidobacterium pseudolongum*, *Lactobacillus johnsonii*, *Olsenella* sp. Associated with ICI Response in patients: *Akkermansia muciniphila*, *Alistipes* spp., *Ruminococcus* spp., *Eubacterium* spp., *Faecalibacterium*, *B. longum*, *Collinsella aerofaciens*, *E. faecium*. Metabolites involved: GMDP (GlcNac-muramyl dipeptide), inosine, I3A (indole-3-aldehyde), ICA (indole-3-carboxylic acid), TMA (trimethylamine), TMAO (trimethylamine N-oxide), butyrate. |
|  | H2. Direction of association | The above-listed taxa and metabolites (H1) are associated with improved ICI response/efficacy. Antibiotics abrogate the response. Certain probiotics (*Bifidobacterium longum 35624*, *Lactobacillus rhamnosus GG*) may have pro- or anti-tumor effects, depending on the context. |
|  | H3. Model predictive performance | Not applicable (review). |
|  | H4. Microbial diversity findings | Cancer patients responding to ICIs have a different microbiome than non-responders. Reduced microbial diversity (e.g., after antibiotic use or in germ-free mice) generally abrogates ICI response. |
|  | H5. Microbial functional/metabolic findings | Bacterial metabolites (GMDP, inosine, I3A, ICA, TMA, TMAO, butyrate) act via NOD2, adenosine A2A receptor, AhR receptor, cGAS/STING, tumor-associated macrophages, and TLR5 to promote antitumor immunity. |
|  | H6. Preclinical/*in vitro* findings | Various bacterial species and their metabolites have demonstrated in murine models (germ-free monocolonized mice, antibiotic-pretreated mice, and oral gavage) the ability to promote or abrogate ICI response. |
|  | H7. Highlight of Findings: Unique Contribution | This review article highlights the growing evidence of gut microbiota and its metabolites modulating ICI efficacy, elucidating complex mechanisms ranging from immune cell activation to regulatory pathway suppression. The "major discovery" lies in the translation of microbiome impact from preclinical models to patients and the elucidation of metabolites and molecular pathways as potential targets for therapeutic optimization. The variability of probiotic response and the need for safer, standardized therapies is a turning point. |
|  | H8. Validation status (internal/external) | The efficacy of FMT from responders to overcome resistance has been validated in limited clinical trials (success rate of about one-third in melanoma, up to 65% in advanced melanoma with FMT before the first anti-PD-1 dose). Preclinical studies in mice confirmed the responder phenotype. |
| I. Discussion/Article Implications | I1. Authors' interpretation | The field of gut microbiome and immunotherapy is rapidly expanding, with new discoveries of mechanisms of action, bacterial species, and metabolites. The microbiome can influence antitumor immunity in a complex and variable manner. |
|  | I2. Proposed biological mechanisms | Bacteria can promote antitumor immunity via: antigen-presenting cell (APC) and myeloid cell activation (GMDP via NOD2, c-di-AMP via cGAS/STING); direct T cell activation (inosine via A2A receptor, I3A via AhR, ICA competing with kynurenine at AhR); butyrate production via TLR5; TMA metabolism to TMAO stimulating macrophages. |
|  | I3. Correlations with other biomarkers | Metagenomic analysis and qPCR-based tests of 21 bacterial strains (in 872 NSCLC, genitourinary, colorectal patients) can stratify patients with good immunotherapy survival prognosis. |
|  | I4. Impact of interventions (if applicable) | FMTs showed potential to overcome resistance, but with variable efficacy and infection risks. Probiotics had mixed effects, with some showing benefits (*Clostridium butyricum* in CRC) and others associated with worse outcomes ("off-the-shelf" probiotics in melanoma). High-fiber diet (P > 20g/day) and no use of "off-the-shelf" probiotics in melanoma were associated with longer survival. |
|  | I5. Consistency/Inconsistency of findings | Differences between studies in identifying bacteria associated with response may be due to tumor types, treatments (anti-PD-1 vs. anti-PD-L1), bacterial sources, microbiomes of mice from different facilities, sequencing methods (16S rRNA vs. metagenomics), and regional diets. |
|  | I6. Transferability/Generalization | Despite species variability, the association of the gut microbiome with immunotherapy response is a global finding, applicable to many cancer types. Clinical translation requires identification of specific bacteria and their mechanisms. |
|  | I7. Causality vs. Association | Demonstrated cause-and-effect relationship in murine models (germ-free monocolonized mice, oral gavage of bacteria). In patients, FMT from responders to mice and patients suggests causality. |
|  | I8. Implications for treatment | Development of safer, more predictable, and effective therapies, focused on specific bacterial metabolites or molecular targets, instead of "Trojan horse" FMTs with billions of live organisms. |
| J. Clinical Application Potential and Barriers | J1. Potential for implementation in practice | The identification of specific metabolites and their mechanisms allows for the development of targeted therapies that may be more standardizable and regulable than FMTs. qPCR-based tests can stratify patients. |
|  | J2. Advantages | Metabolite-based therapies can circumvent the inherent variability of live bacterial treatments. Potential for safer and standardized treatments. |
|  | J3. Barriers to implementation | FMTs are difficult to regulate and standardize, with a risk of infections. Microbiome variability in patients (diet, medications, lifestyle) affects the reproducibility of probiotic effects. |
|  | J4. Cost-effectiveness | Not directly addressed. |
|  | J5. Recommendations for future research | Understand mechanisms behind specific bacteria and their metabolites. Focus on designing more predictable and safe therapies. |
|  | J6. Personalization potential | Identification of specific microbiomes for different populations and cancer types. |
| K. Limitations and Bias of the Original Study | K1. Limitations declared by authors | Review, does not develop original study with its own limitations. |
|  | K2. Selection bias | Not applicable (review). |
|  | K3. Confounding bias | Not applicable (review). |
|  | K4. Generalization | Not applicable (review). |
|  | K5. Reproducibility | Not applicable (review). |

Table S2. Article 2: The role of gut microbiota and metabolites in cancer chemotherapy

| Category | Extracted Variables | Detailing |
| --- | --- | --- |
| A. Study Identification | A1. Lead Author (Year) | Li S (2024) |
|  | A2. Title | The role of gut microbiota and metabolites in cancer chemotherapy |
|  | A3. Journal | Journal of Advanced Research. |
|  | A4. Country/Region | China (Hong Kong) |
|  | A5. Funding | Shenzhen-Hong Kong-Macao Science and Technology Program (Category C) Shenzhen (SGDX20210823103535016); RGC Research Impact Fund Hong Kong (R4032-21F). Type: Public. |
|  | A6. Conflicts of interest | No, the authors declare no known financial or personal conflicts that could have influenced the work. |
| B. Methodological Design | B1. Study type | Review |
|  | B2. Follow-up duration | Not applicable (review) |
|  | B3. Study center(s) | Not applicable (review) |
|  | B4. Recruitment period | Not applicable (review) |
|  | B5. Ethical approval | Not applicable (review) |
| C. Study Population | C1. Sample size | Not applicable (review of previous studies) |
|  | C2. Age | Not applicable |
|  | C3. Sex | Not applicable |
|  | C4. Cancer type | Colorectal (CRC), Lung, Breast (BRCA), Pancreatic (PC), Glioblastoma, Hepatocellular Carcinoma (HCC). |
|  | C5. Tumor stage | Advanced. |
|  | C6. Performance status | Not applicable |
|  | C7. Relevant comorbidities | Not applicable |
|  | C8. Previous treatments | Not applicable (chemotherapy review) |
| D. Chemotherapy Protocol | D1. Chemotherapy regimen | Oxaliplatin (OXA), 5-fluorouracil (5-FU), Irinotecan (IRT), Cisplatin, Gemcitabine (GEM), Doxorubicin (DOX), Paclitaxel (PTX), Docetaxel. |
|  | D2. Treatment line | Not specified (various chemotherapy contexts). |
|  | D3. Treatment intent | Cancer treatment (chemotherapy). |
|  | D4. Number of cycles | Not specified. |
|  | D5. Combination therapies | Not specified. |
|  | D6. Dose modifications | Not specified (mentions GI toxicity can lead to dose reduction). |
| E. Microbiome Analysis | E1. Sample type | Not specified (review). |
|  | E2. Timing of collection | Pre-chemotherapy, post-chemotherapy, during. |
|  | E3. Sequencing technique | Metagenome, macrotranscriptome (via high-throughput next-generation sequencing technology). |
|  | E4. Sequencing platform | Not specified (review of multiple studies). |
|  | E5. Sequencing depth | Not specified. |
|  | E6. Bioinformatic pipeline | Not specified. |
|  | E7. Reference database | Not specified. |
|  | E8. Quality control | Not specified. |
| F. Predictive Model | F1. Model type | Not applicable (review discusses biomarkers, not a specific predictive model developed in this article). |
|  | F2. Algorithm(s) used | Not applicable. |
|  | F3. Predictive variables | Microbial taxa (e.g., *Fusobacterium nucleatum*, *Akkermansia muciniphila*, Clostridiales, Bifidobacteriaceae, Turicibacteraceae, Prevotellaceae, Firmicutes, Gammaproteobacteria, *Mycoplasma hyorhinis*), microbial metabolites (e.g., short-chain fatty acids (SCFAs), butyrate, urolithin A (UroA), ursodeoxycholic acid (UDCA), nucleosides). |
|  | F4. Feature selection method | Review of evidence from preclinical and clinical studies. |
|  | F5. Model validation | Fecal Microbiota Transplant (FMT) from wild-type donors can restore gut microbiome composition. |
|  | F6. Performance metrics | Not applicable (qualitative discussion of findings). |
|  | F7. Training set size | Not applicable. |
|  | F8. Test set size | Not applicable. |
|  | F9. Model interpretability | Cladograms and molecular pathway diagrams (Figure 1 in the article). |
|  | F10. Model availability | Not applicable. |
| G. Outcomes Assessed | G1. Primary study outcome | Chemotherapy efficacy (antitumor response). |
|  | G2. Response criteria | Not specified (review of multiple studies). |
|  | G3. Evaluation time | Not specified (review). |
|  | G4. Secondary outcomes | Toxicity (mucositis, diarrhea, peripheral neuropathy), Survival. |
|  | G5. Associated clinical/laboratory biomarkers | Fecal butyrate level, intestinal bacterial β-glucuronidase enzyme. |
|  | G6. Associated lifestyle/medication factors | Diet (high-fiber, ketogenic (KD), fasting-mimicking (FMD), high-fat Western), Probiotics, Prebiotics, Synbiotics, Antibiotics. |
|  | G7. Associated host genetic factors | Not specified. |
|  | G8. Intervention results (if applicable) | FMT from healthy donors restored gut microbiome and downregulated TLRs and MyD88 after chemotherapy. Dietary interventions (FMD, KD, high-fiber diet) can reduce toxicity and increase efficacy. Probiotics (*Lactobacillus*, *Bifidobacterium*) can reduce mucositis and neuropathy. |
| H. Key Results and Findings | H1. Key microbial taxa/species | Promoters of Chemoresistance: *Fusobacterium nucleatum* (OXA, 5-FU), Gammaproteobacteria (GEM), *Mycoplasma hyorhinis* (GEM). Efficacy Enhancers: *Akkermansia muciniphila* (DOX), Clostridiales, Bifidobacteriaceae, Turicibacteraceae, Prevotellaceae (BRCA). Toxicity Reducers: *Lactobacillus* spp. (OXA, 5-FU, IRT), *Bifidobacterium longum* (IRT). Metabolites: Butyrate (5-FU, docetaxel), Urolithin A (UroA) (5-FU, PTX, cisplatin), UDCA (5-FU), nucleosides (*Bacteroides vulgatus*). |
|  | H2. Direction of association | *F. nucleatum*, Gammaproteobacteria, *M. hyorhinis* → decrease efficacy/increase chemoresistance. *A. muciniphila*, *Lactobacillus*, *Bifidobacterium*, Clostridiales → increase efficacy/decrease toxicity. Butyrate, UroA, UDCA → increase efficacy. Nucleosides by *B. vulgatus* → increase chemoresistance. |
|  | H3. Model predictive performance | Butyrate level correlated with chemotherapy responders in CRC (likely having predictive potential). |
|  | H4. Microbial diversity findings | Chemotherapy (capecitabine + oxaliplatin) observed increased pathogenic bacteria and decreased probiotics (*Dorea*, *Streptococcus*, *Roseburia*). Reduced Firmicutes abundance. Chemotherapy-induced dysbiosis. |
|  | H5. Microbial functional/metabolic findings | Butyrate: Inhibits glucose metabolism via GPR109a-AKT, attenuates inflammation, maintains mucosal junction integrity, inhibits glioma-associated oncogene homolog 1 (GLI1). UroA: Downregulates drug transporters (MDR, ABC). UDCA: Increases *Faecalibacterium prausnitzii*, decreases *F. nucleatum*. Nucleosides: Biosynthesis by *B. vulgatus* for DNA repair, promoting chemoresistance. |
|  | H6. Preclinical/*in vitro* findings | Antibiotics abrogated antitumor effects of anti-CTLA-4. *B. fragilis* or *B. thetaiotaomicron* increased anti-CTLA-4 efficacy. *Bifidobacterium* spp. increased anti-PD-L1 efficacy. Antibiotic cocktail reduced therapeutic effect of oxaliplatin and 5-FU. |
|  | H7. Highlight of Findings: Unique Contribution | This review article comprehensively synthesizes the intricate role of the microbiome and its metabolites in modulating chemotherapy, both in efficacy and toxicity. The "major discovery" is the emergence of the concept of pharmacomicrobiomics and the demonstration that the microbiome can alter the bioavailability, bioactivity, and toxicity of chemotherapeutics. This paves the way for personalized and more effective treatment strategies, through targeted microbiome manipulation, aiming to increase efficacy and mitigate chemotherapy toxicity. |
|  | H8. Validation status (internal/external) | Not applicable (review). |
| I. Discussion/Article Implications | I1. Authors' interpretation | The gut microbiome can have "dualistic" effects on chemotherapy, increasing or decreasing its efficacy and toxicity. Understanding these effects is crucial for developing optimized therapies. |
|  | I2. Proposed biological mechanisms | The microbiome can mediate drug biotransformation, induce autophagy activation, regulate signaling pathways (TLR4/MYD88, GPR109a-AKT), modulate drug transporter expression, impact inflammation and intestinal barrier integrity, and influence nucleotide biosynthesis. |
|  | I3. Correlations with other biomarkers | Not yet fully explored. |
|  | I4. Impact of interventions (if applicable) | Diet: FMD (fasting-mimicking diet), KD (ketogenic diet), and high-fiber diets can optimize chemotherapy. Probiotics/Prebiotics/Synbiotics: Can improve efficacy and reduce toxicity, but with variable effects. Antibiotics: Targeted use can be beneficial, but indiscriminate use is harmful. FMT: Potential to mitigate toxicity and overcome chemoresistance. Engineered Bacteria/Phagotherapy: Future strategies for targeted drug delivery and combating chemoresistant bacteria. |
|  | I5. Consistency/Inconsistency of findings | Effects of probiotics can be variable. The "butyrate paradox" (opposite effects in normal vs. cancer cells) highlights the complexity. |
|  | I6. Transferability/Generalization | Microbiome alteration after chemotherapy is a generalized phenomenon in various cancer types. |
|  | I7. Causality vs. Association | The study points to a causal relationship in many cases (e.g., drug metabolism by bacteria, effects of bacterial metabolites). |
| J. Clinical Application Potential and Barriers | J1. Potential for implementation in practice | High potential for chemotherapy optimization, efficacy and toxicity prediction, and development of predictive biomarkers. |
|  | J2. Advantages | Non-invasive approach, can overcome chemoresistance and reduce side effects. |
|  | J3. Barriers to implementation | Inter-individual variability of the microbiome; difficulty in identifying a universal approach; microbiome susceptibility to endogenous and exogenous factors; need for more robust clinical trials; safety and efficacy of interventions. |
|  | J4. Cost-effectiveness | Not discussed, but the development of targeted interventions can be more cost-effective. |
|  | J5. Recommendations for future research | Validation in large clinical cohorts; longitudinal studies; investigation of the role of other microbes (archaea, fungi, viruses); analysis of spatial heterogeneity of the microbiome; evaluation of the quantitative contribution of metabolites; improvement of metabolite bioavailability. |
| K. Limitations and Bias of the Original Study | K1. Limitations declared by authors | The article does not declare specific limitations of the study itself (being a review), but points to general limitations of the field, such as the need for more robust clinical trials and the challenge of microbiome variability. |
|  | K2. Selection bias | Not applicable (review). |
|  | K3. Confounding bias | Not applicable (review). |
|  | K4. Generalization | Not applicable (review). |
|  | K5. Reproducibility | Not applicable (review). |

Table S3. Article 3: A gut microbial signature for combination immune checkpoint blockade across cancer types

| Category | Extracted Variables | Detailing |
| --- | --- | --- |
| A. Study Identification | A1. Lead Author (Year) | Gunjur A (2024) |
|  | A2. Title | A gut microbial signature for combination immune checkpoint blockade across cancer types |
|  | A3. Journal | Nature Medicine. |
|  | A4. Country/Region | Australia, USA, UK, Netherlands, Spain (in meta-analysis). |
|  | A5. Funding | Wellcome Trust (220540/Z/20/A, 206194/Z/17/Z), Cancer Research UK (C9685/A25117, C20510/A21717), Bristol Myers Squibb Ltd. Australia, Australian Commonwealth Government Medical Research Futures Fund, Tour de Cure (VicDiscovery-02-2021), Niels Stensen Fellowship. Type: Public and Private. |
|  | A6. Conflicts of interest | Yes. A.G. (speaker honorarium from Microbiotica Limited), B.M. (advisory boards for Amgen, BMS, Merck, Beigene and AstraZeneca), M.S.C. (advisory boards/consultancy for Amgen, BMS, Eisai, Ideaya, Merck, Sharp & Dohme (MSD), Nektar, Novartis, Oncosec, Pierre-Fabre, Qbio-tics, Regeneron, Roche, Merck, Moderna and Sanofi, and honoraria from BMS, MSD and Novartis), D.K. (advisory boards for BMS, MSD and Novartis), C.U. (consultancy/advisory boards for Merck Serano and AZ, lectures for IQvia and AZ; institutional research funding from Akeso Biopharma, Arcus Biosciences, Atridia, BeyondSpring Pharmaceuticals, Boehringer Ingelheim, Deciphera and Novotech), S.F. (financial support from Amgen, MSD and AZ; honoraria for advisory boards from Akesobio, Ambrax and MSD; institutional sponsorship/trials and research activities from Akesobio, Ambrax, Amgen, Axelia, AZ, Aulos, BeiGene, Cullinan, Daiichi Sankyo, Edison Oncology, Genentech, MSD, Takeda, HaiHe Biopharma, Vivace and WellMarker Bio), D.J.A. (paid consultant for Ono Therapeutics and Microbiotica Limited; receives research support from AZ, OpenTargets and BMS), T.D.L. (cofounder and scientific director of Microbiotica Limited). |
| B. Methodological Design | B1. Study type | Prospective cohort study (CA209-538 Phase II clinical trial) with meta-analysis of shotgun metagenomic data from public cohorts. |
|  | B2. Follow-up duration | Minimum of 26 months of follow-up (data cutoff: May 7, 2022). |
|  | B3. Study center(s) | Multicenter (5 hospitals in Australia). |
|  | B4. Recruitment period | October 2017 to February 2020. |
|  | B5. Ethical approval | Austin Health Human Research Ethics Committee (Melbourne, Australia) (approval: HREC/16/Austin/152). Registration: ClinicalTrials.gov (NCT02923934). |
| C. Study Population | C1. Sample size | Discovery cohort: N = 106 evaluable cancer patients for microbiome. Validation cohort: N = 364 patients (six external studies). Total meta-analysis: N = 470 (N = 383 after excluding Stable Disease (SD) patients). |
|  | C2. Age | Discovery cohort: Median 60.0 years (range 20.0–82.0). Mean (SD): 57.8 (14.4) years. |
|  | C3. Sex | Discovery cohort: Female 58.5% (62/106), Male 41.5% (44/106). |
|  | C4. Cancer type | Discovery cohort: Diverse rare cancers (upper gastrointestinal and biliary tract (UGB), neuroendocrine neoplasms (NEN), rare gynecological tumors (GYN)). Validation cohorts: Melanoma (predominantly). |
|  | C5. Tumor stage | Advanced. |
|  | C6. Performance status | Eastern Cooperative Oncology Group (ECOG) performance status of 0–1. |
|  | C7. Relevant comorbidities | Proton Pump Inhibitor (PPI) use (within 8 weeks) in 30.2% (32/106) of patients. Antibiotic use (within 8 weeks) in 7.5% (8/106). |
|  | C8. Previous treatments | Most (N=108) received previous systemic therapies (median of one line, range 0–6 lines). One patient had received prior ICI monotherapy. |
| D. Chemotherapy Protocol | D1. Chemotherapy regimen | Not applicable (focus on immunotherapy). |
|  | D2. Treatment line | Not applicable (focus on immunotherapy). |
|  | D3. Treatment intent | Immunotherapy (checkpoint inhibitors). |
|  | D4. Number of cycles | Up to 4 doses of ipilimumab + nivolumab (induction), followed by nivolumab maintenance for up to 2 years. |
|  | D5. Combination therapies | Combination immunotherapy (Cytotoxic T-Lymphocyte Associated Protein 4 (CTLA-4) and Programmed Death-1 (PD-1) inhibitors) with ipilimumab (anti-CTLA-4) and nivolumab (anti-PD-1). |
|  | D6. Dose modifications | Not specified. |
| E. Microbiome Analysis | E1. Sample type | Feces (baseline). |
|  | E2. Timing of collection | Immediately before treatment (from 7 days before to 0 day of treatment initiation). |
|  | E3. Sequencing technique | Deep shotgun metagenomics. |
|  | E4. Sequencing platform | NovaSeq 6000 S4 (2x150 bp paired-end reads). |
|  | E5. Sequencing depth | Median of 20.4 million paired-end reads per sample (discovery cohort). |
|  | E6. Bioinformatic pipeline | Genome-resolved metagenomics workflow: assembly of near-complete Metagenome Assembled Genomes (MAGs), accurate read mapping with Bowtie 2, inStrain tool for QC and quantification. Gapseq for metabolic potential inference. SHAP (SHapley Additive exPlanations) 'TreeExplainer' for feature importance. |
|  | E7. Reference database | Custom strain reference database (study MAGs supplemented with Species Reference Genomes (SRGs) from GTDB r207). GTDB-tk for taxonomic classification. VFDB (Virulence Factor Database) for virulence factors. |
|  | E8. Quality control | Human DNA decontamination (bwa, BMTagger), read trimming, removal of reads mapped with <0.5 genome coverage. Filtering of MAGs with ≥90% completeness and ≤5% contamination (CheckM2, GUNC). Decontam for contaminants. |
| F. Predictive Model | F1. Model type | Machine Learning (ML) classifier. |
|  | F2. Algorithm(s) used | Random Forest (RF) (for prediction), SHAP 'TreeExplainer' (for interpretability/feature importance). |
|  | F3. Predictive variables | Microbial taxa (strain, species, genus, family abundances transformed by Centered Log Ratio (CLR)), clinical factors (15 relevant variables: age, sex, BMI (Body Mass Index), ECOG PS, histology cohort, tumor size, study site, fecal collection season, antibiotic/PPI/chemotherapy use, NLR, platelets, albumin, LDH). |
|  | F4. Feature selection method | No formal feature selection method specified for RF. Feature importance was assessed by the SHAP algorithm. |
|  | F5. Model validation | Five-fold cross-validation repeated 20 times (100 models). Leave-one-group-out cross-validation for generalizability across cancer types. External validation with meta-analysis of six comparable studies (N=364). |
|  | F6. Performance metrics | Receiver Operating Characteristic Area Under the Curve (ROC AUC) (for RvsP (Objective Response vs. Progression) and PFS12 (Progression-Free Survival at 12 months)), Kendall τ, Mann–Whitney U test. |
|  | F7. Training set size | Discovery cohort: 77 evaluable patients (RvsP), 106 patients (PFS12). External validation cohorts. |
|  | F8. Test set size | Discovery cohort: 77 evaluable patients (RvsP), 106 patients (PFS12). External validation cohorts. |
|  | F9. Model interpretability | SHAP 'TreeExplainer' to identify the most important strains. Phylogenetic visualizations and metabolic pathway heatmap for the "top 22" strains. |
|  | F10. Model availability | Code to replicate analyses is available on GitHub (https://github.com/agunjur/cancer_microbiome_CICB/). |
| G. Outcomes Assessed | G1. Primary study outcome | Objective Response vs. Progression (RvsP) (defined as Complete Response (CR) or Partial Response (PR) vs. Disease Progression (PD) or clinical PD (cPD)). |
|  | G2. Response criteria | RECIST 1.1 (Response Evaluation Criteria in Solid Tumors) (best overall response - BOR). |
|  | G3. Evaluation time | PFS at 12 months (PFS12), Overall Survival (OS) at 12 months. |
|  | G4. Secondary outcomes | PFS12, OS12, alpha microbiome diversity, beta microbiome diversity, microbiome composition. |
|  | G5. Associated clinical/laboratory biomarkers | Serum albumin (positive association with BOR, P = 0.0056), Neutrophil-to-Lymphocyte Ratio (NLR) (negative association with BOR, P = 0.0033), age, sex, BMI, LDH (lactate dehydrogenase), platelets, tumor stage (T, N), measurable tumor extent. |
|  | G6. Associated lifestyle/medication factors | PPI use (association with BOR: P = 0.017), antibiotic use, fecal collection season. |
|  | G7. Associated host genetic factors | Not specified. |
| H. Key Results and Findings | H1. Key microbial taxa/species | 22 most important strains: *Faecalibacterium sp900539885* (uncultured strain), *Faecalibacterium prausnitzii D* (two strains), *Faecalibacterium prausnitzii G*, *Gemmiger formicilis*. Culprits (negative association): *Bifidobacterium dentium*, *Akkermansia muciniphila B*, *Spyradocola merdavium*, *Ruthenibacterium lactatiformans*, *Avimicrobium caecorum*, *Lachnospira sp000437735* (two strains), *Copromonas sp000435795*, *Clostridium Q fessum*, *Mediterraneibacter faecis*, *CAG-317 sp000433535*, *CAG-83 sp900552475*, *Oliverpabstia intestinalis*, *ER4 sp000765235*, *Lawsonibacter sp900066825*. Friends (positive association): *Bifidobacterium longum* (strain). |
|  | H2. Direction of association | Culprits (negative association with response): *Bifidobacterium dentium*, *Akkermansia muciniphila B*, *Ruthenibacterium lactatiformans*, *Avimicrobium caecorum*, and other strains of Lachnospiraceae, Oscillospiraceae, and Ruminococcaceae. Friends (positive association with response): *Faecalibacterium* strains, *Bifidobacterium longum*, *Gemmiger formicilis*. |
|  | H3. Model predictive performance | RvsP (strain): AUC = 0.73. PFS12 (strain): AUC = 0.70. Cross-validation across histologies: AUC = 0.75 (overall). External validation in CICB cohorts: median AUC = 0.65. External validation in anti-PD-1 cohorts: median AUC = 0.51. |
|  | H4. Microbial diversity findings | Positive monotonic association between BOR and Shannon diversity index (τ = 0.22, P = 0.003). Gross compositional differences by BOR group (PERMANOVA P = 0.0319). |
|  | H5. Microbial functional/metabolic findings | Butyrate pathways (acetyl-CoA) complete in all "positive" Ruminococcaceae strains (5/5), but none of the "negative" ones (0/2). |
|  | H6. Preclinical/*in vitro* findings | Not applicable (clinical study with meta-analysis of shotgun metagenomic data). |
|  | H7. Highlight of Findings: Unique Contribution | This study reveals that strain-level resolution of the gut microbiome is crucial for enhancing the predictive capacity of response to combination immunotherapy (CICB), outperforming clinical markers and higher taxonomic resolutions. The "major discovery" is the identification that microbial signatures are specific to the ICI regimen, with models trained on CICB not generalizing to anti-PD-1 monotherapy. This provides a fundamental "roadmap" for the development of microbiome-targeted biomarkers and therapies, indicating that personalization should consider the type of ICI regimen and not just the cancer type. |
|  | H8. Validation status (internal/external) | Internal validation: Cross-validation (20x repeated 5-fold) and leave-one-group-out (discovery cohort). External validation: Meta-analysis with six comparable studies. |
| I. Discussion/Article Implications | I1. Authors' interpretation | Accurate strain-level microbial quantification is crucial for predicting CICB response. Signatures are robust across diverse cancer types and geographies but are specific to the ICI regimen (CICB vs. anti-PD-1 monotherapy). |
|  | I2. Proposed biological mechanisms | Positive association of *Faecalibacterium* strains with butyrate (implicated in cytotoxic T cell activation). *Bifidobacterium longum* linked to positive outcomes, while *B. dentium* to negative outcomes. |
|  | I3. Correlations with other biomarkers | Clinical factors (albumin, NLR) are more useful for delineating patients with worse prognosis than for predicting responders. |
|  | I4. Impact of interventions (if applicable) | Does not discuss interventions. |
|  | I5. Consistency/Inconsistency of findings | Low reproducibility of species-level microbial biomarkers in previous meta-analyses can be overcome with strain-level resolution and distinction between ICI regimens. |
|  | I6. Transferability/Generalization | Strain-level signatures are generalizable across diverse cancer types and geographical locations but are specific to the ICI regimen. |
|  | I7. Causality vs. Association | The study identifies predictive associations but does not establish direct causality. Functional validation is needed to confirm mechanisms. |
| J. Clinical Application Potential and Barriers | J1. Potential for implementation in practice | High potential for microbiome diagnostic biomarkers or therapeutic adjuncts tailored to the ICI regimen, potentially guiding patient management. |
|  | J2. Advantages | Improved predictive performance compared to clinical data and higher-level taxonomic approaches. Tumor type agnosticism. |
|  | J3. Barriers to implementation | Strong heterogeneity in microbiome composition between cohorts due to collection and DNA extraction methods. Sample size still limited for definitive clinical robustness. |
|  | J4. Cost-effectiveness | Not discussed, but deep metagenomic analysis can be more expensive than 16S rRNA. |
|  | J5. Recommendations for future research | Validation in larger and more diverse cohorts. Standardization of collection and extraction methods. Cultivation of strains for *in vitro*/*in vivo* functional testing. |
| K. Limitations and Bias of the Original Study | K1. Limitations declared by authors | Limited size of the discovery cohort and meta-analysis. Possible errors in the MAGs (nc-MAGs) used. Difficulty in correcting for DNA extraction bias without impacting geographical biological variance. Lack of strain cultivation for mechanistic insights. |
|  | K2. Selection bias | Not explicitly stated, but the discovery cohort includes patients with rare cancers, which may affect representativeness. |
|  | K3. Confounding bias | Variability between cohorts due to geographical differences and DNA collection/extraction methods. |
|  | K4. Generalization | The results for CICB are promising across various cancer types, but the lack of generalization to anti-PD-1 monotherapy is a limitation for universal application. |
|  | K5. Reproducibility | Data and code available in relevant repositories (European Nucleotide Archive, Zenodo, GitHub). |

Table S4. Article 4: Crosstalk between the gut microbiome and clinical response in locally advanced thoracic esophageal squamous cell carcinoma during neoadjuvant camrelizumab and chemotherapy

| Category | Extracted Variables | Detailing |
| --- | --- | --- |
| A. Study Identification | A1. Lead Author (Year) | Xu L (2022) |
|  | A2. Title | Crosstalk between the gut microbiome and clinical response in locally advanced thoracic esophageal squamous cell carcinoma during neoadjuvant camrelizumab and chemotherapy |
|  | A3. Journal | Annals of Translational Medicine. |
|  | A4. Country/Region | China |
|  | A5. Funding | Medical and Health Technology Plan of Zhejiang Province (2019KY330 and 2020KY057), Key Laboratory of Prevention, Diagnosis and Therapy of Upper Gastrointestinal Cancer of Zhejiang Province (2022E10021). Type: Public. |
|  | A6. Conflicts of interest | No conflicts of interest declared by the authors. |
| B. Methodological Design | B1. Study type | Prospective cohort study. |
|  | B2. Follow-up duration | Not specified in the article (follow-up cutoff date). |
|  | B3. Study center(s) | Unicenter (The Cancer Hospital of the University of Chinese Academy of Sciences (Zhejiang Cancer Hospital)). |
|  | B4. Recruitment period | August 11, 2020 to February 8, 2021. |
|  | B5. Ethical approval | Zhejiang Cancer Hospital Ethics Committee (IRB-2020-320). Registration: ClinicalTrials.gov (NCT04506138). |
| C. Study Population | C1. Sample size | 46 patients initially recruited; 37 patients evaluable for pathological Complete Response (pCR) or Major Pathological Response (MPR). Total fecal samples: 44 (pre-treatment), 42 (pre-surgery), 35 (post-surgery). |
|  | C2. Age | Mean: 63.3 ± 5.96 years (age range: 18–75 years in inclusion criteria). |
|  | C3. Sex | Male: 95.7% (44/46). |
|  | C4. Cancer type | Thoracic Esophageal Squamous Cell Carcinoma (ESCC). |
|  | C5. Tumor stage | Locally advanced (T2–4aNanyM0 or T1N1–3M0, according to American Joint Committee on Cancer (AJCC) TNM 8th edition). Clinical stage: cII (27.0%), cIII–cIVA (73.0%). |
|  | C6. Performance status | Eastern Cooperative Oncology Group (ECOG) performance status of 0 or 1. |
|  | C7. Relevant comorbidities | Excluded patients with autoimmune diseases, interstitial lung diseases. |
|  | C8. Previous treatments | Treatment-naive (had not received previous treatment). |
| D. Chemotherapy Protocol | D1. Chemotherapy regimen | Camrelizumab (Programmed Cell Death Protein 1 (PD-1) inhibitor), carboplatin, and albumin-bound paclitaxel. |
|  | D2. Treatment line | Neoadjuvant. |
|  | D3. Treatment intent | Neoadjuvant. |
|  | D4. Number of cycles | Two cycles of camrelizumab + chemotherapy before surgery. |
|  | D5. Combination therapies | Immunotherapy (camrelizumab) + chemotherapy (carboplatin and albumin-bound paclitaxel). |
|  | D6. Dose modifications | Not specified. |
| E. Microbiome Analysis | E1. Sample type | Feces. |
|  | E2. Timing of collection | 1. Baseline (within 3 days before neoadjuvant treatment). 2. During treatment (3 days before surgery). 3. Post-treatment (at first postoperative defecation). |
|  | E3. Sequencing technique | 16S rRNA sequencing (V3–V4 regions). |
|  | E4. Sequencing platform | Illumina NovaSeq 6000. |
|  | E5. Sequencing depth | Total of 676,674 valid sequences, 7,121 Amplicon Sequence Variants (ASVs). |
|  | E6. Bioinformatic pipeline | QIIME2, R packages (v3.2.0), MEGAN, GraPhlAn. |
|  | E7. Reference database | Not specified. |
|  | E8. Quality control | Sequence processing with QIIME2, filtering, and ASV clustering. |
| F. Predictive Model | F1. Model type | Microbial signature for prediction of pathological response and Adverse Events (AEs). |
|  | F2. Algorithm(s) used | Linear Discriminant Analysis Effect Size (LEfSe) to detect differentially abundant taxa. |
|  | F3. Predictive variables | Microbial taxa at phylum, class, order, family, genus level. |
|  | F4. Feature selection method | LEfSe (LDA score > 2.0). |
|  | F5. Model validation | Not specified. |
|  | F6. Performance metrics | Not directly specified (LEfSe score and P-value). |
|  | F7. Training set size | Not specified. |
|  | F8. Test set size | Not specified. |
|  | F9. Model interpretability | Cladograms and bar plots with LDA scores to visualize enriched taxa in different groups. |
|  | F10. Model availability | Not specified. |
| G. Outcomes Assessed | G1. Primary study outcome | Pathological Complete Response (pCR) and Major Pathological Response (MPR). |
|  | G2. Response criteria | pCR (0% residual tumor), MPR (≤10% residual tumor), RECIST 1.1 for initial evaluation. |
|  | G3. Evaluation time | Pathological evaluation after surgery. |
|  | G4. Secondary outcomes | Adverse Events (AEs) Grade ≥3, Microsatellite Stability (MSI), Tumor Mutational Burden (TMB). |
|  | G5. Associated clinical/laboratory biomarkers | Programmed Cell Death Ligand 1 (PD-L1) (TPS - Tumor Proportion Score, CPS - Combined Positive Score), TMB (muts/Mb). |
|  | G6. Associated lifestyle/medication factors | Alcohol consumption, smoking. |
|  | G7. Associated host genetic factors | PD-L1 (TPS and CPS), TMB. |
| H. Key Results and Findings | H1. Key microbial taxa/species | Non-pCR enriched: Proteobacteria (LDA = 4.16, P = 0.009), *Dialister* (LDA = 3.28, P = 0.032), Aeromonadales, Pseudomonadales, Thermi, Deinococci, Moraxellaceae, Rhodocyclales, Rhodocyclaceae, *Acinetobacter*. Non-MPR enriched: Pseudomonadales (LDA = 2.58, P = 0.035), mitochondrial family. MPR enriched: Barnesiellaceae (LDA = 3.33, P = 0.003), *Pyramidobacter* (LDA = 3.14, P = 0.002), Dethiosulfovibrionaceae, Odoribacteraceae, *Butyricimonas*, *Prevotella*, *Barnesiella*, *Odoribacter*. AEs Grade ≥3 enriched: *Succiniclasticum*, *Nakamurella*, *Rhizobium*, *Granulicella*, Phyllobacteriaceae, Pelagibacteraceae, Actinosynnemataceae, *Aquirestis*, *Flavisolibacter*, *Chelativorans*, Coxiellaceae, *Acidicapsa*, Acidobacteriaceae, *Lentzea*, *Staphylococcus*, *Plesiomonas*, *Dysgonomonas*, *Pseudonocardia*, *Ellin6075*. AEs Grade 1-2 enriched: *Phascolarctobacterium* (LDA = 3.48, P = 0.001), Odoribacteraceae, Synergistia (order, class, phylum), *Butyricimonas*, Deltaproteobacteria, Synergistes, *Odoribacter*, *Anaerotruncus*. |
|  | H2. Direction of association | Alpha diversity (Chao1, Shannon, Good’s coverage) gradually decreased after neoadjuvant treatment and surgery (P < 0.05). Specific taxa enriched in non-responders (pCR/MPR) or in patients with more severe AEs. Specific taxa enriched in responders (MPR) or in patients with less severe AEs. |
|  | H3. Model predictive performance | No AUC or other predictive performance metrics specified beyond LDA scores. |
|  | H4. Microbial diversity findings | Alpha diversity (Chao1, Shannon, Good’s coverage) significantly decreased between the three time points (P < 0.05). No significant differences in alpha diversity between response groups (MPR or pCR), AE grade, TPS, CPS, and TMB. |
|  | H5. Microbial functional/metabolic findings | Not directly assessed, but taxa enriched in MPR (*Butyricimonas*) are butyrate producers. *Prevotella* related to high CPS. |
|  | H6. Preclinical/*in vitro* findings | Not applicable. |
|  | H7. Highlight of Findings: Unique Contribution | This study highlights the dynamics of the gut microbiome during neoadjuvant immunotherapy combined with chemotherapy and surgery for ESCC. The "major discovery" is the identification of specific microbial signatures that predict pathological response and the risk of severe adverse events (AEs) even before treatment. This suggests that the baseline intestinal microbiota profile can serve as a crucial biomarker to personalize treatment strategies and anticipate toxicities in a complex combined therapy setting for ESCC. |
|  | H8. Validation status (internal/external) | Not specified. |
| I. Discussion/Article Implications | I1. Authors' interpretation | The gut microbiome is related to pathological response and AEs in combined neoadjuvant therapy for ESCC. There is variation in microbiome diversity and composition during treatment. |
|  | I2. Proposed biological mechanisms | Gut microflora can regulate cancer development through inflammatory reaction, immune reaction, specific protein activation, and production of carcinogenic metabolites. Microbes influence immune cells (dendritic cells (DCs), Natural Killer (NK) cells, T cell subsets) in the tumor microenvironment. Taxa enriched in MPR (e.g., *Prevotella*) are related to high CPS. |
|  | I3. Correlations with other biomarkers | *Phascolarctobacterium* enriched in patients with PD-L1 CPS ≥10 and TPS ≥1%. *Ruminococcus* negatively associated with clinical stage. |
|  | I4. Impact of interventions (if applicable) | Does not discuss interventions. |
|  | I5. Consistency/Inconsistency of findings | Inconsistent results among studies due to different trial designs (cancer types, clinical stage, treatment regimens, outcomes). |
|  | I6. Transferability/Generalization | Not explicitly discussed, but findings are specific to ESCC patients. |
|  | I7. Causality vs. Association | The study identifies associations and correlations. |
| J. Clinical Application Potential and Barriers | J1. Potential for implementation in practice | High potential for the development of diagnostic fecal tests to identify gut dysbiosis, predicting efficacy or AEs in locally advanced ESCC patients treated with neoadjuvant anti-PD-1 immunotherapy and chemotherapy. |
|  | J2. Advantages | Non-invasive approach, easy to collect, potential to guide personalized treatment. |
|  | J3. Barriers to implementation | No consensus on biomarkers yet. Inconsistency among studies. Need for more research on the role of each bacterial species. |
|  | J4. Cost-effectiveness | Not discussed. |
|  | J5. Recommendations for future research | Investigate correlation with dietary history. Use metagenomic sequencing for species-level resolution. Increase sample collection frequency and include adjuvant immunotherapy. |
| K. Limitations and Bias of the Original Study | K1. Limitations declared by authors | Did not identify correlation with dietary history. Use of 16S rRNA gene sequence (not metagenomics) limits species-level resolution. Few sample collection points (especially post-surgery). Lack of long-term survival data. |
|  | K2. Selection bias | Predominance of male patients (95.7%). |
|  | K3. Confounding bias | The impact of lifestyle factors like diet was not addressed. |
|  | K4. Generalization | Predominantly Chinese population with ESCC, may not generalize to other ethnicities or esophageal cancer types. |
|  | K5. Reproducibility | Not discussed. |

Table S5. Article 5: Role of Gut Microbiome in Neoadjuvant Chemotherapy Response in Urothelial Carcinoma: A Multi-institutional Prospective Cohort Evaluation

| Category | Extracted Variables | Detailing |
| --- | --- | --- |
| A. Study Identification | A1. Lead Author (Year) | Bukavina L (2024) |
|  | A2. Title | Role of Gut Microbiome in Neoadjuvant Chemotherapy Response in Urothelial Carcinoma: A Multi-institutional Prospective Cohort Evaluation |
|  | A3. Journal | Cancer Research Communications. |
|  | A4. Country/Region | USA (Philadelphia, Cleveland) |
|  | A5. Funding | P30CA043703 Case Western Comprehensive Cancer Center Microbiome Grant, P30 CA006927 Fox Chase Cancer Center Support Grant, NIH grant no. R01AI145289-01A1, CA181178 Department of Defense CDMRP. Type: Public. |
|  | A6. Conflicts of interest | Yes. L. Bukavina (associate editor, editorial board member), D.M. Geynisman (honoraria from Exelixis, Merck, AstraZeneca, Bristol Myers Squibb), J.R. Brown (honoraria from Pfizer/Seagen, EMD-Serono, AstraZeneca), E.R. Plimack (honoraria/grants from AbbVie, Astellas, AstraZeneca, BMS, Eisai, EMD Serono, Exelixis, IMV, Merck, Pfizer, Regeneron, Seagen, Seattle Genetics, Signatera, Synthekine), P.H. Abbosh (Janssen and Natera grants, pending patent). |
| B. Methodological Design | B1. Study type | Prospective cohort study (in humans) and *in vivo* study (murine model). |
|  | B2. Follow-up duration | Not specified (sample collection until March 2023). |
|  | B3. Study center(s) | Multicenter (two in USA: Case Western Reserve/University Hospitals Cleveland Medical Center, Fox Chase Cancer Center). |
|  | B4. Recruitment period | July 2018 to March 2023. |
|  | B5. Ethical approval | Institutional Review Board (IRB) of Case Western Reserve/University Hospitals Cleveland Medical Center (IRB# STUDY20200350), Fox Chase Cancer Center (IRB #18-4001). Animal study approved by FCCC Institutional Animal Care and Use Committee (IACUC 19-03). |
| C. Study Population | C1. Sample size | Humans: 142 urothelial carcinoma (UC) patients, 48 controls (partners). Total for analysis: 190 patients. Murine Model: 23 mice exposed to N-butyl-N-(4-hydroxybutyl)-nitrosamine (BBN), 18 controls (water). |
|  | C2. Age | Humans: Not specified (mean/median). Murine Model: 8–10 weeks of age at BBN exposure initiation. |
|  | C3. Sex | Humans: Not specified (distribution). Murine Model: Females and males (14 males, 9 females exposed to BBN; 10 males, 8 females control). |
|  | C4. Cancer type | Humans: Muscle-invasive bladder urothelial carcinoma (MIBC). Murine Model: BBN-induced tumors (bladder carcinogen). |
|  | C5. Tumor stage | Humans: MIBC. Murine Model: BBN-induced tumors. |
|  | C6. Performance status | Humans: Not specified. Murine Model: Not applicable. |
|  | C7. Relevant comorbidities | Humans: Excluded patients with antibiotics in the 6 weeks prior to collection, history of *Clostridioides difficile*. Murine Model: Not applicable. |
|  | C8. Previous treatments | Humans: Treatment with neoadjuvant chemotherapy (NAC). Murine Model: Not applicable. |
| D. Chemotherapy Protocol | D1. Chemotherapy regimen | Neoadjuvant Chemotherapy (NAC): Methotrexate, Vinblastine, Doxorubicin, Cisplatin (dd-MVAC) or Gemcitabine and Cisplatin (GC). |
|  | D2. Treatment line | Neoadjuvant. |
|  | D3. Treatment intent | Neoadjuvant (pre-cystectomy). |
|  | D4. Number of cycles | NAC: before cystectomy, after cycle 2 (of 3). |
|  | D5. Combination therapies | Not specified. |
|  | D6. Dose modifications | Not specified. |
| E. Microbiome Analysis | E1. Sample type | Humans: Feces (rectal swabs, OMNIgene GUT kits). Murine Model: Fecal pellets. |
|  | E2. Timing of collection | Humans: Before NAC, after NAC cycle 2, before cystectomy. Controls (partners) on the same dates. Murine Model: Pre-treatment, 6 weeks, 12 weeks, between 16-22 weeks (after tumor appearance). |
|  | E3. Sequencing technique | 16S rRNA sequencing (V4 region). |
|  | E4. Sequencing platform | Not specified. |
|  | E5. Sequencing depth | Not specified. |
|  | E6. Bioinformatic pipeline | QIIME 2, LEfSe (Linear Discriminant Analysis Effect Size), MaAsLin2 (Microbiome Multivariable with Linear Models). |
|  | E7. Reference database | SILVA 16S ribosomal RNA sequence database (release 138.1). |
|  | E8. Quality control | Sequences with >97% identity clustered into Operational Taxonomic Units (OTUs). OTUs filtered with less than 0.001% abundance. |
| F. Predictive Model | F1. Model type | Machine Learning classifier for complete response (CR) prediction. |
|  | F2. Algorithm(s) used | Random Forest. |
|  | F3. Predictive variables | Microbial taxa (OTUs at genus level). |
|  | F4. Feature selection method | Not specified. |
|  | F5. Model validation | Five rounds of 1,000-fold cross-validation. |
|  | F6. Performance metrics | Area Under the Curve (AUC) = 0.88 (CR), Sensitivity = 0.70, Specificity = 0.80, Positive Predictive Value (PPV) = 0.88, Negative Predictive Value (NPV) = 0.571. |
|  | F7. Training set size | Not specified (referring to cross-validation). |
|  | F8. Test set size | Not specified (referring to cross-validation). |
|  | F9. Model interpretability | Feature importance: *Oscillibacter* (0.051), *Fusicatenibacter* (0.037), *Bacteroides* (0.032). |
|  | F10. Model availability | Not specified. |
| G. Outcomes Assessed | G1. Primary study outcome | Response to NAC: pathological Complete Response (CR) (pT0N0M0) or Partial Response (PR) (pTis, Ta, T1N0M0). |
|  | G2. Response criteria | Pathological (pT0N0M0 for CR, pTis, Ta, T1N0M0 for PR). |
|  | G3. Evaluation time | At cystectomy. |
|  | G4. Secondary outcomes | Short-Chain Fatty Acid (SCFA) levels in blood and feces, impact of demographic factors on microbiome, GM changes during carcinogenesis (murine model). |
|  | G5. Associated clinical/laboratory biomarkers | Not specified. |
|  | G6. Associated lifestyle/medication factors | Smoking, study cohort, BBN use (murine model). |
|  | G7. Associated host genetic factors | Not specified. |
| H. Key Results and Findings | H1. Key microbial taxa/species | In UC vs. Controls: Higher *Prevotella* (P = 0.001) and *Porphyromonas* (P = 0.001) in UC; lower *Faecalibacterium* (P = 0.04) in UC. In non-responders (NR) to NAC: Higher *Bacteroides* (26.95% vs 18.93%, P < 0.01) and *Pseudomonas* (2.18% vs 1.67%, P = 0.03). In responders (CR) to NAC: Higher abundance of Lachnospiraceae (LDA > 3, P < 0.01). In smokers: *Anaerostipes*, *Eggerthella*, *Fusicatenibacter*. In BBN-exposed (murine): Higher *Bacteroides*. |
|  | H2. Direction of association | *Prevotella* and *Porphyromonas* enriched in UC. *Faecalibacterium* reduced in UC. *Bacteroides* and *Pseudomonas* enriched in NR. Lachnospiraceae enriched in CR. *Desulfovibrio* reduced with pT0. *Bacteroides* increased in BBN-exposed mice. |
|  | H3. Model predictive performance | Random Forest model for CR: AUC = 0.88 (95% CI: 0.81–0.94). Clinical variables: AUC = 0.50. |
|  | H4. Microbial diversity findings | Higher alpha diversity in UC patients (Shannon P = 0.00029, Simpson P = 0.0029, Chao P = 0.001). No significant difference in alpha or beta diversity between CR and NR. |
|  | H5. Microbial functional/metabolic findings | SCFA: No significant difference in concentrations between CR and NR. Fecal isobutyric acid positively correlated with *Akkermansia* (rs = 0.51, P = 0.017) and Clostridia (rs = 0.52, P = 0.018). Fecal propionic acid positively correlated with *Lactobacillus* (rs = 0.49, P = 0.02) and Enterobacteriaceae (rs = 0.52, P < 0.03). |
|  | H6. Preclinical/*in vitro* findings | Murine model (BBN): *Bacteroides* abundance increases during BBN exposure and normalizes after carcinogen withdrawal. No significant differences in alpha or beta diversity by sex. |
|  | H7. Highlight of Findings: Unique Contribution | This study offers the first multi-institutional and large-scale evaluation of gut microbiome composition in UC patients undergoing NAC. The "major discovery" is the identification that elevated levels of *Bacteroides* are associated with a worse response to NAC, and that a Machine Learning model based on the microbiome outperforms traditional clinical variables in predicting pathological complete response (CR). This establishes a robust foundation for the development of complex microbial signatures as predictive biomarkers for optimizing therapies in UC. |
|  | H8. Validation status (internal/external) | Internal validation (cross-validation). |
| I. Discussion/Article Implications | I1. Authors' interpretation | The gut microbiome is a crucial and modifiable aspect of anticancer therapeutic response in UC. The presence of *Bacteroides* is linked to poor NAC response, corroborating findings in other cancers (melanoma, pancreas, colon). |
|  | I2. Proposed biological mechanisms | *Bacteroides* and *Prevotella* frequently associated with tumorigenesis. Increased *Bacteroides* during NAC may have implications for response. |
|  | I3. Correlations with other biomarkers | Factors such as sex, smoking, age, and stage correlate with specific bacterial profiles. |
|  | I4. Impact of interventions (if applicable) | Not discussed. |
|  | I5. Consistency/Inconsistency of findings | Findings of increased *Prevotella* and *Bacteroides* in UC patients align with previous research. Observations in the murine model regarding *Bacteroides* corroborate. |
|  | I6. Transferability/Generalization | The inclusion of partners as controls helps mitigate environmental influences, but different cohorts and chemotherapy regimens may limit generalization. |
|  | I7. Causality vs. Association | The study identifies associations. Functional validation is necessary to determine causality. |
| J. Clinical Application Potential and Barriers | J1. Potential for implementation in practice | Identification of patients likely not to benefit from NAC, who could be directed to alternative treatments or early surgery. |
|  | J2. Advantages | Predictive biomarker superior to traditional clinical variables. Non-invasive and promising strategy. |
|  | J3. Barriers to implementation | Need for validation in larger and more detailed studies. Need to integrate microbial analysis with tumor, genomic, and circulating tumor DNA data. |
|  | J4. Cost-effectiveness | Not discussed. |
|  | J5. Recommendations for future research | Larger studies for validation. Clinical trials integrating GM modulation. Manipulation of *Bacteroides* in murine models for therapeutic developments. |
| K. Limitations and Bias of the Original Study | K1. Limitations declared by authors | No concomitant tumor data (genomic mutations, immune infiltration) were collected. The impact of nutritional data or over-the-counter (OTC) medications was not evaluated. Different NAC regimens (GC and MVAC) may affect the results. |
|  | K2. Selection bias | Not explicitly stated. |
|  | K3. Confounding bias | The impact of nutritional data or OTC medications was not addressed. Different chemotherapy regimens. |
|  | K4. Generalization | The population is from two American institutions, which may limit generalization. |
|  | K5. Reproducibility | Raw data can be provided upon request; analyzed data are in the Supplementary Appendix. |

Table S6. Article 6: A Phase II Randomized Clinical Trial and Mechanistic Studies Using Improved Probiotics to Prevent Oral Mucositis Induced by Concurrent Radiotherapy and Chemotherapy in Nasopharyngeal Carcinoma

| Category | Extracted Variables | Detailing |
| --- | --- | --- |
| A. Study Identification | A1. Lead Author (Year) | Xia C (2021) |
|  | A2. Title | A Phase II Randomized Clinical Trial and Mechanistic Studies Using Improved Probiotics to Prevent Oral Mucositis Induced by Concurrent Radiotherapy and Chemotherapy in Nasopharyngeal Carcinoma |
|  | A3. Journal | Frontiers in Immunology. |
|  | A4. Country/Region | China (Nanchang, Guangzhou) |
|  | A5. Funding | National Natural Science Foundation of China (82060638), Academic and technical leaders of major disciplines in Jiangxi Province (20194BCJ22032), Double thousand plan of Jiangxi Province (high end Talents Project of scientific and technological innovation). Type: Public. |
|  | A6. Conflicts of interest | No conflicts of interest declared by the authors. |
| B. Methodological Design | B1. Study type | Randomized, double-blind, placebo-controlled Phase II clinical trial (in humans), and mechanistic studies in an animal model (Sprague-Dawley rats). |
|  | B2. Follow-up duration | 7 weeks of probiotic/placebo treatment. OM (Oral Mucositis) evaluation on days 7, 14, and 21 in animal model. |
|  | B3. Study center(s) | Unicenter (Jiangxi Cancer Hospital in China for the clinical trial). |
|  | B4. Recruitment period | Not specified (mentions June 2016 for bacterial screening). |
|  | B5. Ethical approval | Local Clinical Research Ethics Committee (Clinical Trials number, NCT03112837). Nanchang University Ethics Committee for animal studies. |
| C. Study Population | C1. Sample size | Humans: 85 patients screened, 77 randomized, 70 finally included (34 placebo, 36 probiotic). Animal Model: 39 rats (Control:13, Model:13, Treatment:13). |
|  | C2. Age | Humans: 18–70 years. Animal Model: 8–10 weeks of age. |
|  | C3. Sex | Humans: Exact distribution not specified, but no significant difference between groups. Animal Model: Males. |
|  | C4. Cancer type | Humans: Nasopharyngeal Carcinoma (NPC). Animal Model: Not specified, but oral mucositis induced. |
|  | C5. Tumor stage | Humans: Locally advanced NPC. Animal Model: Not applicable. |
|  | C6. Performance status | Humans: Karnofsky score (no mean/distribution details). Animal Model: Not applicable. |
|  | C7. Relevant comorbidities | Excluded patients with previous cancer, inability to take oral medication, high risk of antimicrobial agents, recurrent OM, severe/uncontrolled infections. |
|  | C8. Previous treatments | Humans: Initial treatment (had not received previous chemo/radiotherapy). Animal Model: Not applicable. |
| D. Chemotherapy Protocol | D1. Chemotherapy regimen | Humans: Cisplatin (100 mg/m2 on days 1, 22, and 43). Animal Model: Busulfan (6 mg/kg for 4 days). |
|  | D2. Treatment line | Human: Not specified, but concurrent with radiotherapy (concurrent chemoradiotherapy (CCRT)). Animal Model: Not applicable. |
|  | D3. Treatment intent | CCRT for NPC. |
|  | D4. Number of cycles | Human: 3 cycles of cisplatin. Animal Model: Not applicable (4-day duration for busulfan). |
|  | D5. Combination therapies | Humans: Radiotherapy (32 fractions of 70 Gy, 2.19 Gy/d, 5 d/week) + Chemotherapy (cisplatin). Animal Model: Irradiation (20 Gy in head region) + Chemotherapy (busulfan). |
|  | D6. Dose modifications | Not specified. |
| E. Microbiome Analysis | E1. Sample type | Humans: Feces. Animal Model: Feces, tongue, and colon tissues. |
|  | E2. Timing of collection | Humans: Before treatment (BRCP - before radiotherapy, chemotherapy, and placebo treatment / BRCPM - before radiotherapy, chemotherapy, and probiotic combination treatment), after treatment (ARCP - after radiotherapy, chemotherapy, and placebo treatment / ARCPM - after radiotherapy, chemotherapy, and probiotic combination treatment). Animal Model: Before sacrifice (days 7, 14, 21). |
|  | E3. Sequencing technique | 16S rRNA sequencing (V3-V4 regions). |
|  | E4. Sequencing platform | Not specified. |
|  | E5. Sequencing depth | Humans: 2,936,897 clean tags, 9,941 OTUs (Operational Taxonomic Units). Animal Model: Not specified. |
|  | E6. Bioinformatic pipeline | FLASH Software, UPARSE, RDP classifier, QIIME (for alpha-diversity), NMDS (Non-metric Multidimensional Scaling), PCoA (Principal Coordinate Analysis). |
|  | E7. Reference database | RDP (Ribosomal Database Project) classifier. |
|  | E8. Quality control | Quality filtering with UPARSE, clustering into OTUs with 97% identity. |
| F. Predictive Model | F1. Model type | Microbial signature for OM prediction and immune response modulation. |
|  | F2. Algorithm(s) used | Not applicable directly (focus on diversity and differential abundance analyses). |
|  | F3. Predictive variables | Microbial taxa (*Lactobacillus*, *Bifidobacterium*, *Akkermansia*, *Clostridium*, *Enterococcus*, *Enterobacter*, Firmicutes, Bacteroidetes, Proteobacteria, Actinobacteria, Lachnospiraceae, *Ruminococcus*). |
|  | F4. Feature selection method | Not applicable directly (focus on diversity and differential abundance analyses). |
|  | F5. Model validation | Not applicable (focus on clinical trial and animal model). |
|  | F6. Performance metrics | Not applicable. |
|  | F7. Training set size | Not applicable. |
|  | F8. Test set size | Not applicable. |
|  | F9. Model interpretability | NMDS and PCoA to visualize microbial community clustering. |
|  | F10. Model availability | Not applicable. |
| G. Outcomes Assessed | G1. Primary study outcome | Incidence of severe oral mucositis (OM) (grade 3 or higher) in NPC patients. |
|  | G2. Response criteria | National Cancer Institute Common Terminology Criteria for Adverse Events (CTCAE) version 4.0 scale for OM. Response Evaluation Criteria in Solid Tumors (RECIST) based on magnetic resonance imaging (MRI) for treatment efficacy. |
|  | G3. Evaluation time | Weekly for OM in patients. Days 7, 14, 21 for animal model. |
|  | G4. Secondary outcomes | Reduction rate of T cells (CD3+, CD4+, CD8+), lymphocytes, hemoglobin, and weight. Inflammation (IL-1β, IL-6, TNF-α), apoptosis (Bax/Bcl-2), intestinal permeability (ZO-1, Claudin-1), TLR4/NF-κB in animal model. Gut microbiome diversity and composition. |
|  | G5. Associated clinical/laboratory biomarkers | Lymphocytes (CD3+, CD4+, CD8+), leukocytes. |
|  | G6. Associated lifestyle/medication factors | Not applicable. |
|  | G7. Associated host genetic factors | Not specified. |
| H. Key Results and Findings | H1. Key microbial taxa/species | Probiotics used: *Lactobacillus plantarum MH-301*, *Bifidobacterium animalis subsp. Lactis LPL-RH*, *Lactobacillus rhamnosus LGG-18*, *Lactobacillus acidophilus*. Gut microbiota: Firmicutes, Bacteroidetes, Proteobacteria, Actinobacteria, Lachnospiraceae, *Ruminococcus*, *Bacteroides* (in rats). *Lactobacillus*, *Bifidobacterium*, *Akkermansia* (in village residents). *Actinobacillus* (in NPC patients with severe mucositis). |
|  | H2. Direction of association | Probiotic: Reduced severe OM in patients (p < 0.01) and rats (p < 0.01). Improved reduction rate of CD3+, CD4+, CD8+ in patients (p < 0.01). Suppressed peripheral immune response in rats. Restored microbial diversity in patients (ARCP diverged, ARCPM clustered with HP). Reduced inflammation (IL-6, IL-1β, TNF-α) and apoptosis (Bax/Bcl-2), reversed TLR4/NF-κB upregulation, and improved ZO-1 and Claudin-1 expression in rats. |
|  | H3. Model predictive performance | Not applicable. |
|  | H4. Microbial diversity findings | In patients: CCRT perturbed microbiome diversity; probiotic cocktail restored dysbiosis. In rats: Probiotic cocktail improved alpha-diversity (Shannon, Simpson) and PCoA showed clustering with control. |
|  | H5. Microbial functional/metabolic findings | Not directly assessed. |
|  | H6. Preclinical/*in vitro* findings | Probiotics *MH-301*, *LPL-RH*, *LGG-18*, *L. acidophilus* selected based on acid, bile salt, oxidation resistance, antibacterial capacity, and cell adhesion. Animal model of OM reproduced findings of OM reduction and inflammatory/immune modulation. |
|  | H7. Highlight of Findings: Unique Contribution | This study demonstrates that gut microbiome modulation with a specific probiotic cocktail can significantly reduce the severity of oral mucositis (OM) induced by concurrent chemoradiotherapy (CCRT) in NPC patients. The "major discovery" is the elucidation of the biological mechanisms involved, showing that probiotics act by improving systemic immune response (increase in T cells) and restoring gut microbiome homeostasis, which in turn attenuates inflammation and protects intestinal barrier integrity. This validates a microbiome-based therapeutic approach to mitigate oncological treatment toxicities. |
|  | H8. Validation status (internal/external) | Internal validation (Phase II clinical trial). Validation in animal model (rats). |
| I. Discussion/Article Implications | I1. Authors' interpretation | The probiotic cocktail reduces OM severity by improving NPC patients' immunity and restoring gut dysbiosis. The mechanism involves inflammation suppression and intestinal barrier protection. |
|  | I2. Proposed biological mechanisms | Probiotics can: 1. Increase T cells (CD3+, CD4+, CD8+). 2. Reduce the activation of TLR4/NF-κB and the production of pro-inflammatory cytokines (IL-6, IL-1β, TNF-α). 3. Decrease apoptosis (Bax/Bcl-2). 4. Increase tight junction protein expression (ZO-1, Claudin-1), protecting the intestinal barrier. 5. Modulate microbiota composition (increase Firmicutes, Lachnospiraceae, *Ruminococcus* and decrease Bacteroidetes, *Actinobacillus*). |
|  | I3. Correlations with other biomarkers | Not explicitly discussed. |
|  | I4. Impact of interventions (if applicable) | The probiotic cocktail is an effective intervention for OM. Screening bacteria from cancer-free village residents led to the identification of promising probiotic strains. |
|  | I5. Consistency/Inconsistency of findings | Consistent with previous studies that show the microbiome's role in modulating immunotherapy and toxicity. Different from some studies that show probiotics like *Enterococcus faecium* as opportunistic pathogens. |
|  | I6. Transferability/Generalization | Findings are specific to NPC and CCRT-induced OM, but general mechanisms may be transferable to other treatment-induced oncological toxicities. |
|  | I7. Causality vs. Association | Mechanistic studies in rats provide strong evidence of causality for the probiotic cocktail's effects on OM. |
| J. Clinical Application Potential and Barriers | J1. Potential for implementation in practice | High potential for the use of probiotic cocktails as adjuvant treatment to mitigate OM in NPC patients undergoing CCRT. |
|  | J2. Advantages | Reduced OM severity, improved immune response, maintenance of intestinal bacterial homeostasis. |
|  | J3. Barriers to implementation | Need for more patients in the clinical trial to confirm results. Need for further studies on fecal microbiota translocation to identify the microbiome's role. |
|  | J4. Cost-effectiveness | Not discussed. |
|  | J5. Recommendations for future research | Increase the number of patients in the clinical trial. Perform fecal microbiota transplant to validate the microbiome's role. |
| K. Limitations and Bias of the Original Study | K1. Limitations declared by authors | Number of NPC patients was not large enough. Need for fecal microbiota transplant to further identify the microbiome's role. |
|  | K2. Selection bias | Not explicitly stated. |
|  | K3. Confounding bias | Not explicitly stated. |
|  | K4. Generalization | Study focused on NPC, may not be directly generalizable to other cancer patient populations. |
|  | K5. Reproducibility | Data available in online repositories (PRJNA579226). |

Table S7. Article 7: Grow With the Challenge – Microbial Effects on Epithelial Proliferation, Carcinogenesis, and Cancer Therapy

| Category | Extracted Variables | Detailing |
| --- | --- | --- |
| A. Study Identification | A1. Lead Author (Year) | von Frieling J (2018) |
|  | A2. Title | Grow With the Challenge – Microbial Effects on Epithelial Proliferation, Carcinogenesis, and Cancer Therapy |
|  | A3. Journal | Frontiers in Microbiology. |
|  | A4. Country/Region | Germany (Kiel) |
|  | A5. Funding | Deutsche Forschungsgemeinschaft (DFG) CRC1182 “Origin and Function of Metaorganisms” (C2, C1, A4, Z1), Nucleotide Lab of the ExC 306 Inflammation at Interfaces, Research Training Group “Genes, Environment, and Inflammation” (RTG 1743/1), intramural grant from the medical faculty of Kiel University. Type: Public. |
|  | A6. Conflicts of interest | No conflicts of interest declared by the authors. |
| B. Methodological Design | B1. Study type | Review |
|  | B2. Follow-up duration | Not applicable (review) |
|  | B3. Study center(s) | Not applicable (review) |
|  | B4. Recruitment period | Not applicable (review) |
|  | B5. Ethical approval | Not applicable (review) |
| C. Study Population | C1. Sample size | Not applicable (review of various studies in humans and animal models). |
|  | C2. Age | Not applicable |
|  | C3. Sex | Not applicable |
|  | C4. Cancer type | Colorectal carcinoma, bladder cancer, sarcoma, melanoma. Animal models: Colon cancer, MCA205 sarcoma. |
|  | C5. Tumor stage | Not specified (review). |
|  | C6. Performance status | Not applicable |
|  | C7. Relevant comorbidities | Intestinal inflammation. |
|  | C8. Previous treatments | Not applicable. |
| D. Chemotherapy Protocol | D1. Chemotherapy regimen | Oxaliplatin, 5-Fluorouracil (5-FU), Gemcitabine, Cyclophosphamide, Irinotecan, Doxorubicin. |
|  | D2. Treatment line | Not specified (review). |
|  | D3. Treatment intent | Chemotherapy, immunotherapy (CTLA-4 and PD-L1 blockade). |
|  | D4. Number of cycles | Not specified. |
|  | D5. Combination therapies | Immunotherapy (CTLA-4, PD-L1 blockade), chemotherapy. |
|  | D6. Dose modifications | Not specified. |
| E. Microbiome Analysis | E1. Sample type | Not specified (review). |
|  | E2. Timing of collection | Not specified. |
|  | E3. Sequencing technique | Not specified, but mentions metagenomics. |
|  | E4. Sequencing platform | Not specified. |
|  | E5. Sequencing depth | Not specified. |
|  | E6. Bioinformatic pipeline | Not specified. |
|  | E7. Reference database | Not specified. |
|  | E8. Quality control | Not specified. |
| F. Predictive Model | F1. Model type | Not directly applicable (review). Mentions the microbiome as a "promising target to prevent carcinogenesis and boost chemotherapy success rate." |
|  | F2. Algorithm(s) used | Not directly applicable (review). |
|  | F3. Predictive variables | Microbial taxa (*Fusobacterium nucleatum*, *Bacteroides fragilis*, *Prevotella* spp., *Mycoplasma hyorhinis*, Gammaproteobacteria, *Barnesiella intestinihominis*, *Enterococcus hirae*, *Lactobacillus johnsonii*, *Lactobacillus murinus*, *Bifidobacterium*), microbial metabolites (secondary bile acids, butyrate). |
|  | F4. Feature selection method | Not directly applicable (review). |
|  | F5. Model validation | Not directly applicable (review). |
|  | F6. Performance metrics | Not directly applicable (review). |
|  | F7. Training set size | Not applicable. |
|  | F8. Test set size | Not applicable. |
|  | F9. Model interpretability | Mechanisms of action of bacteria and metabolites. |
|  | F10. Model availability | Not applicable. |
| G. Outcomes Assessed | G1. Primary study outcome | Not applicable (review). |
|  | G2. Response criteria | Not specified (review). |
|  | G3. Evaluation time | Not specified. |
|  | G4. Secondary outcomes | Epithelial cell proliferation, carcinogenesis, chemotherapy efficacy, chemotherapy toxicity, immunotherapy response. |
|  | G5. Associated clinical/laboratory biomarkers | Not specified. |
|  | G6. Associated lifestyle/medication factors | Antibiotics (ABT) (abrogate antitumor effects, prevent cancer development), diet (high-fiber promotes protection). |
|  | G7. Associated host genetic factors | Not specified. |
| H. Key Results and Findings | H1. Key microbial taxa/species | Promoters of Carcinogenesis/Chemoresistance: *Fusobacterium nucleatum*, *Bacteroides fragilis* (also promotes immunotherapy), *Prevotella* spp., *Mycoplasma hyorhinis*, Gammaproteobacteria, *Streptococcus gallolyticus*, *Helicobacter pylori*, *Salmonella enterica*. Promoters of Efficacy/Protection: *Bacteroides thetaiotaomicron*, Burkholderiales, *Barnesiella intestinihominis*, *Enterococcus hirae*, *Lactobacillus johnsonii*, *Lactobacillus murinus*, *Bifidobacterium*. |
|  | H2. Direction of association | Dysbiosis: Altered in malignant tumors, increases microbial diversity in colorectal carcinoma (CRC). Carcinogenesis: *F. nucleatum*, *B. fragilis*, *Prevotella* spp. drive epithelial proliferation. Chemoresistance: *F. nucleatum* promotes via TLR4/MYD88; *Mycoplasma hyorhinis* and Gammaproteobacteria inactivate gemcitabine. Immunotherapy: *B. thetaiotaomicron* and *B. fragilis* essential for anti-CTLA-4; *Bifidobacterium* for anti-PD-L1. Efficacy: Oxaliplatin depends on infiltrating mycelium and TLR4-MYD88 signaling. Cyclophosphamide (CTX) depends on *Lactobacillus*, *Enterococcus hirae*, *B. intestinihominis*. |
|  | H3. Model predictive performance | Not applicable (review). |
|  | H4. Microbial diversity findings | In CRC, microbial diversity is increased. Co-habitation or fecal transfer can equalize tumor growth among mice. |
|  | H5. Microbial functional/metabolic findings | Xenobiotic metabolism by bacteria. Cytidine deaminase (CDDL) enzyme in Gammaproteobacteria inactivates gemcitabine. Bacterial metabolites can induce DNA damage. Butyrate protects against colorectal cancer via GPR109a and GPR43. Secondary bile acids (carcinogenic). |
|  | H6. Preclinical/*in vitro* findings | Germ-free mice or antibiotic-treated mice protected or had reduced cancer development. *Drosophila* as a high-throughput model for chemotherapeutics. |
|  | H7. Highlight of Findings: Unique Contribution | This review article consolidates the view that the microbiome is not just an environmental factor but an intrinsic and dynamic regulator of tissue homeostasis, carcinogenesis, and, crucially, the efficacy and toxicity of anticancer therapies. The "major discovery" is the demonstration that the microbiome actively modulates immune response and drug metabolism, becoming a manipulable target to optimize treatments. The article emphasizes that anticancer therapy success "depends on the microbiome," paving the way for the development of personalized and complementary strategies. |
|  | H8. Validation status (internal/external) | Not applicable (review). |
| I. Discussion/Article Implications | I1. Authors' interpretation | The microbiome has direct effects on cell proliferation and cell death, being crucial for tissue homeostasis. Imbalances can lead to cancer. The microbiome influences inflammation and, consequently, cancer susceptibility. The microbiome also influences therapy success. |
|  | I2. Proposed biological mechanisms | Regulation of intestinal stem cell activity (JNK, EGFR, Imd, JAK-STAT in *Drosophila*; TLR in mice). Drug metabolism (gemcitabine, 5-FU, cyclophosphamide). Modulation of immunity (helper T cells (Th17, Th1), cytotoxic T cells (CD8+), NK cells, dendritic cells). PAMPs signaling via TLRs. Production of inflammatory cytokines (TNF). |
|  | I3. Correlations with other biomarkers | Not directly discussed. |
|  | I4. Impact of interventions (if applicable) | Antibiotics can abrogate antitumor effects. Fecal microbiota transplant (FMT) can equalize tumor growth. |
|  | I5. Consistency/Inconsistency of findings | Effects of *B. fragilis* (carcinogenesis promoter and immunotherapy supporter) seem contradictory, but may be due to pleiotropic effects. |
|  | I6. Transferability/Generalization | The article suggests that *Drosophila* is a potential high-throughput model for studying the microbiome's impact on chemotherapeutic efficacy. |
|  | I7. Causality vs. Association | The review points to evidence of causality, especially in animal models (e.g., microbiome eradication with antibiotics abrogates response). |
| J. Clinical Application Potential and Barriers | J1. Potential for implementation in practice | Identification of specific bacteria and molecular pathways to prevent tumorigenesis or modulate chemotherapy response. |
|  | J2. Advantages | Targeted microbiome modulation can be a "promising approach" to personalize oncology. |
|  | J3. Barriers to implementation | The microbiome is complex and its composition differs between humans and animal models. Gaps remain regarding the cause-and-effect relationship. |
|  | J4. Cost-effectiveness | Not discussed. |
|  | J5. Recommendations for future research | Studies to elucidate fundamental principles of host-microbiota interactions. Exploration of diet, probiotic interventions. |
| K. Limitations and Bias of the Original Study | K1. Limitations declared by authors | The article does not declare specific limitations of the study itself (being a review), but points to knowledge gaps in the field, such as the need for more studies on the cause-and-effect relationship and the distinction between taxa. |
|  | K2. Selection bias | Not applicable (review). |
|  | K3. Confounding bias | Not applicable (review). |
|  | K4. Generalization | Not applicable (review). |
|  | K5. Reproducibility | Not applicable (review). |

Table S8. Article 8: The Relationship Between Gut Microbiome Features and Chemotherapy Response in Gastrointestinal Cancer

| Category | Extracted Variables | Detailing |
| --- | --- | --- |
| A. Study Identification | A1. Lead Author (Year) | Li N (2021) |
|  | A2. Title | The Relationship Between Gut Microbiome Features and Chemotherapy Response in Gastrointestinal Cancer |
|  | A3. Journal | Frontiers in Oncology. |
|  | A4. Country/Region | China (Beijing) |
|  | A5. Funding | National Natural Science Foundation of China (61435001), CAMS Innovation Fund for Medical Sciences (2017-I2M-4-003, 2016-I2M-1-001). Type: Public. |
|  | A6. Conflicts of interest | No conflicts of interest declared by the authors. |
| B. Methodological Design | B1. Study type | Prospective cohort study. |
|  | B2. Follow-up duration | Not specified (sample collection from April 2018 to April 2020). |
|  | B3. Study center(s) | Unicenter (Peking Union Medical College Hospital (PUMCH)). |
|  | B4. Recruitment period | April 2018 to April 2020. |
|  | B5. Ethical approval | PUMCH Ethics Committee. |
| C. Study Population | C1. Sample size | 130 gastrointestinal (GI) cancer patients, 147 healthy controls (HC). 117 patients with efficacy data, 53 patients with samples at two time points for variation analysis. |
|  | C2. Age | Patients: Median 63.5 years (range 29–75). HC: Median 55 years (range 22–74). |
|  | C3. Sex | Patients: 93 M / 37 F. HC: 84 M / 63 F. |
|  | C4. Cancer type | Esophageal Squamous Cell Carcinoma (EC), Gastric Adenocarcinoma (GC), Colorectal Adenocarcinoma (CRC). |
|  | C5. Tumor stage | Locally advanced (51/130) or with distant metastases (79/130) according to American Joint Committee on Cancer (AJCC) TNM 7.0. |
|  | C6. Performance status | Not specified. |
|  | C7. Relevant comorbidities | Excluded patients with inflammatory bowel disease, intestinal infection, intestinal obstruction, probiotic/antibiotic use in the past 2 months prior to collection. |
|  | C8. Previous treatments | Treatment-naive in the 6 months prior to admission. |
| D. Chemotherapy Protocol | D1. Chemotherapy regimen | Oxaliplatin-based regimens (53.1%), taxane+platinum (30.8%), irinotecan (3.8%), immunotherapy (4.6%), others (7.6%). |
|  | D2. Treatment line | Not specified, but patients with locally advanced or metastatic disease. |
|  | D3. Treatment intent | Not directly specified, but treatment for advanced/metastatic disease. |
|  | D4. Number of cycles | Not specified. Response evaluation 6-8 weeks after treatment. |
|  | D5. Combination therapies | Chemotherapy, targeted therapy, immunotherapy. |
|  | D6. Dose modifications | Not specified. |
| E. Microbiome Analysis | E1. Sample type | Feces. |
|  | E2. Timing of collection | Patients: 1 week before therapy (baseline) and at response evaluation ("treatment"). HC: One collection. |
|  | E3. Sequencing technique | 16S rRNA sequencing (V4 region). |
|  | E4. Sequencing platform | Illumina MiSeq. |
|  | E5. Sequencing depth | Total of 30,502,042 tags, mean of 89,712 tags per sample (340 samples). |
|  | E6. Bioinformatic pipeline | FLASH software (v1.2.11), USEARCH software (v7.0.1090), Ramer-Douglas-Peucker classifier algorithm (v2.2), R programming language (v3.1.1). |
|  | E7. Reference database | GreenGene Database (V201305). |
|  | E8. Quality control | Removal of low-quality reads, reads with N, low-complexity reads. Clustering of tags into Operational Taxonomic Units (OTU) with 97% similarity. |
| F. Predictive Model | F1. Model type | Machine Learning classifier to predict chemotherapy response. |
|  | F2. Algorithm(s) used | LEfSe (Linear Discriminant Analysis Effect Size), ROC (Receiver Operating Characteristic) curves, Kaplan-Meier estimate and log-rank test. |
|  | F3. Predictive variables | Microbial taxa (genera, species), abundance variation (post-treatment vs. baseline). |
|  | F4. Feature selection method | LEfSe (LDA score ≥ 3.6), Mann-Whitney U test, Kruskal-Wallis test. |
|  | F5. Model validation | Internal validation (70% training, 30% validation cross-validation) for the ROC model. |
|  | F6. Performance metrics | AUC, Sensitivity, Specificity (for ROC). |
|  | F7. Training set size | 37 samples for the ROC model. |
|  | F8. Test set size | 16 samples for the ROC model. |
|  | F9. Model interpretability | Cladograms, bar diagrams with LDA scores, Kaplan-Meier curves. |
|  | F10. Model availability | Not specified. |
| G. Outcomes Assessed | G1. Primary study outcome | Chemotherapy response (Responders (R) vs. Non-Responders (NR) / Disease Progression (PD) vs. non-PD). |
|  | G2. Response criteria | RECIST 1.1 (best clinical response: partial response (PR), stable disease (SD), progressive disease (PD)). |
|  | G3. Evaluation time | 6–8 weeks after treatment. |
|  | G4. Secondary outcomes | Progression-Free Survival (PFS), Overall Survival (OS). Toxicity (grade 3–4 AEs), ESR (Erythrocyte Sedimentation Rate) and hsCRP (hypersensitive C-reactive protein) elevation, lymphopenia, abnormal lymphocyte subgroups. |
|  | G5. Associated clinical/laboratory biomarkers | ESR, hsCRP, blood cell count (total lymphocytes and subgroups: B cells, CD4+ T, CD8+ T, NK (natural killer cell), CD4+CD28+T/CD4+T, CD8+CD38+T/CD8+T). |
|  | G6. Associated lifestyle/medication factors | Diet (Northern Chinese population with parallel dietary history), antibiotic/probiotic use (exclusion criteria). |
|  | G7. Associated host genetic factors | Not specified. |
| H. Key Results and Findings | H1. Key microbial taxa/species | In Patients (vs. HC): Increased: *Bacteroides fragilis*, *Escherichia coli*, *Akkermansia muciniphila*, *Clostridium hathewayi*, *Alistipes finegoldii*. Decreased: *Faecalibacterium prausnitzii*, *Roseburia faecis*, *Clostridium clostridioforme*, *Blautia producta*, *Bifidobacterium adolescent*, *Butyricicoccus pullicaecorum*. Associated with chemotherapy response: *Roseburia faecis* (abundance variation). |
|  | H2. Direction of association | In Patients (vs. HC): Fecal microbiota richness increased. Chemotherapy Response: Decreased abundance of *R. faecis* after chemotherapy associated with worse response (NR); increased abundance of *R. faecis* associated with better response (R). |
|  | H3. Model predictive performance | ROC for *R. faecis* abundance variation (PD vs. non-PD, total cohort): AUC = 0.818 (95% CI: 0.536–1.000), P = 0.040. Sensitivity = 75.0%, Specificity = 93.9%. |
|  | H4. Microbial diversity findings | GI cancer patients vs. HC: Chao1 index (richness) higher in patients (388.8 vs 377.0, P = 0.00217). Shannon index (homogeneity) similar (3.4 vs 3.3, P = 0.25785). Beta diversity (PCoA) showed significant difference (R = 0.282, P = 0.001). No significant difference in alpha or beta diversity between responders and non-responders at baseline. |
|  | H5. Microbial functional/metabolic findings | Not directly assessed, but *Roseburia* is a butyrate producer. |
|  | H6. Preclinical/*in vitro* findings | Not applicable. |
|  | H7. Highlight of Findings: Unique Contribution | This study establishes a clear gut microbial signature that distinguishes gastrointestinal (GI) cancer patients from healthy individuals and, crucially, identifies the variation in *Roseburia faecis* abundance as a predictive biomarker of chemotherapy response. The "major discovery" is the ability to monitor microbiome change (and not just baseline) to predict Disease Progression (PD), suggesting that the microbiome can serve as a non-invasive and easily performed tool to adapt treatments. This represents an advance towards precision medicine by considering microbiota dynamics in therapeutic response. |
|  | H8. Validation status (internal/external) | Internal validation (cross-validation for the ROC model). |
| I. Discussion/Article Implications | I1. Authors' interpretation | The gut microbiome of GI cancer patients differs from healthy individuals, and changes in *Roseburia faecis* abundance can predict chemotherapy response. The studied population, with similar dietary and geographical history, suggests that observed differences are attributable to the disease. |
|  | I2. Proposed biological mechanisms | *Roseburia* is a producer of butyrate (Short-Chain Fatty Acids (SCFAs)), which protects the intestine and inhibits carcinogenesis (blocks nuclear factor kappa beta (NF-κB), induces T cell activation, inhibits angiogenesis). |
|  | I3. Correlations with other biomarkers | Positive correlation between baseline *R. faecis* abundance and NK cell proportion (r = 0.329, P = 0.029). No correlation with TNM stage, sex, age, differentiation, hsCRP, total lymphocytes, or lymphocyte subgroups. |
|  | I4. Impact of interventions (if applicable) | Does not discuss interventions. |
|  | I5. Consistency/Inconsistency of findings | Findings of reduced *F. prausnitzii* in CRC are consistent with the literature. Reduced *Roseburia* in cancer patients is also consistent. Inconsistency in detecting significant changes in GC and CRC in separate analyses may be due to biological differences of the tumor or sample size. |
|  | I6. Transferability/Generalization | The microbial characteristics of EC and GC patients are similar to those of CRC patients. Most patients (50-80 years old) are within the common age range in articles. |
|  | I7. Causality vs. Association | The study establishes an association. Causality and exact mechanisms need to be verified by further functional and metabolomic research. |
| J. Clinical Application Potential and Barriers | J1. Potential for implementation in practice | High potential for *Roseburia faecis* as a biomarker to monitor disease progression and adjust treatment regimen. |
|  | J2. Advantages | Non-invasive and easy-to-perform test. |
|  | J3. Barriers to implementation | Need for more functional and metabolomic validation. Relatively small sample size, especially in subgroups. |
|  | J4. Cost-effectiveness | Not discussed. |
|  | J5. Recommendations for future research | Conduct metabolomic and animal studies to explore mechanisms in depth. |
| K. Limitations and Bias of the Original Study | K1. Limitations declared by authors | Relatively small sample size, especially in tumor type subgroups. Lack of metabolomic analysis (SCFAs). No animal research was conducted to validate clinical findings. Did not explore the exact mechanism of *Roseburia*. Microbiota heterogeneity and impact of other factors (food) not fully analyzed. |
|  | K2. Selection bias | Not explicitly stated. |
|  | K3. Confounding bias | Control for lifestyle factors, diet, and concomitant medications (beyond exclusion criteria) not specified. |
|  | K4. Generalization | The population is from China, which may limit generalization. The lack of significant findings in GC and CRC in separate analyses, unlike EC, may be a limitation. |
|  | K5. Reproducibility | Datasets available in NCBI with BioProject Number 766426. |

Table S9. Article 9: Gut microbiome with RAS mutation and chemotherapy response in patients with advanced or metastatic colorectal cancer: a pilot, exploratory study

| Category | Extracted Variables | Detailing |
| --- | --- | --- |
| A. Study Identification | A1. Lead Author (Year) | Kim JH (2025) |
|  | A2. Title | Gut microbiome with RAS mutation and chemotherapy response in patients with advanced or metastatic colorectal cancer: a pilot, exploratory study |
|  | A3. Journal | Frontiers in Oncology. |
|  | A4. Country/Region | South Korea |
|  | A5. Funding | Korea University, Korea Health Technology R&D Project (RS-2022-KH130153), Jeil Pharmaceutical Co., Ltd. Type: Public and Private. |
|  | A6. Conflicts of interest | No conflicts of interest declared by the authors. |
| B. Methodological Design | B1. Study type | Pilot exploratory prospective cohort study. |
|  | B2. Follow-up duration | Median of 15.9 months (95% CI: 13.49–18.27). |
|  | B3. Study center(s) | Unicenter (Korea University Anam Hospital). |
|  | B4. Recruitment period | October 2021 to February 2023. |
|  | B5. Ethical approval | Institutional Review Board (2021AN0403). |
| C. Study Population | C1. Sample size | 17 patients recruited. 15 patients with fecal samples (30 samples collected in total). |
|  | C2. Age | Median 61 years (range 35–73 years). |
|  | C3. Sex | Male: 47.1% (8/17); Female: 52.9% (9/17). |
|  | C4. Cancer type | Metastatic colorectal cancer (mCRC). |
|  | C5. Tumor stage | Locally advanced disease (1), initial metastasis (11), recurrent disease (5). |
|  | C6. Performance status | Eastern Cooperative Oncology Group (ECOG) scores of 0–2. |
|  | C7. Relevant comorbidities | Not specified. |
|  | C8. Previous treatments | No previous palliative systemic chemotherapy, no previous antibiotic use. |
| D. Chemotherapy Protocol | D1. Chemotherapy regimen | First-line systemic chemotherapy: 5-fluorouracil, leucovorin, and oxaliplatin (FOLFOX) or 5-fluorouracil, leucovorin, and irinotecan (FOLFIRI), with or without biological agents (anti-VEGF (vascular endothelial growth factor) or anti-EGFR (epidermal growth factor receptor)). |
|  | D2. Treatment line | First-line systemic. |
|  | D3. Treatment intent | Metastatic disease control. |
|  | D4. Number of cycles | Administered every 2 weeks until disease progression or unacceptable toxicity. |
|  | D5. Combination therapies | Chemotherapy + biological agents (anti-VEGF or anti-EGFR). |
|  | D6. Dose modifications | Not specified. |
| E. Microbiome Analysis | E1. Sample type | Feces. |
|  | E2. Timing of collection | At baseline (before chemotherapy), during chemotherapy, or at the time of disease progression. |
|  | E3. Sequencing technique | 16S rRNA sequencing. |
|  | E4. Sequencing platform | Not specified. |
|  | E5. Sequencing depth | Not specified. |
|  | E6. Bioinformatic pipeline | QIIME2R (v0.99.6), Microbial (v0.0.20), Microbiomeutilities (v1.00.17). |
|  | E7. Reference database | Not specified. |
|  | E8. Quality control | All samples met quality control requirements for 16S rRNA sequencing. |
| F. Predictive Model | F1. Model type | Microbial signature associated with chemotherapy response and mutation status. |
|  | F2. Algorithm(s) used | LEfSe (Linear Discriminant Analysis Effect Size), Kaplan-Meier. |
|  | F3. Predictive variables | Microbial taxa (genera *Holdemanella*, *Anaerostipes*, *Collinsella*, *Lactobacillus*, *Bacteroides*, *Bifidobacterium*, *Actinomyces*, *Solobacterium*, Butyricicoccaceae, Ruminococcaceae, *Faecalibacterium*, Lachnospiraceae_NK4A136_group, *Enterococcus*, *Peptostreptococcus*, *Streptococcus*). |
|  | F4. Feature selection method | LEfSe (LDA score > 2.0). |
|  | F5. Model validation | No predictive model with validation specified. The study is exploratory pilot. |
|  | F6. Performance metrics | Not applicable to a specific predictive model. |
|  | F7. Training set size | Not applicable. |
|  | F8. Test set size | Not applicable. |
|  | F9. Model interpretability | Differential abundance and diversity analysis. LEfSe cladograms. |
|  | F10. Model availability | Not applicable. |
| G. Outcomes Assessed | G1. Primary study outcome | Gut microbiome composition in relation to lifestyle factors, clinicogenomic factors, and chemotherapy response. |
|  | G2. Response criteria | RECIST 1.1 (Complete Response (CR), Partial Response (PR), Stable Disease (SD), Progressive Disease (PD)). |
|  | G3. Evaluation time | Every 6 weeks by CT or MRI. |
|  | G4. Secondary outcomes | Progression-Free Survival (PFS), Overall Survival (OS). |
|  | G5. Associated clinical/laboratory biomarkers | KRAS, NRAS, BRAF mutation status; Microsatellite Instability (MSI) status. |
|  | G6. Associated lifestyle/medication factors | Smoking history, alcohol consumption history, previous radiotherapy. |
|  | G7. Associated host genetic factors | RAS mutation (KRAS and NRAS), BRAF, Microsatellite Instability (MSI). |
| H. Key Results and Findings | H1. Key microbial taxa/species | Smoking: *Actinomyces* and *Solobacterium* (smokers); Butyricicoccaceae, Ruminococcaceae, *Faecalibacterium*, Lachnospiraceae_NK4A136_group (non-smokers). RAS mutation: *Holdemanella*, *Anaerostipes*, *Collinsella* (RAS-mutated mCRC); *Faecalibacterium*, *Eubacterium* (wild-type RAS mCRC). Chemotherapy Response: *Lactobacillus* spp. (responders); *Anaerococcus*, Christensenellaceae, DTU089, *Porphyromonas* (non-responders). Disease Control: *Bifidobacterium* (non-PD); *Bacteroides* (trend of increase in PD). |
|  | H2. Direction of association | Smoking: Changes in microbiome composition (but not alpha diversity). RAS mutation: Notable differences in beta diversity (P = 0.042 and P = 0.047). *Holdemanella*, *Anaerostipes*, *Collinsella*, and Coriobacteriaceae enriched in mutated RAS. Chemotherapy Response: *Lactobacillus* spp. in responders. *Bifidobacterium* significantly increased in non-PD (P = 0.0027). *Bacteroides* tended to increase in PD (P = 0.2065). |
|  | H3. Model predictive performance | Not applicable (exploratory pilot study, not a validated predictive model). |
|  | H4. Microbial diversity findings | Smoking: Beta diversity showed a weak difference (P = 0.058 and P = 0.056). RAS mutation: No significant change in alpha diversity, but notable differences in beta diversity. Chemotherapy Response: No difference in alpha or beta diversity between responders and non-responders. |
|  | H5. Microbial functional/metabolic findings | Not directly assessed. |
|  | H6. Preclinical/*in vitro* findings | Not applicable. |
|  | H7. Highlight of Findings: Unique Contribution | This pilot study is the first to investigate the interconnections among the gut microbiome, RAS mutations, and chemotherapy response in mCRC. The "major discovery" is the suggestion of an association between RAS mutation status and gut microbiome composition, with specific taxa enriched in each state, and that this association may influence chemotherapy response. This opens a new avenue to explore the microbiome as a biomarker that integrates host genetic factors and environmental factors (smoking) to predict treatment outcomes and personalize approaches for mCRC patients. |
|  | H8. Validation status (internal/external) | Not applicable (exploratory pilot study). |
| I. Discussion/Article Implications | I1. Authors' interpretation | The gut microbiome may play a mediating role in the impact of smoking on mCRC. The differences in the microbiome between RAS-mutated and wild-type RAS mCRC may explain prognostic differences. The microbiome can serve as a biomarker of response. |
|  | I2. Proposed biological mechanisms | The microbiome can cause DNA damage, promoting genomic instability and increased mutation rate (e.g., colibactin from *E. coli*, *H. pylori*). Taxa like *Faecalibacterium* and *Eubacterium* are protective, while *Anaerostipes* and *Bacteroides* are detrimental. |
|  | I3. Correlations with other biomarkers | KRAS, NRAS, BRAF mutation status; Microsatellite Instability (MSI) status. |
|  | I4. Impact of interventions (if applicable) | Not discussed. |
|  | I5. Consistency/Inconsistency of findings | Findings on smoking and microbiome are in line with literature. The relationship between KRAS and microbiome has been reported (*Roseburia*, *Parabacteroides*, *Metascardovia*, *Staphylococcus*, Bacillales). |
|  | I6. Transferability/Generalization | The studied population is Korean of East Asian descent, which may limit generalization. |
|  | I7. Causality vs. Association | The study identifies associations. More functional studies are needed to elucidate the causal role of microorganisms. |
| J. Clinical Application Potential and Barriers | J1. Potential for implementation in practice | Promising biomarker to predict treatment response and prognosis in mCRC patients, aiding therapy selection and personalization. |
|  | J2. Advantages | Non-invasive, can complement other biomarkers. |
|  | J3. Barriers to implementation | Small sample size of the pilot study. Need for validation in larger, independent cohorts. Lack of data on confounding factors (socioeconomic status, concomitant medications, detailed dietary patterns). |
|  | J4. Cost-effectiveness | Not discussed. |
|  | J5. Recommendations for future research | Larger and more detailed studies to validate associations. Integration of shotgun metagenomics, metabolomics, and functional validation. |
| K. Limitations and Bias of the Original Study | K1. Limitations declared by authors | Small sample size limits statistical power and robustness. Korean population of East Asian descent limits generalization. Missing data on confounding factors (socioeconomic status, marital status, medications/supplements, detailed diet). Timing of follow-up sample collection was not standardized. No mechanistic experiments were performed to investigate the influence of microbes on RAS-related pathways. |
|  | K2. Selection bias | Not explicitly stated. |
|  | K3. Confounding bias | Environmental factors (smoking, diet) are not completely explained by microbiome composition. |
|  | K4. Generalization | The population is Korean, which may limit generalization to other ethnic groups. |
|  | K5. Reproducibility | Datasets available in NCBI with PRJNA1214585. |

Table S10. Article 10: Gut microbiome model predicts response to neoadjuvant immunotherapy plus chemoradiotherapy in rectal cancer

| Category | Extracted Variables | Detailing |
| --- | --- | --- |
| A. Study Identification | A1. Lead Author (Year) | Yang Z (2024) |
|  | A2. Title | Gut microbiome model predicts response to neoadjuvant immunotherapy plus chemoradiotherapy in rectal cancer |
|  | A3. Journal | Med. |
|  | A4. Country/Region | China (Beijing, Tianjin, Xi'an) |
|  | A5. Funding | China National Natural Science Foundation (82202884), China Association of Gerontology and Geriatrics, Training Fund for Open Projects at Clinical Institutes and Departments of Capital Medical University (CCMU2022ZKYXY008), Beijing Hospitals Authority Clinical Medicine Development of special funding support (ZLRK202302), National Key Technologies R&D Program (2015BAI13B09), Clinical Center for Colorectal Cancer, Capital Medical University (1192070313). Type: Public. |
|  | A6. Conflicts of interest | No conflicts of interest declared by the authors. |
| B. Methodological Design | B1. Study type | Prospective multicenter cohort study (Phase II clinical trial). |
|  | B2. Follow-up duration | Median of 57.4 weeks (25.6–109.6) until September 2023. |
|  | B3. Study center(s) | Multicenter (6 centers). |
|  | B4. Recruitment period | June 2021 to November 2022. |
|  | B5. Ethical approval | Beijing Friendship Hospital Ethics Committee, Capital Medical University (2021-P2-318-01). Registration: ClinicalTrials.gov (NCT04911517 and NCT05368051). |
| C. Study Population | C1. Sample size | 60 patients screened, 50 patients recruited. 33 patients with fecal samples for analysis. |
|  | C2. Age | Not specified (mean/median). |
|  | C3. Sex | Not specified (distribution). |
|  | C4. Cancer type | Locally advanced rectal cancer (LARC). |
|  | C5. Tumor stage | LARC (T3-4 or N+, T3a, T3b, T3c, T4a, T4b). All Microsatellite Stable (MSS)/Proficient Mismatch Repair (pMMR). |
|  | C6. Performance status | Not specified (mentions Eastern Cooperative Oncology Group (ECOG) performance). |
|  | C7. Relevant comorbidities | Excluded with previous active malignancy, recent surgery, conditions affecting capecitabine absorption, uncontrolled severe infections, allergy to study components, estimated survival <5 years, severe hepatic/renal damage, previous immunotherapy, organ/bone marrow transplant, immunodeficiency, history of uncontrolled epilepsy, central neurological/mental disease. |
|  | C8. Previous treatments | Excluded patients who received immunosuppressants or systemic hormonal therapy 1 month before the study. |
| D. Chemotherapy Protocol | D1. Chemotherapy regimen | Capecitabine (850–1,000 mg/m2, bid, po, days 1–14). |
|  | D2. Treatment line | Neoadjuvant. |
|  | D3. Treatment intent | Neoadjuvant. |
|  | D4. Number of cycles | Three 21-day cycles of capecitabine. |
|  | D5. Combination therapies | Immunotherapy (tislelizumab - PD-1 (Programmed Cell Death Protein 1) blocker, 200 mg, iv.gtt, day 8) + chemoradiotherapy (long-course radiotherapy 50 Gy/25f, 2 Gy/f, 5 days/week + capecitabine). |
|  | D6. Dose modifications | Not specified. |
| E. Microbiome Analysis | E1. Sample type | Feces. |
|  | E2. Timing of collection | Pre-treatment (Pre), before the third 21-day cycle of neoadjuvant therapy (Post1), and before surgery (Post2). |
|  | E3. Sequencing technique | Metagenomics. |
|  | E4. Sequencing platform | Illumina PE150 (NovaGene). |
|  | E5. Sequencing depth | Total of 75 fecal samples collected. |
|  | E6. Bioinformatic pipeline | Readfq (v8), Bowtie 2 (v2.2.4), MEGAHIT (v1.0.4-beta), MetaGeneMark (v2.10), CD-HIT (v4.5.8), DIAMOND (v0.9.9.110), MEGAN, Metastats, LEfSe (Linear Discriminant Analysis Effect Size), Gephi, NetShift. |
|  | E7. Reference database | NCBI NR database (v2018-01-02). |
|  | E8. Quality control | High-quality clean reads, human DNA removal (*Homo sapiens* genome assembly hg38), gene dedundancy (CD-HIT). Scaftigs ≥500 bp. |
| F. Predictive Model | F1. Model type | Predictive model based on gut microbiome (SPEED - Species-level Gut Microbiome Prediction; GEED - Genus-level Gut Microbiome Prediction). |
|  | F2. Algorithm(s) used | Support Vector Machine (SVM) with linear kernel. |
|  | F3. Predictive variables | Microbial taxa (species and genera). |
|  | F4. Feature selection method | Based on the absolute value of species and genera, discarding variables expressed in <50% of patients in training. |
|  | F5. Model validation | Internal validation (5 repetitions of 5-fold cross-validation). External validation (independent cohorts of CRC, melanoma, NSCLC). |
|  | F6. Performance metrics | Area Under the Curve (AUC), accuracy (ACC), sensitivity (SE), specificity (SP), Positive Predictive Value (PPV), Negative Predictive Value (NPV). |
|  | F7. Training set size | 33 samples (14 responders, 19 non-responders). |
|  | F8. Test set size | Combined validation cohort: 50 patients (CRC: 9, NSCLC: 14, melanoma: 27). |
|  | F9. Model interpretability | Variable coefficients in the model (Tables S3 and S4), cladograms, co-occurrence networks. |
|  | F10. Model availability | Not specified. |
| G. Outcomes Assessed | G1. Primary study outcome | Pathological Complete Response (pCR) to neoadjuvant immunotherapy combined with chemoradiotherapy (nICRT). |
|  | G2. Response criteria | Tumor Regression Grade (TRG) 0 for pCR, TRG 1, 2, 3 for non-pCR (AJCC standard). |
|  | G3. Evaluation time | After radical surgery (6–12 weeks after radiotherapy completion). |
|  | G4. Secondary outcomes | Treatment-Related Adverse Events (TRAEs), survival (without recurrence or metastasis), dynamic microbiome variation. |
|  | G5. Associated clinical/laboratory biomarkers | Carcinoembryonic Antigen (CEA), Neutrophil-to-Lymphocyte Ratio (NLR), Platelet-to-Lymphocyte Ratio (PLR), Lymphocyte-to-Monocyte Ratio (LMR), Systemic Immune-Inflammation Index (SII), Mismatch Repair (MMR)/Microsatellite Stability (MSI) status. |
|  | G6. Associated lifestyle/medication factors | Not specified (exclusion of previous probiotic/antibiotic use). |
|  | G7. Associated host genetic factors | MSS (Microsatellite Stable)/pMMR (Proficient Mismatch Repair). |
| H. Key Results and Findings | H1. Key microbial taxa/species | After nICRT: *Lactobacillus*, *Eubacterium*, Lachnospiraceae, *Lachnoclostridium*. In responders (Rs) vs non-responders (NRs) at baseline: Rs: *Lachnospiraceae bacterium*, *Blautia wexlerae*, Clostridiales, *Clostridioides difficile*. NRs: *Bacteroides*, *Prevotella*, *Porphyromonas*, *Prevotella copri*, *Akkermansia muciniphila*, *Bacteroides plebeius*. Taxa with highest coefficient in SPEED model (e.g., positive impact on response): *Lachnospiraceae bacterium*, *Bacteroides intestinalis*, *Eubacterium sp. 41_20*, *Butyricimonas sp. MarseilleP3923*, *Candidatus gastranaerophilales bacterium HUM_17*, *Clostridium sp. CAG:58*, *Selenomonas ruminantium*. Taxa with highest coefficient in SPEED model (e.g., positive impact on non-response): *Phascolarctobacterium succinatutens*, *Fusobacterium nucleatum*, *Firmicutes bacterium CAG:65*. |
|  | H2. Direction of association | After nICRT: Microbial diversity decreases. Bacteroidetes reduced; Firmicutes, *Lactobacillus*, *Eubacterium*, *Lachnoclostridium* increased. In Rs at baseline: Higher abundance of *Lachnospiraceae bacterium*, *Blautia wexlerae*, Clostridiales, *Clostridioides difficile*. In NRs at baseline: Higher abundance of *Bacteroides*, *Prevotella*, *Porphyromonas*, *Prevotella copri*, *Akkermansia muciniphila*, *Bacteroides plebeius*. |
|  | H3. Model predictive performance | SPEED (species level): Training: AUC = 98.80% (95% CI: 95.67%–100%). Combined validation: AUC = 77.78% (95% CI: 65.42%–88.29%). CRC validation: AUC = 83.34%. Melanoma validation: AUC = 78.16%. NSCLC validation: AUC = 77.78%. GEED (genus level): Training: AUC = 81.34% (95% CI: 65.49%–93.73%). Combined validation: AUC = 70.77% (95% CI: 57.49%–81.27%). CRC validation: AUC = 77.78%. Melanoma validation: AUC = 66.67%. NSCLC validation: AUC = 64.81%. |
|  | H4. Microbial diversity findings | Diversity (Shannon and Inverse Simpson) decreases after nICRT. No significant difference in alpha diversity between Rs and NRs at baseline. |
|  | H5. Microbial functional/metabolic findings | *Eubacterium venturium* (Short-Chain Fatty Acid (SCFA) producer), *Bacteroides* spp. (butyrate and other substance producers). |
|  | H6. Preclinical/*in vitro* findings | Not applicable. |
|  | H7. Highlight of Findings: Unique Contribution | This pioneering study not only demonstrates a high pathological complete response (pCR) rate to neoadjuvant immunotherapy combined with chemoradiotherapy (nICRT) in rectal cancer but also, for the first time, develops and validates a species-level predictive model (SPEED) based on the gut microbiome. The "major discovery" is the SPEED model's ability to predict pCR with high robustness and accuracy from the baseline microbiome, revealing specific taxa associated with responders and non-responders. This establishes a new paradigm for patient stratification and individualized management of rectal cancer, optimizing treatment and the potential for "watch-and-wait" approaches. |
|  | H8. Validation status (internal/external) | Internal validation (training cohort). External validation (independent cohorts of CRC, melanoma and NSCLC). |
| I. Discussion/Article Implications | I1. Authors' interpretation | nICRT has an encouraging curative effect with mild toxicity. The gut microbiome may have predictive potential for nICRT response. The SPEED model is a reliable and repeatable tool for screening responders. |
|  | I2. Proposed biological mechanisms | SCFAs (produced by *Eubacterium venturium*) can provide energy, regulate regulatory T cells (Treg), and induce interleukin-18, improving immunotherapy efficacy. *Clostridioides difficile* may decrease and delay tumor progression. |
|  | I3. Correlations with other biomarkers | Higher Carcinoembryonic Antigen (CEA) levels were observed in the non-responder group (P = 0.017). No significant differences in other biomarkers. |
|  | I4. Impact of interventions (if applicable) | Does not discuss interventions. |
|  | I5. Consistency/Inconsistency of findings | The abundance of Firmicutes (40.99%) and Bacteroidetes (14.28%) reflects similarity with healthy adult microbial communities. Firmicutes associated with better outcomes, Bacteroidetes with adverse events. |
|  | I6. Transferability/Generalization | The SPEED model can guide clinical application with interpretability and usability. The reported method is not limited to LARC and can predict efficacy in other patients. |
|  | I7. Causality vs. Association | The study identifies predictive associations. |
| J. Clinical Application Potential and Barriers | J1. Potential for implementation in practice | The SPEED model can act as a reliable tool for screening responders, aiding individualized management of LARC patients, especially for considering the "watch-and-wait" strategy. |
|  | J2. Advantages | High robustness and accuracy in pCR prediction. Accessibility, non-invasiveness, and repeatability of fecal sampling. |
|  | J3. Barriers to implementation | A follow-up phase is still ongoing, requiring more long-term survival data. High costs and difficulty of using bioinformatic software for metagenomic sequencing in routine clinical practice. |
|  | J4. Cost-effectiveness | Not discussed, but metagenomic sequencing costs are higher. |
|  | J5. Recommendations for future research | Larger randomized clinical trials (Phase II/III) to confirm the benefits of nICRT and the predictive performance of the SPEED model. Establish gender, ethnicity, and age-stratified cohorts. Research the exact biological mechanism of the model. |
| K. Limitations and Bias of the Original Study | K1. Limitations declared by authors | Non-randomized Phase II study with relatively small sample size (33 fecal samples). Lack of long-term survival data (follow-up ongoing). Differences in lifestyle and microbiome among ethnic groups (need for stratified cohorts). Lack of research on the exact biological mechanism of the SPEED model. |
|  | K2. Selection bias | MSS/pMMR patients (which may limit generalization). |
|  | K3. Confounding bias | The impact of lifestyle factors such as diet was not addressed. |
|  | K4. Generalization | Chinese population, may limit global generalization. |
|  | K5. Reproducibility | Metagenomic sequencing data deposited at NCBI SRA BioProject (PRJNA1049662, PRJNA1054290). |

Table S11. Article 11: Gut Microbiota Modulation of Efficacy and Toxicity of Cancer Chemotherapy and Immunotherapy

| Category | Extracted Variables | Detailing |
| --- | --- | --- |
| A. Study Identification | A1. Lead Author (Year) | Chrysostomou D (2023) |
|  | A2. Title | Gut Microbiota Modulation of Efficacy and Toxicity of Cancer Chemotherapy and Immunotherapy |
|  | A3. Journal | Gastroenterology. |
|  | A4. Country/Region | United Kingdom |
|  | A5. Funding | Servier Technologies (through the Imperial College London STRATiGRAD Programme), Medical Research Council. Funding and infrastructure support from NIHR Imperial Biomedical Research Centre (BRC) (RDA02) and National Institute for Health Research (CL-2019-21-002). Type: Public and Private. |
|  | A6. Conflicts of interest | No conflicts of interest declared by the authors. |
| B. Methodological Design | B1. Study type | Review |
|  | B2. Follow-up duration | Not applicable (review). |
|  | B3. Study center(s) | Not applicable (review). |
|  | B4. Recruitment period | Not applicable (review). |
|  | B5. Ethical approval | Not applicable (review). |
| C. Study Population | C1. Sample size | Not applicable (review of various studies in humans and animal models). |
|  | C2. Age | Not applicable |
|  | C3. Sex | Not applicable |
|  | C4. Cancer type | Colorectal Cancer, Lung Cancer, Breast Cancer, Ovarian Cancer, Melanoma, Hepatocellular Carcinoma (HCC), Renal Cell Carcinoma. |
|  | C5. Tumor stage | Not specified (review). |
|  | C6. Performance status | Not applicable |
|  | C7. Relevant comorbidities | Graft-versus-host disease (GVHD). |
|  | C8. Previous treatments | Not applicable. |
| D. Chemotherapy Protocol | D1. Chemotherapy regimen | 5-Fluorouracil (5-FU), Irinotecan, Oxaliplatin, Gemcitabine, Methotrexate, Cisplatin, Cyclophosphamide (CTX). |
|  | D2. Treatment line | Not specified (review). |
|  | D3. Treatment intent | Chemotherapy, immunotherapy (anti-PD-1, anti-PD-L1, anti-CTLA-4). |
|  | D4. Number of cycles | Not specified. |
|  | D5. Combination therapies | Immunotherapy (checkpoint inhibitors) with or without chemotherapy. |
|  | D6. Dose modifications | Mentioned that toxicity may lead to dose reduction or cessation. |
| E. Microbiome Analysis | E1. Sample type | Feces. |
|  | E2. Timing of collection | Not specified. |
|  | E3. Sequencing technique | Not specified. |
|  | E4. Sequencing platform | Not specified. |
|  | E5. Sequencing depth | Not specified. |
|  | E6. Bioinformatic pipeline | Not specified. |
|  | E7. Reference database | Not specified. |
|  | E8. Quality control | Not specified. |
| F. Predictive Model | F1. Model type | Not directly applicable (review). Host-oncomicrobiome-therapy interaction models ("TIMER hypothesis"). |
|  | F2. Algorithm(s) used | Not directly applicable (review). |
|  | F3. Predictive variables | Microbial taxa (e.g., g-proteobacteria, *Mycoplasma hyorhinis*, *E. coli*, *Fusobacterium nucleatum*, *Lactobacillus johnsonii*, *Enterococcus hirae*, *Barnesiella intestinihominis*, *Bifidobacterium*, *Akkermansia muciniphila*, *Faecalibacterium prausnitzii*, *Gemmiger formicilis*, *Bacteroides fragilis*, *B. thetaiotaomicron*, Burkholderiales, *Collinsella aerofaciens*), microbial metabolites (e.g., Short-Chain Fatty Acids (SCFAs), bile acids, inosine), microbial enzymes (β-glucuronidase, cytidine deaminase, nitroreductase, thymidine phosphorylase). |
|  | F4. Feature selection method | Not directly applicable (review). |
|  | F5. Model validation | Not directly applicable (review). |
|  | F6. Performance metrics | Not directly applicable (review). |
|  | F7. Training set size | Not applicable. |
|  | F8. Test set size | Not applicable. |
|  | F9. Model interpretability | Mechanisms of action of bacteria and metabolites. |
|  | F10. Model availability | Not applicable. |
| G. Outcomes Assessed | G1. Primary study outcome | Efficacy and toxicity of chemotherapy and immunotherapy. |
|  | G2. Response criteria | Not specified (review). |
|  | G3. Evaluation time | Not specified. |
|  | G4. Secondary outcomes | Survival, intestinal mucositis, diarrhea, peripheral neuropathy, nephropathy, cardiotoxicity, colitis. |
|  | G5. Biomarkers clinical/laboratory associated | Not specified. |
|  | G6. Associated lifestyle/medication factors | Antibiotic use (impacts efficacy/toxicity), diet (high-fiber, low-fat, ketogenic diets), probiotics, prebiotics, synbiotics. |
|  | G7. Associated host genetic factors | Toll-like receptors (TLR), MYD88, A2A receptor, STING. |
| H. Key Results and Findings | H1. Key microbial taxa/species | Modulate Chemotherapy: g-proteobacteria, *Mycoplasma hyorhinis*, *Escherichia coli*, *Fusobacterium nucleatum*, *Lactobacillus johnsonii*, *Enterococcus hirae*, *Barnesiella intestinihominis*. Modulate Immunotherapy: *Bifidobacterium*, *Lactobacillus rhamnosus GG*, *Akkermansia muciniphila*, *Enterococcus hirae*, *Bacteroides fragilis*, *B. thetaiotaomicron*, Burkholderiales, *Faecalibacterium prausnitzii*, *Gemmiger formicilis*, *Bifidobacterium longum*, *Collinsella aerofaciens*. |
|  | H2. Direction of association | Efficacy: Bacterial metabolites (e.g., butyrate) can increase. Specific bacteria can inactivate (e.g., g-proteobacteria inactivate gemcitabine) or activate (e.g., *E. coli* activates CB1954) chemotherapeutics. CTX (cyclophosphamide) depends on Gram-positive bacteria. Anti-CTLA-4 depends on *Bacteroides*. Anti-PD-1/PD-L1 depends on *Bifidobacterium*, *L. rhamnosus GG*, *A. muciniphila*. Toxicity: Bacterial β-glucuronidases (irinotecan) increase. Microbiome can inhibit (e.g., TLR2 for methotrexate) or increase (e.g., bacterial LPS via TLR4 for oxaliplatin) toxicity. Chemotherapy-induced dysbiosis can increase pathobionts. |
|  | H3. Model predictive performance | Not applicable (review). |
|  | H4. Microbial diversity findings | Loss of gut microbial diversity has a direct impact on chemotherapy efficacy and toxicity. Chemotherapy can induce dysbiosis and competitive inhibition failure. |
|  | H5. Microbial functional/metabolic findings | Microbial enzymes (β-glucuronidase, cytidine deaminase, nitroreductase, thymidine phosphorylase). Microbial metabolites (SCFAs, bile acids, inosine) can affect cancer cell and immune cell function. |
|  | H6. Preclinical/*in vitro* findings | Ample evidence from murine models (germ-free, antibiotic-treated) for almost all findings. *E. coli* in *Caenorhabditis elegans*. |
|  | H7. Highlight of Findings: Unique Contribution | This review article deepens the concept of "oncomicrobiome" and its pivotal role in modulating the efficacy and toxicity of anticancer therapies. The "major discovery" is the systematization of interactions through five central mechanisms (TIMER: Translocation, Immunomodulation, Metabolism, Enzymatic Degradation, and Reduced Diversity), which, by being expanded to include microbiome evolution throughout treatment, offer a multiparametric and dynamic model of pharmacomicrobiomics. This establishes a new paradigm for oncological research, emphasizing the microbiome as a promising therapeutic target for precision medicine, capable of shaping clinical outcomes and reducing treatment toxicity. |
|  | H8. Validation status (internal/external) | Not applicable (review). |
| I. Discussion/Article Implications | I1. Authors' interpretation | The microbiome has extensive drug metabolism capabilities and regulates the tumor microenvironment. It is a missing link that explains variation in therapeutic response. Microbiome modulation can be a crucial adjunct to current treatments. |
|  | I2. Proposed biological mechanisms | Microbial enzymes (β-glucuronidases) transform prodrugs. g-proteobacteria inactivate gemcitabine. *Bifidobacterium* regulates type I interferon via STING. *E. coli* activates CB1954. *Fusobacterium nucleatum* promotes chemoresistance via TLR4 and MYD88. Microbes influence T cells (Th17, Th1, CD8+). Toll-like receptors (TLR) and pathogen-associated molecular patterns (PAMPs) mediate innate immunity. |
|  | I3. Correlations with other biomarkers | Not directly discussed. |
|  | I4. Impact of interventions (if applicable) | Diet: Can improve efficacy (e.g., butyrate in irinotecan) and reduce toxicity (e.g., isomalto-oligosaccharides in irinotecan). Fasting/fasting-mimicking diet can reduce AEs and improve efficacy. Probiotics/Prebiotics/Synbiotics: Can mitigate toxicity (e.g., VSL#3 in irinotecan, *Lactobacillus* in cisplatin) and improve response. Postbiotics: Safer alternative to live probiotics. Antibiotics: Can be used to selectively abolish pathogenic bacteria exerting negative effects. Viruses, Fungi, Bacteriophages: Can inactivate tumors. Synthetic Engineering: Modification of bacteria to release cytotoxic compounds or modulate the immune system. Intestinal Microbiota Transplant (IMT): Promising for improving response and reducing toxicity (e.g., anti-PD-1 refractory melanoma). |
|  | I5. Consistency/Inconsistency of findings | The microbiome is a "mosaic" of microbiota-regulated toxicity. Inconsistencies of data on SCFAs and immunotherapy. |
|  | I6. Transferability/Generalization | Not explicitly discussed, but findings are derived from various models and cancers, suggesting broad applicability. |
|  | I7. Causality vs. Association | The review presents strong evidence of causality, especially in preclinical models, and explores the underlying mechanisms. |
|  | I8. Implications for treatment | Integration of the microbiome in drug trials and clinical practice is necessary to optimize therapies and reduce toxicity. |
| J. Clinical Application Potential and Barriers | J1. Potential for implementation in practice | The discovery of new therapeutic targets and the reduction of failures of candidates for new drugs. |
|  | J2. Advantages | Optimization of chemotherapy and immunotherapy, reduction of toxicity, development of personalized treatment strategies. |
|  | J3. Barriers to implementation | Need for robust clinical study data to overcome quality, safety, and efficacy challenges of Live Biotherapeutic Products (LBPs). Adoption of strategies must consider safety and efficiency, as there are conflicting results in different cohorts. |
|  | J4. Cost-effectiveness | Not discussed. |
|  | J5. Recommendations for future research | Future clinical trials should consider the interaction of genetic, environmental, and microbiome factors. New large-scale biobanks that consider global variations. Better understanding of underlying microbiome-drug interaction mechanisms. |
|  | J6. Personalization potential | Patient stratification and biomarker development for precision medicine. |
| K. Limitations and Bias of the Original Study | K1. Limitations declared by authors | Review, does not develop original study with its own limitations. |
|  | K2. Selection bias | Not applicable (review). |
|  | K3. Confounding bias | Not applicable (review). |
|  | K4. Generalization | Not applicable (review). |
|  | K5. Reproducibility | Not applicable (review). |

Table S12. Article 12: Gut microbiota-mediated nucleotide synthesis attenuates the response to neoadjuvant chemoradiotherapy in rectal cancer

| Category | Extracted Variables | Detailing |
| --- | --- | --- |
| A. Study Identification | A1. Lead Author (Year) | Teng H (2023) |
|  | A2. Title | Gut microbiota-mediated nucleotide synthesis attenuates the response to neoadjuvant chemoradiotherapy in rectal cancer |
|  | A3. Journal | Cancer Cell. |
|  | A4. Country/Region | China (Beijing) |
|  | A5. Funding | National Natural Science Foundation of China (81988101, 81830086, 82073333, 82173152), Science Foundation of Peking University Cancer Hospital (17-01), Beijing Hospitals Authority’s Ascent Plan (DFL20220902), Pilot Project (Fourth Round) to Reform Public Development of Beijing Municipal Medical Research Institute (2021-1), Chinese Academy of Medical Sciences Innovation Fund for Medical Sciences (2019-I2M-5-081). Type: Public. |
|  | A6. Conflicts of interest | The authors declare no conflicts of interest. |
| B. Methodological Design | B1. Study type | Longitudinal study with 16S rRNA sequencing and meta-transcriptomics (humans), metabolite profiling, and *in vitro* and *in vivo* (animal model) functional validation. |
|  | B2. Follow-up duration | Not specified. |
|  | B3. Study center(s) | Unicenter (Peking University Cancer Hospital and Institute). |
|  | B4. Recruitment period | January 2018 to November 2019. |
|  | B5. Ethical approval | Beijing Cancer Hospital Ethics Committee (No. 2018YJZ40). |
| C. Study Population | C1. Sample size | Humans: 126 Locally Advanced Rectal Cancer (LARC) patients. 353 fecal samples (for 16S rRNA), 91 fecal samples (for meta-transcriptomics). 735 patients (with 2,205 serum samples) for uric acid analysis. Cell Models: HCT116 (colorectal cancer). Animal Model: BALB/cA-nu (xenograft), C57BL/6J (orthotopic). |
|  | C2. Age | Humans: Not specified (mean/median). |
|  | C3. Sex | Humans: Not specified. Cell Model: HCT116 (male). Animal Model: BALB/cA-nu (female, 5 weeks), C57BL/6J (male, 6 weeks). |
|  | C4. Cancer type | Humans: Locally advanced rectal adenocarcinoma (LARC). Cell Model: Colorectal cancer (HCT116, MC38). |
|  | C5. Tumor stage | LARC. |
|  | C6. Performance status | Not specified. |
|  | C7. Relevant comorbidities | Not specified (exclusion of antibiotic, probiotic, proton pump inhibitor (PPI) use in the 2 weeks prior to collection). |
|  | C8. Previous treatments | Not specified (treatment-naive for neoadjuvant chemoradiotherapy). |
| D. Chemotherapy Protocol | D1. Chemotherapy regimen | Capecitabine + Oxaliplatin (CapeOx) or Capecitabine alone (concurrent chemotherapy). |
|  | D2. Treatment line | Neoadjuvant. |
|  | D3. Treatment intent | Neoadjuvant. |
|  | D4. Number of cycles | Radiotherapy (IMRT - intensity-modulated radiation therapy) 50-50.6 Gy and 41.8-45 Gy in 22-25 fractions. |
|  | D5. Combination therapies | Chemoradiotherapy (IMRT) + concurrent chemotherapy. |
|  | D6. Dose modifications | Not specified. |
| E. Microbiome Analysis | E1. Sample type | Feces (humans), serum (humans), tumor tissues (humans), bacterial culture supernatant (*Bacteroides vulgatus*). |
|  | E2. Timing of collection | Humans (feces): 2 weeks to 1 day before nCRT (pre-nCRT), 10 to 15 days after initiation (Post1), 3 days before/after radiotherapy (Post2), 6 to 8 weeks after completion (Post3). Humans (serum): Pre-nCRT, Post2, Post3. |
|  | E3. Sequencing technique | 16S rRNA sequencing (V3-V4 regions), Metatranscriptomics. |
|  | E4. Sequencing platform | MiSeq (16S rRNA), HiSeq (metatranscriptomics). |
|  | E5. Sequencing depth | 16S rRNA: 31,133,815 V3-V4 sequences (range 29,271–377,077 per sample). Metatranscriptomics: 3,600,812,066 150-bp reads (range 20,546,656-59,570,764 per sample). |
|  | E6. Bioinformatic pipeline | QIIME 2-2019.7 (for 16S rRNA), KneadDataV0.7.2, HUMAnN 2.0 (for meta-transcriptomics). LEfSe, NetShift. For metabolites: XCMS, OPLS-DA, MIMOSA. |
|  | E7. Reference database | Greengenes (version 13.8 for 16S rRNA). NCBI NR database (for metatranscriptomic genes). |
|  | E8. Quality control | Filtering of low-quality reads and human DNA (KneadDataV0.7.2, hg38). |
| F. Predictive Model | F1. Model type | Not applicable directly (the study focuses on elucidating mechanisms and identifying biomarkers). |
|  | F2. Algorithm(s) used | Gene Set Enrichment Analysis (GSEA). |
|  | F3. Predictive variables | Expression of DNA repair-related genes (HPRT1, APRT), nucleoside transporters (SLC29A1, SLC35B3), levels of nucleotide biosynthesis-related metabolites (hypoxanthine, uridine, guanosine, adenosine, thymidine), serum uric acid levels. |
|  | F4. Feature selection method | Meta-transcriptomic analysis, metabolites, gene expression. |
|  | F5. Model validation | Serum uric acid validation in an independent dataset (184 rectal cancer patients). |
|  | F6. Performance metrics | Area Under the Curve (AUC) = 0.732 (for uric acid in response prediction). |
|  | F7. Training set size | Not applicable. |
|  | F8. Test set size | 184 patients for uric acid validation. |
|  | F9. Model interpretability | Nucleotide biosynthesis pathways, DNA repair genes. |
|  | F10. Model availability | Not applicable. |
| G. Outcomes Assessed | G1. Primary study outcome | Response to nCRT (responders vs. non-responders). |
|  | G2. Response criteria | Tumor Regression Grade (TRG) 0-1 (responders), TRG 2-3 (non-responders) according to NCCN (National Comprehensive Cancer Network) TRG. Distant metastasis within 2 months after nCRT (non-responders). |
|  | G3. Evaluation time | After treatment completion (2 months). |
|  | G4. Secondary outcomes | Cancer cell survival (*in vitro*), tumor volume (*in vivo*), gH2AX levels (gamma-H2AX). |
|  | G5. Associated clinical/laboratory biomarkers | Serum uric acid, DNA repair gene expression, nucleoside transporters. |
|  | G6. Associated lifestyle/medication factors | Not specified (exclusion of antibiotics, probiotics, PPI). |
|  | G7. Associated host genetic factors | Not specified. |
| H. Key Results and Findings | H1. Key microbial taxa/species | *Bacteroides vulgatus* (BV), *Alistipes*, *Bacteroides*, *Parabacteroides*, *Actinomyces*, *Coprococcus*, *Dorea*. |
|  | H2. Direction of association | Microbial diversity: Reduced after nCRT (Shannon and Inverse Simpson). Taxa pre-nCRT vs. post-nCRT: Bacteroidetes enriched pre-nCRT; Firmicutes enriched post-nCRT. *Alistipes*, *Bacteroides*, *Parabacteroides* significantly abundant pre-nCRT; *Actinomyces*, *Coprococcus*, *Dorea* enriched post-nCRT. Taxa in responders vs. non-responders: *Bacteroides coprophilus* correlated with disease improvement in ulcerative colitis. *Bacteroides vulgatus* enriched in non-responders post-nCRT. *Bacteroides ovatus*, *Bacteroides thetaiotaomicron*, *Bacteroides xylanisolvens* modulated by *Bacteroides* species. |
|  | H3. Model predictive performance | Serum uric acid predicted chemoradiotherapy response with AUC = 0.732. |
|  | H4. Microbial diversity findings | Significant reduction in fecal microbial diversity (Shannon and Inverse Simpson) after nCRT (P < 0.001). Bray-Curtis distance significantly lower in post-nCRT samples. |
|  | H5. Microbial functional/metabolic findings | Metatranscriptomics: Nucleotide biosynthesis pathways (purine, pyrimidine) enriched in non-responders. *purF* (purine) and pyrimidine genes elevated in non-responders. Metabolomics: Elevated abundance of nucleotide biosynthesis-related metabolites (hypoxanthine, uridine, guanosine, adenosine, thymidine) in non-responders. *B. vulgatus* predominated in the nucleotide biosynthesis pathway. Serum uric acid: Increased in non-responders post-nCRT (P = 0.0016). |
|  | H6. Preclinical/*in vitro* findings | Exogenous nucleoside supplementation significantly increased HCT116 cell survival to 5-FU and irradiation in an *in vitro* model. *B. vulgatus* gavage attenuated 5-FU effect in orthotopic colorectal tumors in mice. |
|  | H7. Highlight of Findings: Unique Contribution | This longitudinal and multi-omic study uncovers a crucial mechanism of resistance to neoadjuvant chemoradiotherapy (nCRT) in rectal cancer: gut microbiome-mediated nucleotide biosynthesis, primarily by *Bacteroides vulgatus*. The "major discovery" is that the microbiota can supply exogenous nucleosides that fuel tumor cell DNA repair, protecting them from treatment. Furthermore, serum uric acid emerges as a promising prognostic biomarker. This reveals the complex "crosstalk" between the microbiome and cancer cells, paving the way for therapeutic strategies aimed at modulating this interaction to overcome treatment resistance. |
|  | H8. Validation status (internal/external) | Functional validation *in vitro* (HCT116 cells) and *in vivo* (xenografts, orthotopic MC38 model). Validation of serum uric acid biomarker in independent cohort. |
| I. Discussion/Article Implications | I1. Authors' interpretation | The heterogeneity of LARC response to nCRT can be explained by gut microbiota-mediated nucleotide biosynthesis. The regulation of nucleotide metabolism is a conserved and convergent mechanism of radiation response. |
|  | I2. Proposed biological mechanisms | *B. vulgatus* is the main species in nucleotide biosynthesis. Exogenous nucleosides protect cancer cells from 5-FU and irradiation, increasing DNA repair capacity. DNA repair genes and nucleoside transporters (SLC29A1, SLC35B3) are upregulated in non-responding tumors. Uric acid, a purine biosynthesis metabolite, functions as an antioxidant, protecting tumor cells from oxidative stress. |
|  | I3. Correlations with other biomarkers | Serum uric acid is a potential prognostic biomarker. |
|  | I4. Impact of interventions (if applicable) | Pyrimidine or purine biosynthesis inhibitors (brequinar, mycophenolic acid) decrease cancer cell survival. Nucleoside transporter inhibitor (NBMPR) attenuates the protective effect of nucleosides. |
|  | I5. Consistency/Inconsistency of findings | Finding of *B. vulgatus* enriched in non-responders is consistent with literature that associates it with ulcerative colitis pathogenesis and progression from adenoma to carcinoma. |
|  | I6. Transferability/Generalization | The hypothesis that microbiota-mediated nucleotide biosynthesis influences chemoradiosensitivity is applicable to various cancer types. |
|  | I7. Causality vs. Association | The study demonstrates causality through *in vitro* and *in vivo* functional validations (nucleoside supplementation, *B. vulgatus* gavage). |
| J. Clinical Application Potential and Barriers | J1. Potential for implementation in practice | Actions aimed at interrupting nucleoside supply by microbiota or nucleoside entry into tumor cells can be promising therapeutic strategies to overcome chemoradiotherapy resistance. Monitoring serum uric acid can help predict prognosis. |
|  | J2. Advantages | Identification of resistance mechanisms and prognostic biomarkers. |
|  | J3. Barriers to implementation | The causal relationship between bacterial nucleosides and tumor response still needs to be defined. Need for large sample validations for uric acid. |
|  | J4. Cost-effectiveness | Not discussed. |
|  | J5. Recommendations for future research | Large-scale validation and elucidation of specific mechanisms. |
| K. Limitations and Bias of the Original Study | K1. Limitations declared by authors | Microbiome not assessed at strain level, which is relevant for *Bacteroides*. Nucleoside concentration used *in vitro* may not be fully representative of the tumor microenvironment. Uric acid's mechanism of action needs to be defined. |
|  | K2. Selection bias | Not explicitly stated. |
|  | K3. Confounding bias | Not explicitly stated. |
|  | K4. Generalization | Findings may be generalizable to other cancer types treated with chemoradiotherapy. |
|  | K5. Reproducibility | Metatranscriptomic and 16S rRNA data available in the Genome Sequence Archive (HRA001813). Clinical and transcriptomic data from the TCGA READ cohort. |

Table S13. Article 13: AENEAS: A Randomized Phase III Trial of Aumolertinib Versus Gefitinib as First-Line Therapy for Locally Advanced or Metastatic Non–Small-Cell Lung Cancer With EGFR Exon 19 Deletion or L858R Mutations

| Category | Extracted Variables | Detailing |
| --- | --- | --- |
| A. Study Identification | A1. Lead Author (Year) | Lu S (2022) |
|  | A2. Title | AENEAS: A Randomized Phase III Trial of Aumolertinib Versus Gefitinib as First-Line Therapy for Locally Advanced or Metastatic Non–Small-Cell Lung Cancer With EGFR Exon 19 Deletion or L858R Mutations |
|  | A3. Journal | Journal of Clinical Oncology. |
|  | A4. Country/Region | China (53 centers in mainland China) |
|  | A5. Funding | Hansoh Pharmaceutical Group Co, Ltd. Type: Industrial. |
|  | A6. Conflicts of interest | Yes. Several authors declare financial relationships (consultancy, research, honoraria, employment) with Hansoh Pharmaceutical Group Co, Ltd., AstraZeneca, Pfizer, Boehringer Ingelheim, BMS (Bristol Myers Squibb), Merck, etc. |
| B. Methodological Design | B1. Study type | Randomized, double-blind, Phase III clinical trial. |
|  | B2. Follow-up duration | Median of 20.5 months (aumolertinib) and 20.7 months (gefitinib) at the data cutoff of January 15, 2021. |
|  | B3. Study center(s) | Multicenter (53 study sites in mainland China). |
|  | B4. Recruitment period | November 30, 2018 to September 6, 2019. |
|  | B5. Ethical approval | The protocol was approved by the ethics committee of each institution. Registration: ClinicalTrials.gov (NCT03849768). |
| C. Study Population | C1. Sample size | 429 patients randomized (214 aumolertinib, 215 gefitinib). |
|  | C2. Age | Median 59 years (aumolertinib) and 62 years (gefitinib). Range: 25-81 years. |
|  | C3. Sex | Male: 37.4% (aumolertinib), 37.2% (gefitinib). Female: 62.6% (aumolertinib), 62.8% (gefitinib). |
|  | C4. Cancer type | Locally advanced or metastatic Non–Small-Cell Lung Cancer (NSCLC) with activating Epidermal Growth Factor Receptor (EGFR) mutations (exon 19 deletion or L858R). |
|  | C5. Tumor stage | IIIB (5.6% aumolertinib, 7.9% gefitinib), IV (94.4% aumolertinib, 92.1% gefitinib). |
|  | C6. Performance status | Eastern Cooperative Oncology Group (ECOG) PS 0 (23.8% aumolertinib, 25.1% gefitinib), ECOG PS 1 (74.8% aumolertinib, 74.0% gefitinib). |
|  | C7. Relevant comorbidities | Not specified. |
|  | C8. Previous treatments | No prior systemic therapy allowed (except in adjuvant/neoadjuvant setting). No prior EGFR inhibitor treatment allowed. |
| D. Chemotherapy Protocol | D1. Chemotherapy regimen | Aumolertinib (110 mg) or Gefitinib (250 mg), both administered once daily, orally. |
|  | D2. Treatment line | First-line. |
|  | D3. Treatment intent | Treatment (tyrosine kinase inhibitor (TKI)). |
|  | D4. Number of cycles | Treatment was continued until disease progression, withdrawal of consent, development of unacceptable side effects, or fulfillment of other discontinuation criteria. |
|  | D5. Combination therapies | Not applicable (monotherapy). |
|  | D6. Dose modifications | Not specified, but dose interruptions were implemented in 16.8% (aumolertinib) and 24.7% (gefitinib). Dose reduction in 4.2% (aumolertinib) and 4.7% (gefitinib). |
| E. Microbiome Analysis | E1. Sample type | Not applicable (Phase III clinical trial for drug evaluation, does not include microbiome analysis). |
|  | E2. Timing of collection | Not applicable. |
|  | E3. Sequencing technique | Not applicable. |
|  | E4. Sequencing platform | Not applicable. |
|  | E5. Sequencing depth | Not applicable. |
|  | E6. Bioinformatic pipeline | Not applicable. |
|  | E7. Reference database | Not applicable. |
|  | E8. Quality control | Not applicable. |
| F. Predictive Model | F1. Model type | Not applicable (Phase III clinical trial evaluating drugs, not microbiome-based predictive models). |
|  | F2. Algorithm(s) used | Not applicable. |
|  | F3. Predictive variables | Not applicable. |
|  | F4. Feature selection method | Not applicable. |
|  | F5. Model validation | Not applicable. |
|  | F6. Performance metrics | Not applicable. |
|  | F7. Training set size | Not applicable. |
|  | F8. Test set size | Not applicable. |
|  | F9. Model interpretability | Not applicable. |
|  | F10. Model availability | Not applicable. |
| G. Outcomes Assessed | G1. Primary study outcome | Progression-Free Survival (PFS) (investigator-assessed). |
|  | G2. Response criteria | RECIST 1.1 (Response Evaluation Criteria in Solid Tumors). |
|  | G3. Evaluation time | Imaging performed at baseline and every 6 weeks (67 days) up to 15 months, then every 12 weeks. |
|  | G4. Secondary outcomes | Overall Survival (OS), Objective Response Rate (ORR), Duration of Response (DoR), Disease Control Rate (DCR), Depth of Response. Adverse Events (AEs). |
|  | G5. Associated clinical/laboratory biomarkers | EGFR mutations (ex19del, L858R). Central Nervous System (CNS) metastases. Creatine Phosphokinase (CPK) levels. |
|  | G6. Associated lifestyle/medication factors | Smoking history. |
|  | G7. Associated host genetic factors | EGFR mutations (exon 19 deletion or L858R). |
| H. Key Results and Findings | H1. Key microbial taxa/species | Not applicable (the study does not include microbiome analysis). |
|  | H2. Direction of association | Not applicable. |
|  | H3. Model predictive performance | Not applicable. |
|  | H4. Microbial diversity findings | Not applicable. |
|  | H5. Microbial functional/metabolic findings | Not applicable. |
|  | H6. Preclinical/*in vitro* findings | Not applicable. |
|  | H7. Highlight of Findings: Unique Contribution | This article reports a Phase III clinical trial for a new drug, aumolertinib, for NSCLC, and does not address the clinical application potential of microbiome-based predictive models. The main contribution is the demonstration that aumolertinib is a well-tolerated and effective third-generation tyrosine kinase inhibitor (TKI) for EGFR-mutated NSCLC as a first-line treatment. |
|  | H8. Validation status (internal/external) | Not applicable (the study does not include microbiome analysis). |
| I. Discussion/Article Implications | I1. Authors' interpretation | Aumolertinib is effective and well-tolerated, offering significant PFS benefit compared to gefitinib in EGFR-mutated NSCLC patients. It is an important alternative to diversify first-line treatment options. |
|  | I2. Proposed biological mechanisms | Aumolertinib is a third-generation EGFR TKI that demonstrates greater selectivity against sensitizing and T790M EGFR mutations, with less inhibition of wild-type EGFR. |
|  | I3. Correlations with other biomarkers | EGFR mutations (ex19del, L858R) and presence of CNS (Central Nervous System) metastases are stratification factors and predictors of benefit. |
|  | I4. Impact of interventions (if applicable) | Not applicable. |
|  | I5. Consistency/Inconsistency of findings | Superior efficacy in exon 19 deletion mutations compared to L858R is consistent with other third-generation TKIs. |
|  | I6. Transferability/Generalization | The findings, although in Chinese patients, are broadly applicable globally due to consistency in the approach to EGFR-mutated NSCLC. |
|  | I7. Causality vs. Association | Not applicable (Phase III clinical trial, not microbiome analysis). |
| J. Clinical Application Potential and Barriers | J1. Potential for implementation in practice | Aumolertinib as a new first-line treatment option for EGFR-mutated NSCLC. |
|  | J2. Advantages | PFS significantly longer. Better tolerability profile (lower rates of rash and diarrhea) compared to gefitinib. |
|  | J3. Barriers to implementation | Not applicable (the study does not include microbiome analysis). |
|  | J4. Cost-effectiveness | Not discussed. |
|  | J5. Recommendations for future research | Ongoing studies in the adjuvant setting and in combination with chemotherapy. OS and crossover analyses will be reported in the future. |
| K. Limitations and Bias of the Original Study | K1. Limitations declared by authors | Study conducted exclusively in China with Chinese patients (extrapolation is supported by global consistencies, but it is a geographical limitation). Low OS maturity at the time of analysis. |
|  | K2. Selection bias | Not applicable. |
|  | K3. Confounding bias | Not applicable. |
|  | K4. Generalization | Although authors state results are broadly applicable, study was conducted only in China. |
|  | K5. Reproducibility | Not applicable. |

Table S14. Article 14: Fecal microbiota transplant overcomes resistance to anti–PD-1 therapy in melanoma patients

| Category | Extracted Variables | Detailing |
| --- | --- | --- |
| A. Study Identification | A1. Lead Author (Year) | Davar D (2021) |
|  | A2. Title | Fecal microbiota transplant overcomes resistance to anti–PD-1 therapy in melanoma patients |
|  | A3. Journal | Science. |
|  | A4. Country/Region | USA (Pittsburgh, Bethesda, Frederick, Corvallis) |
|  | A5. Funding | Merck MSD (research contract for D.D.), Melanoma Breakthrough Foundation Breakthrough Consortium (D.D.), National Cancer Institute (NCI) of NIH (National Institutes of Health) (R01 CA228181 and R01 CA222203 for H.M.Z.), James W. and Frances G. McGlothlin Chair in Melanoma Immunotherapy Research (H.M.Z.), NCI Comprehensive Cancer Center Support CORE grant (P30 CA047904), Intramural Research Program of NIH, NIAID, NCI Center for Cancer Research. Type: Industrial and Public. |
|  | A6. Conflicts of interest | Yes. D.D. (research and consultancy for various companies), Y.G.N. (research support), J.M.K. (research support and consultancy), H.M.Z. (research support and consultancy), H.M.Z., D.D., J.A.M., R.R.R., G.T. and A.K.D. are patent inventors. |
| B. Methodological Design | B1. Study type | Phase II, single-arm clinical trial. |
|  | B2. Follow-up duration | Median of 7 months of follow-up. |
|  | B3. Study center(s) | Unicenter (UPMC Hillman Cancer Center, University of Pittsburgh). |
|  | B4. Recruitment period | June 2018 to January 2020. |
|  | B5. Ethical approval | Not specified in the article, but conducted within the context of a clinical trial (NCT03341143). |
| C. Study Population | C1. Sample size | 16 patients recruited, 15 patients received Fecal Microbiota Transplant (FMT) and pembrolizumab and were evaluable for response. 7 FMT donors. |
|  | C2. Age | Not specified (mean/median). |
|  | C3. Sex | Not specified. |
|  | C4. Cancer type | Advanced metastatic melanoma. |
|  | C5. Tumor stage | Advanced metastatic. |
|  | C6. Performance status | Not specified. |
|  | C7. Relevant comorbidities | Exclusion due to active infections. |
|  | C8. Previous treatments | Primarily refractory to anti-PD-1 therapy (defined as no prior response to anti-PD-1 alone or in combination with anti-CTLA-4 or investigational agents). |
| D. Chemotherapy Protocol | D1. Chemotherapy regimen | Pembrolizumab (anti-PD-1). |
|  | D2. Treatment line | After previous anti-PD-1 failure (refractory patients). |
|  | D3. Treatment intent | Immunotherapy (overcoming anti-PD-1 resistance). |
|  | D4. Number of cycles | Pembrolizumab every 3 weeks until disease progression or intolerable toxicity. |
|  | D5. Combination therapies | Immunotherapy (pembrolizumab) + Fecal Microbiota Transplant (FMT). |
|  | D6. Dose modifications | Not specified. |
| E. Microbiome Analysis | E1. Sample type | Feces (recipients and donors), serum (recipients). |
|  | E2. Timing of collection | Recipients: Pre-FMT (7 to 21 days before), post-FMT (weekly for 12 weeks, then every 3 weeks). Donors: Serial samples. |
|  | E3. Sequencing technique | Shotgun metagenomics. |
|  | E4. Sequencing platform | Not specified. |
|  | E5. Sequencing depth | Not specified. |
|  | E6. Bioinformatic pipeline | UMAP (Uniform Manifold Approximation and Projection) for composition analysis. Meta-analysis using Fisher's method. |
|  | E7. Reference database | Not specified. |
|  | E8. Quality control | Not specified (mentions "extensive infectious studies" for donors). |
| F. Predictive Model | F1. Model type | Not applicable (the study focuses on evaluating an intervention, not on developing a predictive model for clinical practice). |
|  | F2. Algorithm(s) used | Not applicable. |
|  | F3. Predictive variables | Microbial taxa (e.g., Firmicutes (Lachnospiraceae, Ruminococcaceae), Actinobacteria (Bifidobacteriaceae, Coriobacteriaceae), Bacteroidetes). |
|  | F4. Feature selection method | Statistical analysis to identify differentially abundant taxa. |
|  | F5. Model validation | Not applicable. |
|  | F6. Performance metrics | Not applicable. |
|  | F7. Training set size | Not applicable. |
|  | F8. Test set size | Not applicable. |
|  | F9. Model interpretability | Cladograms, transkingdom networks, abundance plots. |
|  | F10. Model availability | Not applicable. |
| G. Outcomes Assessed | G1. Primary study outcome | Safety and efficacy of FMT + anti-PD-1 (Objective Response (OR) and Stable Disease (SD) >12 months). |
|  | G2. Response criteria | RECIST 1.1 (Response Evaluation Criteria in Solid Tumors). |
|  | G3. Evaluation time | Every 12 weeks for radiographic evaluation. |
|  | G4. Secondary outcomes | Progression-Free Survival (PFS), Overall Survival (OS), Adverse Events (AEs), gut microbiota perturbation, FMT engraftment, CD8+ T cell activation, Natural Killer (NK) cell activation. |
|  | G5. Associated clinical/laboratory biomarkers | CD56+CD8+ T cells, TIGIT, CD57, 2B4, OX40, ICOS, 4-1BB, CD16, NKp46, NKp30, granzyme B, perforin, CD103, T-bet, LAG-3, CD27 (CD8+ T cells). CXCL8 (IL-8) and SPP1 (osteopontin) in myeloid cells. Serum bile acids, metabolomics and lipidomics, circulating cytokines and chemokines (CCL2, CXCL8, IL-18, IL-12p70, IFN-γ, IL-21, CXCL13, IL-5, IL-13, IL-10, TNF, TRAIL, CX3CL1, FLT3L). |
|  | G6. Associated lifestyle/medication factors | Antibiotic use (prohibited during the study, but impact evaluated in case of use), diet. |
|  | G7. Associated host genetic factors | Not specified. |
| H. Key Results and Findings | H1. Key microbial taxa/species | Enriched in Responders (R): Firmicutes (Lachnospiraceae and Ruminococcaceae), Actinobacteria (Bifidobacteriaceae and Coriobacteriaceae), *Bifidobacterium longum*, *Collinsella aerofaciens*, *Faecalibacterium prausnitzii*. Decreased in R: Bacteroidetes. |
|  | H2. Direction of association | FMT induced rapid and durable microbiome perturbation. R showed a significant shift towards donor microbiome (P = 0.006) and greater intra-patient microbiome variance. FMT engraftment and humoral immune response (IgG) against donor bacteria associated with response. |
|  | H3. Model predictive performance | Not applicable. |
|  | H4. Microbial diversity findings | No significant difference in alpha diversity between donors and pre-FMT recipients. CR donor microbiota had higher alpha diversity than PR. |
|  | H5. Microbial functional/metabolic findings | Increased serum bile acids (primary to secondary transformation) in R. Increased bacterial catabolism products of aromatic compounds (e.g., hippurate, p-cresol sulfate, hydrocinnamate) in R. Significant alterations in serum metabolomic and lipidomic profile in R. |
|  | H6. Preclinical/*in vitro* findings | Not applicable. |
|  | H7. Highlight of Findings: Unique Contribution | This pioneering clinical trial demonstrates that Fecal Microbiota Transplant (FMT) from responder donors, in combination with anti-PD-1, can overcome primary immunotherapy resistance in advanced melanoma patients. The "major discovery" is evidence that FMT reprograms the tumor microenvironment and modulates systemic immune response, leading to favorable clinical and immunological responses. Successful donor microbiome engraftment, accompanied by a specific IgG response, is crucial for intervention success, pointing to the microbiome's potential as an active therapy to enhance immunotherapy. |
|  | H8. Validation status (internal/external) | Validation of intervention efficacy in a Phase II clinical trial. |
| I. Discussion/Article Implications | I1. Authors' interpretation | A single FMT with anti-PD-1 can overcome primary anti-PD-1 resistance in a subset of advanced melanoma patients, reprogramming the tumor microenvironment. FMT engraftment and the recipient's specific IgG response to the donor microbiome are critical. |
|  | I2. Proposed biological mechanisms | Peripheral immunity: Increased activated CD56+CD8+ T cells, greater activation of CD8+ T cells and mucosal-associated invariant T (MAIT) cells in responders. Intratumoral immunity: Counteracting myeloid-mediated immunosuppression, increased CD8+ T cell activation. CXCL8 (IL-8) and SPP1 (osteopontin) expressed in myeloid cells can be regulated by FMT. |
|  | I3. Correlations with other biomarkers | Increased CD8+ T cell activation, decreased frequency of IL-8 producing myeloid cells in tumors. Circulating cytokine/chemokine markers (e.g., CCL2, CXCL8, IL-18 decreased; IL-12p70, IFN-γ, IL-21, CXCL13, IL-5, IL-13, IL-10, TNF, TRAIL, CX3CL1, FLT3L increased). |
|  | I4. Impact of interventions (if applicable) | FMT is an effective intervention. Antibiotic use rapidly perturbs FMT-induced microbiome changes, leading to clinical progression. |
|  | I5. Consistency/Inconsistency of findings | Taxa enriched in responders (Lachnospiraceae, Ruminococcaceae, Bifidobacteriaceae, Coriobacteriaceae) and decreased (Bacteroidetes) are consistent with previous reports. |
|  | I6. Transferability/Generalization | Findings are specific to PD-1 refractory melanoma patients. |
|  | I7. Causality vs. Association | The study demonstrates causality of intervention (FMT) in overcoming resistance and immune modulation. |
| J. Clinical Application Potential and Barriers | J1. Potential for implementation in practice | FMT as a therapy to overcome immunotherapy resistance in melanoma patients. |
|  | J2. Advantages | Well-tolerated therapy, with potential to induce durable responses in refractory patients. |
|  | J3. Barriers to implementation | FMT engraftment was heterogeneous in non-responders. Clinical response was not universal. Need for more studies to identify biomarkers for patient selection and donor safety. |
|  | J4. Cost-effectiveness | Not discussed. |
|  | J5. Recommendations for future research | Validation in larger randomized clinical trials. Identification of microbial, circulating, and intratumoral biomarkers to select patients most likely to benefit. Identification of a bacterial consortium capable of converting refractory patients into responders. |
| K. Limitations and Bias of the Original Study | K1. Limitations declared by authors | Small sample size (n=15 evaluable). Lack of sufficient tumor tissue to assess tumor mutational burden, PD-L1 expression, and IFN-γ gene signature. |
|  | K2. Selection bias | Not applicable (single-arm Phase II trial). |
|  | K3. Confounding bias | Not explicitly stated. |
|  | K4. Generalization | Preliminary findings warrant future evaluation in larger randomized clinical trials. |
|  | K5. Reproducibility | Sequencing data and metadata were deposited in BioProject (PRJNA672867). |

Table S15. Article 15: Intestinal microbiota signatures of clinical response and immune-related adverse events in melanoma patients treated with anti-PD-1

| Category | Extracted Variables | Detailing |
| --- | --- | --- |
| A. Study Identification | A1. Lead Author (Year) | McCulloch JA (2022) |
|  | A2. Title | Intestinal microbiota signatures of clinical response and immune-related adverse events in melanoma patients treated with anti-PD-1 |
|  | A3. Journal | Nature Medicine. |
|  | A4. Country/Region | USA (Pittsburgh, Bethesda, Corvallis, Houston, Chicago, New York), other published cohorts. |
|  | A5. Funding | Intramural Research Program of NIH (National Institutes of Health), NCI (National Cancer Institute), Center for Cancer Research. Melanoma Breakthrough Foundation Breakthrough Consortium (D.D.). NIH/NCI (R01 CA228181 and R01 CA222203 for H.M.Z.). James W. and Frances G. McGlothlin Chair in Melanoma Immunotherapy Research (H.M.Z.). Irvington postdoctoral fellowship from the Cancer Research Institute (M.V.). University of Pittsburgh Medical Center Hillman Cancer Center (HCC) Microbiome Shared Facility and Cytometry Facility (P30 CA047904). Type: Public. |
|  | A6. Conflicts of interest | Yes. D.D. (research support and consultancy for various companies), H.M.Z. (research support and consultancy for various companies), D.D., H.M.Z., J.A.M., R.R.R., G.T. and A.K.D. are patent inventors. |
| B. Methodological Design | B1. Study type | Prospective cohort study (Pittsburgh cohort), meta-analysis, and reanalysis of four published datasets. |
|  | B2. Follow-up duration | PFS (Progression-Free Survival) and OS (Overall Survival) evaluated at 10 months to 1 year. |
|  | B3. Study center(s) | Unicenter (University of Pittsburgh’s Hillman Cancer Center for the Pittsburgh cohort), multicenter in meta-analysis. |
|  | B4. Recruitment period | Not specified for the Pittsburgh cohort. |
|  | B5. Ethical approval | Sample bank protocols approved by the Institutional Review Board (IRB) of the University of Pittsburgh (HCC 96–099 and 20–019, approval MOD19080226–004 and STUDY20010266). |
| C. Study Population | C1. Sample size | Pittsburgh cohort: 94 patients (63 P-ESC (early sample cohort), 31 P-LSC (late sample cohort)). Meta-analysis: 155 shotgun samples and 150 16S amplicon samples from 5 melanoma cohorts (total N = 185). |
|  | C2. Age | Not specified (mean/median). |
|  | C3. Sex | Not specified. |
|  | C4. Cancer type | Melanoma (Stage III or IV). |
|  | C5. Tumor stage | Stage III or IV. |
|  | C6. Performance status | Not specified. |
|  | C7. Relevant comorbidities | Not specified. |
|  | C8. Previous treatments | 83% of patients treated with anti-PD-1 as first-line. 17% received previous therapy. |
| D. Chemotherapy Protocol | D1. Chemotherapy regimen | Anti-PD-1 (nivolumab, pembrolizumab or experimental anti-PD-1). |
|  | D2. Treatment line | First-line for 83% of patients in the Pittsburgh cohort. |
|  | D3. Treatment intent | Immunotherapy (advanced melanoma treatment). |
|  | D4. Number of cycles | Not specified. |
|  | D5. Combination therapies | Anti-PD-1 alone or in combination with pegylated interferon (peg-IFN). |
|  | D6. Dose modifications | Not specified. |
| E. Microbiome Analysis | E1. Sample type | Feces. Exfoliated intestinal luminal cells ("exfoliome"). |
|  | E2. Timing of collection | Baseline (before or up to 4 months after anti-PD-1 initiation) for P-ESC. After >4 months for P-LSC. |
|  | E3. Sequencing technique | Shotgun metagenomics. 16S rRNA sequencing (for AGP - American Gut Project). Transcriptomic analysis (RNA from exfoliated intestinal cells). |
|  | E4. Sequencing platform | NovaSeq System (Illumina) (2x150 bp paired-end protocol). |
|  | E5. Sequencing depth | Mean sequencing depth of 10.14 Gbp ± 4.95 Gbp (excluding host reads). |
|  | E6. Bioinformatic pipeline | JAMS_BW (v1.5.7), Trimmomatic (v0.36), Bowtie2 (v2.3.2), MEGAHIT (v1.2.9), Kraken 2, Prokka (v1.14.6), ComBat R package (for batch correction), LEfSe (Linear Discriminant Analysis Effect Size), GSEA (Gene Set Enrichment Analysis), PhenoGraph R package. |
|  | E7. Reference database | NCBI GenBank (for Kraken 2). Immgen database (for cell GSEA). AGP (for microbiotypes). |
|  | E8. Quality control | Quality trimming, adapter clipping, alignment to human genome (for host read removal). |
| F. Predictive Model | F1. Model type | Machine Learning (ML) classifier to predict PD-1 response and irAEs. Cox risk model (for PFS). Transkingdom network. |
|  | F2. Algorithm(s) used | Random Forest (RF), Generalized Linear Model (GLM), polynomial Support Vector Machine (SVM). Cox Proportional Hazards Regression (CoxPH) for PFS. PERMANOVA for diversity. |
|  | F3. Predictive variables | Microbial taxa (genera, species, microbial genes), NLR, Body Mass Index (BMI), PPI use. |
|  | F4. Feature selection method | Fisher’s 'q value', Evaluate Cutpoints, GSEA (Gene Set Enrichment Analysis), transkingdom network analysis. |
|  | F5. Model validation | Leave-one-out cross-validation on batch-corrected datasets. |
|  | F6. Performance metrics | Receiver Operating Characteristic Area Under the Curve (ROC AUC), Hazard Ratio (HR), P-value. |
|  | F7. Training set size | Meta-analysis: 155 shotgun samples and 150 16S amplicon samples. Pittsburgh cohort: 94 patients. |
|  | F8. Test set size | Meta-analysis: 4 independent published cohorts. |
|  | F9. Model interpretability | Cladograms, transkingdom networks, heatmaps, volcano plots, t-UMAP (t-distributed uniform manifold approximation and projection). |
|  | F10. Model availability | Code for shotgun sequencing analysis on GitHub (JAMS_BW package). R GSEA code. Transkingdom network code on GitHub. |
| G. Outcomes Assessed | G1. Primary study outcome | PFS (Progression-Free Survival) and OS (Overall Survival) to anti-PD-1 therapy. irAEs (Immune-Related Adverse Events). |
|  | G2. Response criteria | RECIST 1.1 for radiographic evaluation. |
|  | G3. Evaluation time | PFS at 9-10 months. |
|  | G4. Secondary outcomes | Gut microbiome composition, microbial genes, host gene expression, NLR (neutrophil-to-lymphocyte ratio). |
|  | G5. Associated clinical/laboratory biomarkers | NLR (elevated NLR associated with worse survival), BMI, LDH (lactate dehydrogenase). |
|  | G6. Associated lifestyle/medication factors | Use of PPIs (Proton Pump Inhibitors), antibiotics. |
|  | G7. Associated host genetic factors | Not specified. |
| H. Key Results and Findings | H1. Key microbial taxa/species | Favorable (responders (NP) / better PFS): *Ruminococcus (Mediterraneibacter) torques*, *Blautia producta*, *Blautia wexlerae*, *Blautia hansenii*, *Eubacterium rectale*, *Ruminococcus (Mediterraneibacter) gnavus*, *Anaerostipes hadrus*. Members of the phylum Actinobacteria and family Lachnospiraceae. Unfavorable (progressors (P) / worse PFS): *Prevotella* spp., *Oscillibacter* spp., *Alistipes* spp., *Sutterellaceae* spp., *Bacteroides* spp., Proteobacteria phylum. Members of the phylum Bacteroidetes and Proteobacteria. *Streptococcus* spp. Associated with irAEs: *Lachnospiraceae* spp. (irAEs), *Streptococcus* spp. (irAEs). |
|  | H2. Direction of association | Baseline gut microbiome composition optimally associated with clinical outcome at ~1 year post-treatment. Gram-negative taxa enriched in P. Lachnospiraceae spp. and Actinobacteria associated with better response. *Streptococcus* spp. associated with irAEs and worse PFS. PPI use correlated with *Streptococcus* spp. and oralization of the microbiome. |
|  | H3. Model predictive performance | ML models (Random Forest, GLM, SVM): AUC ranging from 0.21 to 0.79 for cross-cohort validation. Leave-one-out cross-validation with batch-corrected data: AUCs between 0.54 and 1.00 (significant in 12 of 15 combinations). |
|  | H4. Microbial diversity findings | No significant differences in alpha diversity (Inverse Simpson, Shannon, taxon richness) between R and NR for shotgun or 16S amplicon in meta-analysis. Diversity is a weak predictor in individual studies and heterogeneous across studies. |
|  | H5. Microbial functional/metabolic findings | Microbial genes: LPS synthesis in P, iron bioavailability in NP. Genes for polysaccharide deacetylation (favorable), flavin/riboflavin metabolism (favorable). Genes for LPS synthesis and mucus degradation (alpha-l-fucosidase, alpha-galactosidase) (unfavorable). |
|  | H6. Preclinical/*in vitro* findings | Not applicable (human study). |
|  | H7. Highlight of Findings: Unique Contribution | This study highlights the importance of baseline gut microbiome composition in predicting clinical response and Immune-Related Adverse Events (irAEs) to anti-PD-1 therapy in melanoma. The "major discovery" is the identification that Gram-negative taxa are associated with a systemic and intestinal inflammatory state, driving poor response, while specific microbiotypes, some with geographical distribution, may explain heterogeneity across cohorts. This points to the need to consider geographical context and the microbiome as a causal factor in immunosuppression, offering a roadmap for developing biomarkers and targeted interventions to optimize immunotherapy. |
|  | H8. Validation status (internal/external) | *In silico* validation in independent cohort (Houston) for some findings. Meta-analysis of 5 melanoma cohorts. |
| I. Discussion/Article Implications | I1. Authors' interpretation | Baseline microbiome composition is strongly associated with clinical outcome at approximately 1 year after treatment initiation, suggesting that microbiome influence becomes dominant during this period. Unfavorable microbiomes (rich in Gram-negative bacteria) promote systemic and intestinal inflammation. |
|  | I2. Proposed biological mechanisms | Unfavorable microbes enriched in Gram-negative bacteria can promote an LPS (lipopolysaccharide)-dominated inflammatory signature in the gut, leading to systemic inflammation (elevated NLR) and poor response to anti-PD-1. Microbial genes related to polysaccharide deacetylation (favorable) and mucus degradation/LPS synthesis (unfavorable) influence response. |
|  | I3. Correlations with other biomarkers | High NLR (neutrophil-to-lymphocyte ratio) correlates with worse survival and enrichment of Gram-negative bacteria. PPI use correlates with microbiota oralization (*Streptococcus* spp., *Veillonella* spp.). |
|  | I4. Impact of interventions (if applicable) | Does not discuss interventions. |
|  | I5. Consistency/Inconsistency of findings | Lack of concordance among studies may be explained by: heterogeneity of response over time, methodological differences, microbiotypes with non-uniform geographical distribution. |
|  | I6. Transferability/Generalization | Findings were confirmed in meta-analyses of multiple cohorts, suggesting robustness. However, uneven geographical distribution of microbiotypes suggests that biomarkers may be region-specific. |
|  | I7. Causality vs. Association | Transkingdom network analysis suggests regulatory relationships between host and microbes. |
| J. Clinical Application Potential and Barriers | J1. Potential for implementation in practice | Development of microbial biomarkers to predict clinical response and irAEs to anti-PD-1 therapy, aiding patient stratification. |
|  | J2. Advantages | Potential to predict timing of response and identify patients at risk of irAEs. |
|  | J3. Barriers to implementation | Heterogeneity of microbiome and response. Need for more validation in large prospective cohorts. |
|  | J4. Cost-effectiveness | Not discussed. |
|  | J5. Recommendations for future research | Studies to confirm relevance of findings in large prospective cohorts. Preclinical studies to deepen understanding of mechanisms. |
| K. Limitations and Bias of the Original Study | K1. Limitations declared by authors | Inconsistencies among published microbial signatures. Meta-diversity alpha analysis did not show significant differences. Microbiome analysis of P-LSC did not predict late therapeutic failure. |
|  | K2. Selection bias | Not explicitly stated. |
|  | K3. Confounding bias | Heterogeneity among studies (collection/extraction protocols, geographical influences, diet and lifestyle). |
|  | K4. Generalization | Inconsistencies across cohorts may be explained by "microbiotypes" with non-uniform geographical distribution. |
|  | K5. Reproducibility | Data available in relevant repositories (BioProject accession no. PRJNA762360; AGP: ENA database PRJEB11419; other accesses via BioProject). |

Table S16. Article 16: Gut OncoMicrobiome Signatures (GOMS) as next-generation biomarkers for cancer immunotherapy

| Category | Extracted Variables | Detailing |
| --- | --- | --- |
| A. Study Identification | A1. Lead Author (Year) | Thomas AM (2023) |
|  | A2. Title | Gut OncoMicrobiome Signatures (GOMS) as next-generation biomarkers for cancer immunotherapy |
|  | A3. Journal | Nature Reviews Clinical Oncology. |
|  | A4. Country/Region | Italy, France, Canada, USA (in meta-analysis) |
|  | A5. Funding | Seerave Foundation (M.F.), CIHR (Canadian Institute for Health Research), FRQS (Fonds de la Recherche Québec-Santé), Terry Fox Marathon of Hope Program and Weston Foundation (B.R.). SU2C-AACR-IRG-19-17, Melanoma Moon Shots Program, Melanoma Research Alliance (4022024) and NIH (1 R01 CA219896-01A1) (J.A.W.). European Union Horizon 2020 (ONCOBIOME-825410, MASTER-818368, IHMCSA-964590), ERC-STG MetaPG-716575, ERC-CoG microTOUCH-101045015, NCI NIH (1U01CA230551) (N.S.). ANR (Agence Nationale de la Recherche) (French-German Ileobiome 19-CE15-0029-01), European Union Horizon Europe (PREVALUNG-EU), RHU5 “ANR-21-RHUS-0017” IMMUNOLIFE, SIGN’IT ARC foundation and SIRIC Stratified Oncology Cell DNA Repair and Tumour Immune Elimination (SOCRATE) (L.Z.). Elior, Seerave Foundation, ANR, Badinter Philantropia, Cancéopole Ile-de-France; Dassault, FHU CARE, FRM (Fondation pour la Recherche Médicale), Inserm (HTE), INCa (Institut National du Cancer), Institut Universitaire de France, LabEx Immuno-Oncology and Ligue contre le Cancer (Equipe labelisée) (L.Z. and G.K.). Type: Industrial and Public. |
|  | A6. Conflicts of interest | Yes. A.M.T. (employee of Microbiotica), B.R. (grants, pending patent), G.K. (research contracts, board of directors, scientific co-founder), J.A.W. (patent inventor, lecturing/honoraria compensation, consultant/board member, stock options), L.Z. (scientific co-founder, scientific board chairman, research grants, former board member, consultant), N.S. (no conflict). |
| B. Methodological Design | B1. Study type | Systematic Review, Meta-analysis, and Mega-analysis of shotgun metagenomic data. |
|  | B2. Follow-up duration | Not applicable (review of various studies). |
|  | B3. Study center(s) | Not applicable (review). |
|  | B4. Recruitment period | Not applicable (review). |
|  | B5. Ethical approval | Not applicable (review). |
| C. Study Population | C1. Sample size | Mega-analysis: 1,879 adult cancer patients (8 cancer types), 5,341 non-cancer individuals. ICI Meta-analysis: 808 cancer patients (12 cohorts, 8 studies) for ORR (Objective Response Rate) analysis. |
|  | C2. Age | Not specified (review of various studies). |
|  | C3. Sex | Not specified (review of various studies). |
|  | C4. Cancer type | Colorectal Cancer (CRC), Lung (NSCLC), Breast, Ovarian, Prostate, Melanoma, Chronic Leukemia, Esophageal Squamous Cell Carcinoma (ESCC), Urothelial Carcinoma. Pancreatic ductal carcinoma (PDAC). |
|  | C5. Tumor stage | Advanced stage (melanoma), Stages II–III (breast cancer), Stages I–II (PDAC), Stage III–IV (MIBC). |
|  | C6. Performance status | Not specified. |
|  | C7. Relevant comorbidities | Aging, cachexia, obesity, pulmonary and hepatic dysfunction, inflammatory bowel disease (IBD), metabolic syndrome, autoimmune diseases, mental health disorders. |
|  | C8. Previous treatments | Antibiotics, proton pump inhibitors (PPIs), anti-inflammatory agents, osmotic laxatives, biguanides, polychemotherapy, endocrine therapy (androgen deprivation). |
| D. Chemotherapy Protocol | D1. Chemotherapy regimen | ICIs (Immune Checkpoint Inhibitors), cytotoxic chemotherapy, endocrine therapy. |
|  | D2. Treatment line | Neoadjuvant/adjuvant (ICIs). |
|  | D3. Treatment intent | Immunotherapy (checkpoint inhibitors). |
|  | D4. Number of cycles | Not specified. |
|  | D5. Combination therapies | ICI monotherapy, ICI + anti-CTLA4. |
|  | D6. Dose modifications | Not specified. |
| E. Microbiome Analysis | E1. Sample type | Feces, saliva (PDAC), tumor tissues (PDAC). |
|  | E2. Timing of collection | Not specified (review of multiple studies). |
|  | E3. Sequencing technique | Shotgun metagenomics. 16S rRNA sequencing. qPCR (quantitative polymerase chain reaction). Fluorescence *in situ* hybridization (FISH) (for PDAC). |
|  | E4. Sequencing platform | Not specified (review of multiple studies). |
|  | E5. Sequencing depth | Not specified. |
|  | E6. Bioinformatic pipeline | MetaPhlAn 4 (for taxonomic profiles), pibble models (for mega-analysis), LEfSe (Linear Discriminant Analysis Effect Size), Random Forest. |
|  | E7. Reference database | Not specified (MetaPhlAn uses its own database). |
|  | E8. Quality control | Filtering of artifactual contaminants (breast cancer). |
| F. Predictive Model | F1. Model type | GOMS (Gut OncoMicrobiome Signatures) as predictive biomarkers. Microbiome-based classification models. Transkingdom network. |
|  | F2. Algorithm(s) used | Random Forest, LEfSe, Cox regression analyses. |
|  | F3. Predictive variables | Microbial taxa (SGBs (species-level genome bins), genera, species), relative abundance, diversity (alpha, beta), microbial metabolites (SCFAs, bile acids, tryptophan, L-Arginine, TMAO). Clinical factors (age, sex, study cohort). |
|  | F4. Feature selection method | Not specified (review of multiple studies). |
|  | F5. Model validation | Leave-one-dataset-out cross-validation (all-minus-one). |
|  | F6. Performance metrics | Area Under the Curve (AUC), Hazard Ratio (HR), OR (Odds Ratio). |
|  | F7. Training set size | Not applicable (review of various studies). ICI Meta-analysis: N = 761 patients (for the Random Forest model with sex and age). |
|  | F8. Test set size | Not applicable. |
|  | F9. Model interpretability | LEfSe cladograms, abundance bar plots. |
|  | F10. Model availability | Not applicable. |
| G. Outcomes Assessed | G1. Primary study outcome | Objective Response (ORR), Overall Survival (OS), Progression-Free Survival (PFS). |
|  | G2. Response criteria | RECIST 1.1 (Response Evaluation Criteria in Solid Tumors). |
|  | G3. Evaluation time | Not specified. |
|  | G4. Secondary outcomes | Immune-Related Adverse Events (irAEs), Cachexia, Neurotoxicity, T cell migration, Cytokine release. |
|  | G5. Associated clinical/laboratory biomarkers | PD-L1 (programmed cell death ligand 1), TMB (tumor mutational burden), MMR (DNA mismatch repair), Neutrophil-to-Lymphocyte Ratio (NLR), cytokines (IL-8), sCD14, sST2 (soluble IL-1 receptor-like 1), LPS binding protein (LBP), uric acid, tryptophan levels, kynurenine, serum L-Arginine, TMAO (trimethylamine N-oxide). |
|  | G6. Associated lifestyle/medication factors | PPIs use, antibiotics, anti-inflammatory agents, osmotic laxatives, biguanides, diet, smoking, age, sex. |
|  | G7. Associated host genetic factors | HER2, p53, PI3K–PTEN, KRAS (oncogenes). |
| H. Key Results and Findings | H1. Key microbial taxa/species | Pan-cancer (non-responders): *Enterocloster*, *Hungatella* and *Clostridium* spp., *Pseudoflavonifractor*, *Eisenbergiella*, Veillonellaceae, Eggerthellaceae, Enterobacteriaceae, Erysipelotrichaceae, *Streptococcus* and *Actinomyces*, *Veillonella parvula*, *Eggerthella lenta*, *E. asparagiformis*. Pan-cancer (responders): Lachnospiraceae, Oscillospiraceae (including *Faecalibacterium* spp.), *Bifidobacterium* spp., *Roseburia* spp., *Coprococcus* spp., *Blautia* spp., *Eubacterium* spp., *Dorea* spp., *Akkermansia muciniphila SGB9226*. Breast Cancer: Bacteroidales, Tannerellaceae, Rikenellaceae, Prevotellaceae, Odoribacteraceae, *Bacteroides uniformis*, *Parabacteroides merdae*, *Bacteroides thetaiotaomicron*, *E. bolteae*, *E. clostridioformis*. PDAC: *Streptococcus* and *Veillonella* spp., *Romboutsia timonensis*, *Methanobrevibacter smithii*. CRC: *Solobacterium moorei*, *Fusobacterium nucleatum*, *Parvimonas micra*, *Peptostreptococcus stomatis*, *Peptostreptococcus anaerobius*, *Gemella morbillorum*. NSCLC: *A. muciniphila SGB9226*. Melanoma: *Bifidobacterium pseudocatenulatum*, *Roseburia* spp., *A. muciniphila*. |
|  | H2. Direction of association | Pan-cancer: Enrichment of pro-inflammatory/immunosuppressive taxa in non-responders/cancer patients; enrichment of SCFA-producing/immunostimulatory taxa in responders/healthy individuals. NSCLC: *A. muciniphila* (<4.8% relative abundance) associated with inflamed tumor microenvironment and better outcomes (ORR and OS); (>4.8%) associated with resistance. CRC: Enrichment of *F. nucleatum* and oral species (e.g., *P. stomatis*). Toxicity: Streptococcaceae associated with irAEs. |
|  | H3. Model predictive performance | NSCLC (*A. muciniphila*): Prediction of clinical benefit independent of other prognostic factors. PDAC: Classification with AUC up to 0.84 (27 species). ICI ML (761 patients): Moderate and inconsistent predictive power (AUC 0.71). |
|  | H4. Microbial diversity findings | Cancer patients and comedications generally exhibit lower microbial diversity. *Akkermansia* spp. with >4.8% abundance (due to antibiotic resistance) is associated with dysbiosis. |
|  | H5. Microbial functional/metabolic findings | Cachexia: Fecal SCFA levels reduced; xenobiotic metabolites derived from microorganisms (e.g., phenylalanine and tyrosine fermentation products) increased. Healthy aging: Increased indoles (bacterial tryptophan degradation products). PDAC: Increased isoprenoid biosynthesis (KRAS activity) and ADP-L-glycero-D-manno-heptose (LPS). Metabolites: SCFAs, bile acids, tryptophan (tryptophan, kynurenine, I3A, indole propionic acid), L-Arginine, inosine, urolithins, hypoxanthine, histidine, iron (TMAO). |
|  | H6. Preclinical/*in vitro* findings | *Lactobacillus reuteri* translocates to melanoma, releasing I3A that activates AhR receptors in CD8+ T cells. Microbial inosine potentiates immunotherapy by binding to the A2A receptor in CD8+ T cells. *Clostridium* spp. transforms primary bile acids into secondary ones, dampening NKT response. |
|  | H7. Highlight of Findings: Unique Contribution | This review and meta-analysis article integrates the vast and complex field of GOMS (Gut OncoMicrobiome Signatures), establishing them as next-generation biomarkers for cancer immunotherapy. The "major discovery" is the identification of consistent patterns of GOMS associated with response or resistance to immune checkpoint inhibitors (ICIs) across various cancer types, even with variability among cohorts. The article highlights the microbiome's role in modulating immunity and drug metabolism, and the need for personalized microbiome-based strategies to overcome resistance and manage toxicities, providing a roadmap for future research and clinical translation. |
|  | H8. Validation status (internal/external) | Meta-analysis and mega-analysis of shotgun metagenomic datasets from 30 cancer cohorts (23 studies) and 17 control cohorts (14 studies). |
| I. Discussion/Article Implications | I1. Authors' interpretation | Oncogenesis is associated with gut dysbiosis. The microbiome influences immunosurveillance and ICI response. Comorbidities and medications affect the microbiome. There is a "stress ileopathy state" associated with cancer that leads to dysbiosis. GOMS are shared among diseases and are promising biomarkers. |
|  | I2. Proposed biological mechanisms | Dysbiosis: Alteration of intestinal barrier integrity, bacterial translocation, altered microbial metabolites (SCFAs, bile acids), interaction with immune receptors (AhR, IL-10R). Metabolism: Biosynthesis of C5 isoprenoids (KRAS oncogenesis), ADP-L-glycero-D-manno-heptose (LPS), tryptophan degradation into indoles, metabolism of L-Arginine, inosine, bile acids, choline. |
|  | I3. Correlations with other biomarkers | PD-L1, TMB, MMR (DNA Mismatch Repair), IL-8, sCD14, sST2 (soluble IL-1 receptor-like 1). |
|  | I4. Impact of interventions (if applicable) | Diet: High-fiber (favorable), Western (unfavorable). Antibiotics/PPIs: Reduce diversity and alter microbiome composition. FMT: Improves ICI response. *Lactobacillus reuteri*: Releases I3A, improves ICIs. |
|  | I5. Consistency/Inconsistency of findings | Consistency of GOMS in breast cancer, PDAC, CRC and NSCLC. Inconsistencies in species identification among studies can be resolved with meta-analyses and mega-analyses. |
|  | I6. Transferability/Generalization | GOMS are identified in diverse cancer types and geographical populations. Applicability to other cancer types or populations needs to be validated. |
|  | I7. Causality vs. Association | Preclinical studies provide evidence of causality (e.g., *A. muciniphila* activates immune responses). |
| J. Clinical Application Potential and Barriers | J1. Potential for implementation in practice | GOMS as a screening tool for early diagnosis and patient stratification for immunotherapy. |
|  | J2. Advantages | Non-invasive and cost-effective approach (fecal samples). Complements existing biomarkers. |
|  | J3. Barriers to implementation | Lack of standardization (collection, analysis). Heterogeneity of the microbiota. Regulatory challenges. Need for more longitudinal and functional studies. |
|  | J4. Cost-effectiveness | Metagenomics analysis is cost-effective compared to other biomarkers. |
|  | J5. Recommendations for future research | Randomized clinical trials, longitudinal studies, robust external validation. Develop rapid PCR-based tests. High-resolution microbiome studies. |
| K. Limitations and Bias of the Original Study | K1. Limitations declared by authors | The robustness of GOMS is limited by study heterogeneity (sample size, methods, outcomes). Most cancer studies do not include matched healthy controls. |
|  | K2. Selection bias | Most cancer studies were retrospective, without matched controls. |
|  | K3. Confounding bias | Factors such as age, sex, comorbidities, medications, and performance status can confound results. |
|  | K4. Generalization | Findings may be influenced by geographical and ethnic differences. |
|  | K5. Reproducibility | Despite meta-analysis, standardization is still needed to ensure reproducibility. |

Table S17. Article 17: Gut microbiome development along the colorectal adenoma–carcinoma sequence

| Category | Extracted Variables | Detailing |
| --- | --- | --- |
| A. Study Identification | A1. Lead Author (Year) | Feng Q (2015) |
|  | A2. Title | Gut microbiome development along the colorectal adenoma–carcinoma sequence |
|  | A3. Journal | Nature Communications. |
|  | A4. Country/Region | Austria (Oberndorf Hospital), China (Shenzhen). |
|  | A5. Funding | Natural Science Foundation of China (30890032, 30725008, 30811130531 and 31161130357), Shenzhen Municipal Government of China (BGI20100001, CXB201108250096A and CXB201108250098A), Danish Strategic Research Council grant (2106-07-0021), Ole RØmer grant from the Danish Natural Science Research Council and Solexa project (272-07-0196), SPAR Austria. Type: Public and Private. |
|  | A6. Conflicts of interest | No financial conflicts of interest declared by the authors. |
| B. Methodological Design | B1. Study type | Cross-sectional Metagenomic-Wide Association Study (MGWAS). |
|  | B2. Follow-up duration | Not applicable (cross-sectional study). |
|  | B3. Study center(s) | Multicenter. |
|  | B4. Recruitment period | Between 2010 and 2012. |
|  | B5. Ethical approval | Local ethics committee (Ethikkommission des Landes Salzburg, approval no. 415-E/1262/2-2010). |
| C. Study Population | C1. Sample size | 156 fecal samples analyzed (55 healthy controls, 42 advanced adenoma, 41 carcinoma). 9 additional samples (6 controls, 3 adenoma) used in test sets. |
|  | C2. Age | 45-86 years (Caucasian). |
|  | C3. Sex | Controls: 24 F / 33 M. Advanced adenoma: 22 F / 22 M. Carcinoma: 18 F / 28 M. |
|  | C4. Cancer type | Advanced colorectal adenoma, colorectal carcinoma. |
|  | C5. Tumor stage | Not specified (advanced adenoma, carcinoma). Carcinoma classified by AJCC (American Joint Committee on Cancer) TNM staging system. |
|  | C6. Performance status | Not applicable. |
|  | C7. Relevant comorbidities | Metabolic syndrome. Type 2 diabetes. No probiotics or antibiotics in the last 3 months. |
|  | C8. Previous treatments | Not specified. |
| D. Chemotherapy Protocol | D1. Chemotherapy regimen | Not applicable (study of microbiome and diet association with CRC). |
|  | D2. Treatment line | Not applicable. |
|  | D3. Treatment intent | Not applicable. |
|  | D4. Number of cycles | Not applicable. |
|  | D5. Combination therapies | Not applicable. |
|  | D6. Dose modifications | Not applicable. |
| E. Microbiome Analysis | E1. Sample type | Feces. |
|  | E2. Timing of collection | Single collection at baseline. |
|  | E3. Sequencing technique | Shotgun paired-end metagenomics. |
|  | E4. Sequencing platform | Illumina (insert size 350 bp, read length 100 bp). |
|  | E5. Sequencing depth | 5 GB per sample on average. |
|  | E6. Bioinformatic pipeline | SOAPdenovo v2.04 (contig assembly), GeneMark v2.7d (gene prediction), BLAT (redundant genes), BLASTN v2.2.24 (taxonomic assignment), PAM (partitioning around medoids), Dirichlet multinomial mixture model (DMM), SPIEC-EASI (co-occurrence networks), Cytoscape 3.0.2. |
|  | E7. Reference database | IMG database (v400) for taxonomic assignment. KEGG (Kyoto Encyclopedia of Genes and Genomes) for functional pathways. Virulence Factors of pathogenic bacteria Databases (VFDB) for virulence factors. |
|  | E8. Quality control | High-quality reads. Assembled contigs. Removal of redundant genes. |
| F. Predictive Model | F1. Model type | Random Forest classifier for carcinoma and adenoma detection. |
|  | F2. Algorithm(s) used | Random Forest (randomForest4.6-7 package in R 3.0.2). |
|  | F3. Predictive variables | Metagenomic Linkage Groups (MLGs) (15 MLGs for carcinoma, 10 MLGs for adenoma). Age and BMI (Body Mass Index) were also tested as predictors. |
|  | F4. Feature selection method | 10-fold cross-validation repeated 5 times (50 tests) to select the optimal number of MLGs. |
|  | F5. Model validation | 10-fold cross-validation repeated 5 times (training). Independent test set. |
|  | F6. Performance metrics | Receiver Operating Characteristic Area Under the Curve (AUC). Classification error rate. |
|  | F7. Training set size | Carcinoma: 55 controls, 41 carcinoma. Adenoma: 55 controls, 42 adenoma. |
|  | F8. Test set size | Carcinoma: 8 controls, 47 advanced adenoma, 5 carcinoma. Adenoma: 8 controls, 5 advanced adenoma, 46 carcinoma. |
|  | F9. Model interpretability | Most important MLGs, co-occurrence networks (Cytoscape). |
|  | F10. Model availability | Not specified. |
| G. Outcomes Assessed | G1. Primary study outcome | Identification of fecal microbial signatures associated with advanced adenoma and colorectal carcinoma. |
|  | G2. Response criteria | Histopathology (tubular adenoma, advanced adenoma, carcinoma). |
|  | G3. Evaluation time | Not applicable (cross-sectional study). |
|  | G4. Secondary outcomes | Influence of risk factors (diet, smoking, obesity) on the microbiome. Microbiome richness and diversity. |
|  | G5. Associated clinical/laboratory biomarkers | Serum ferritin, C-reactive protein (CRP), hemoglobin (Hb), fasting glucose, fasting insulin, HOMA index (Homeostasis Model Assessment), triglycerides (TG), cholesterol (HDL, LDL), Gamma-glutamyl transferase (GGT), Alanine Aminotransferase (ALT), Aspartate Aminotransferase (AST). |
|  | G6. Associated lifestyle/medication factors | Red meat consumption, fruit and vegetable consumption, dietary fiber, smoking, physical activity, waist circumference, waist-to-hip ratio (WHR). |
|  | G7. Associated host genetic factors | Not specified (except cancer-related mutations in other studies). |
| H. Key Results and Findings | H1. Key microbial taxa/species | Enriched in carcinoma: MLGs of *Bacteroides*, *Parabacteroides*, *Alistipes putredinis*, *Bilophila wadsworthia*, *Lachnospiraceae bacterium*, *Escherichia coli*. Oral anaerobes (*Fusobacterium sp. oral taxon 370*, *Parvimonas micra*, *Gemella morbillorum* and *Peptostreptococcus stomatis*). Decreased in carcinoma: *Bifidobacterium animalis*, *Streptococcus thermophilus*, *Ruminococcus*, *Streptococcus*. |
|  | H2. Direction of association | Gene and genus richness of the microbiome increases from controls to adenoma, and from adenoma to carcinoma. Specific MLGs are significantly elevated in carcinoma. |
|  | H3. Model predictive performance | Carcinoma classifier: AUC = 96% (in test set). Adenoma classifier: AUC = 87.38% (in training set), AUC = 59.56% (in test set). |
|  | H4. Microbial diversity findings | Gene and genus richness significantly different between the 3 groups (P = 0.005 and P = 3.2e–7, respectively). Alpha-diversity not significantly different. Higher richness is not a sign of a healthy microbiome. |
|  | H5. Microbial functional/metabolic findings | Carcinoma-associated: SCFA production from amino acids, bile acid metabolism. Control-associated: Butyrate production. KEGG pathways: Phosphotransferase modules, sugar transporters (decreased in carcinoma), amino acid transport (Histidine, Arginine, Lysine increased in carcinoma), mucin degradation (dermatan sulfate, heparan sulfate, keratan sulfate increased in carcinoma). Methanogenesis increased in adenoma/carcinoma. |
|  | H6. Preclinical/*in vitro* findings | Not applicable (human study). |
|  | H7. Highlight of Findings: Unique Contribution | This metagenomic-wide association study (MGWAS) maps the evolution of the gut microbiome along the adenoma-colorectal carcinoma sequence, revealing that increased microbial richness and enrichment of oral species are characteristics of cancer. The "major discovery" is the identification of Metagenomic Linkage Groups (MLGs) that serve as biomarkers with high diagnostic potential for early detection of adenoma and colorectal carcinoma, and the demonstration of how dietary factors (red meat consumption versus vegetables) directly influence cancer-associated microbial composition. This opens the way for non-invasive diagnostic and preventive interventions. |
|  | H8. Validation status (internal/external) | Internal validation (10-fold cross-validation) and in separate test set. |
| I. Discussion/Article Implications | I1. Authors' interpretation | The gut microbiome undergoes profound changes before and during colorectal cancer development. Higher gene and genus richness is a warning sign for microbiome health in CRC. |
|  | I2. Proposed biological mechanisms | Carcinogenic bacteria (*F. nucleatum*) bind to epithelial cell E-cadherin, stimulating Wnt/β-catenin signaling. Increased bacterial metabolites (e.g., secondary bile acids) can be carcinogenic. Red meat consumption selects for putrefactive bacteria that can promote colorectal carcinoma. |
|  | I3. Correlations with other biomarkers | Correlation between CRP (C-reactive protein) and meat consumption with MLGs enriched in carcinoma. Serum ferritin and hemoglobin (Hb) negatively correlated with MLGs enriched in carcinoma. |
|  | I4. Impact of interventions (if applicable) | Does not discuss interventions. |
|  | I5. Consistency/Inconsistency of findings | Consistent with previous 16S rRNA pyrosequencing analyses in adenoma. The role of *Akkermansia* in CRC is still unclear. |
|  | I6. Transferability/Generalization | Findings were replicated in two distinct populations (Austria and China). Applicability to other populations and cancer types needs to be validated. |
|  | I7. Causality vs. Association | The study establishes associations, but the authors point to evidence of causality in other studies. |
| J. Clinical Application Potential and Barriers | J1. Potential for implementation in practice | Development of non-invasive and early diagnostic tools for adenoma and colorectal carcinoma. |
|  | J2. Advantages | Non-invasive early detection, allowing intervention. |
|  | J3. Barriers to implementation | The adenoma classifier still requires additional examinations for confirmation. |
|  | J4. Cost-effectiveness | Not discussed. |
|  | J5. Recommendations for future research | Studies to resolve the role of *Akkermansia* in CRC. Research on how diet, obesity, and smoking collectively act on the microbiome in colorectal carcinoma development. |
| K. Limitations and Bias of the Original Study | K1. Limitations declared by authors | A formal power analysis for sample size calculation was not performed. Functional enrichment analysis revealed more microbial genes than metabolites. |
|  | K2. Selection bias | Not explicitly stated. |
|  | K3. Confounding bias | Not applicable. |
|  | K4. Generalization | The applicability of markers in other cohorts and populations still needs to be validated. |
|  | K5. Reproducibility | Metagenomic sequencing data deposited in the EBI database (accession ERP008729). |

Table S18. Article 18: The influence of the gut microbiome on cancer, immunity, and cancer immunotherapy

| Category | Extracted Variables | Detailing |
| --- | --- | --- |
| A. Study Identification | A1. Lead Author (Year) | Gopalakrishnan V (2018) |
|  | A2. Title | The influence of the gut microbiome on cancer, immunity, and cancer immunotherapy |
|  | A3. Journal | Cancer Cell. |
|  | A4. Country/Region | USA (Houston) |
|  | A5. Funding | J.A.W. supported by Binational Science Foundation, Melanoma Research Alliance, Stand Up To Cancer, MD Anderson Cancer Center Multidisciplinary Research Program Grant, and MD Anderson Cancer Center’s Melanoma Moon Shots Program. J.A.W. is a member of the Parker Institute for Cancer Immunotherapy at MD Anderson Cancer Center. A.R. supported by Kimberley Clarke Foundation Award for Scientific Achievement (Odyssey Fellowship program at MD Anderson Cancer Center). Type: Public and Private. |
|  | A6. Conflicts of interest | Yes. J.A.W. (honoraria, advisory board, patent inventor), V.G. (consultant, patent inventor). |
| B. Methodological Design | B1. Study type | Review |
|  | B2. Follow-up duration | Not applicable (review). |
|  | B3. Study center(s) | Not applicable (review). |
|  | B4. Recruitment period | Not applicable (review). |
|  | B5. Ethical approval | Not applicable (review). |
| C. Study Population | C1. Sample size | Not applicable (review of various studies in humans and animal models). |
|  | C2. Age | Not applicable. |
|  | C3. Sex | Not applicable. |
|  | C4. Cancer type | Melanoma, Non-Small Cell Lung Cancer (NSCLC), Renal Cell Carcinoma (RCC), Urothelial Cancer. |
|  | C5. Tumor stage | Advanced. |
|  | C6. Performance status | Not applicable. |
|  | C7. Relevant comorbidities | Graft-versus-host disease (GVHD). |
|  | C8. Previous treatments | Antibiotics (ABT), Immune Checkpoint Inhibitors (ICIs). |
| D. Chemotherapy Protocol | D1. Chemotherapy regimen | Immunotherapy (checkpoint inhibitors: anti-CTLA-4, anti-PD-1/PD-L1), chemotherapy (platinum agents, cyclophosphamide). |
|  | D2. Treatment line | Not specified (various treatment contexts). |
|  | D3. Treatment intent | Immunotherapy, chemotherapy. |
|  | D4. Number of cycles | Not specified. |
|  | D5. Combination therapies | Not specified (combination immunotherapy, immunotherapy with chemotherapy). |
|  | D6. Dose modifications | Not specified. |
| E. Microbiome Analysis | E1. Sample type | Feces (humans, murine models), Mesenteric lymph nodes (murine models), Tumor tissue (murine models). |
|  | E2. Timing of collection | Before, during, and after treatment (context of antibiotic use). |
|  | E3. Sequencing technique | Metagenomic sequencing (human). |
|  | E4. Sequencing platform | Not specified (review of multiple studies). |
|  | E5. Sequencing depth | Not specified. |
|  | E6. Bioinformatic pipeline | Not specified. |
|  | E7. Reference database | Not specified. |
|  | E8. Quality control | Not specified. |
| F. Predictive Model | F1. Model type | Not applicable (review discusses biomarkers, not a specific predictive model developed in this article). |
|  | F2. Algorithm(s) used | Not applicable. |
|  | F3. Predictive variables | Microbial taxa (e.g., *Faecalibacterium*, *Ruminococcus*, *Blautia*, *Enterococcus*, *Streptococcus*, *Bifidobacterium*, *Bacteroides fragilis*, *Bacteroides thetaiotaomicron*, *Burkholderia cepacia*, *Akkermansia muciniphila*, *Alistipes*, *Collinsella aerofaciens*, *Porphyromonas pasteri*, *Actinomyces viscosus*, *Garnderella vaginalis*). |
|  | F4. Feature selection method | Not applicable (review of evidence). |
|  | F5. Model validation | Fecal Microbiota Transplant (FMT) in murine models (germ-free, with ABT) for causal validation. |
|  | F6. Performance metrics | Not applicable. |
|  | F7. Training set size | Not applicable. |
|  | F8. Test set size | Not applicable. |
|  | F9. Model interpretability | Phylogenetic tree (Figure 2 in the article). |
|  | F10. Model availability | Not applicable. |
| G. Outcomes Assessed | G1. Primary study outcome | Not applicable (review). |
|  | G2. Response criteria | Not specified (review of multiple studies). |
|  | G3. Evaluation time | Not specified. |
|  | G4. Secondary outcomes | Survival (PFS and OS), Toxicity (adverse events, GVHD), Immunity (CD4+, CD8+, Th17, Tregs, DCs - dendritic cells). |
|  | G5. Associated clinical/laboratory biomarkers | L-tryptophan, 3-indoxyl sulfate, IgA (immunoglobulin A), HLA (human leukocyte antigen). |
|  | G6. Associated lifestyle/medication factors | Antibiotics, diet, probiotics, FMT. |
|  | G7. Associated host genetic factors | Not specified. |
|  | G8. Intervention results (if applicable) | ABT: Impact on microbiome composition and outcomes. Probiotics (*Lactobacillus rhamnosus GG*): GVHD reduction. FMT: ICI response improvement. |
| H. Key Results and Findings | H1. Key microbial taxa/species | Associated with ICI Response: Clostridiales, Ruminococcaceae, *Faecalibacterium*, *Bifidobacterium longum*, *Collinsella aerofaciens*, *Enterococcus faecium*, *Akkermansia muciniphila*, *Alistipes*, *Porphyromonas pasteri*. Associated with ICI Non-Response: Bacteroidales, *Actinomyces viscosus*, *Garnderella vaginalis*. Associated with Toxicity: *Faecalibacterium prausnitzii*, other Firmicutes (higher colitis risk), Bacteroidetes (lower colitis incidence). In HSCT: Reduction of *Faecalibacterium* and *Ruminococcus*, increase of *Enterococcus*, *Streptococcus*, Proteobacteria (dysbiosis). *Blautia* (Clostridia) associated with reduced GVHD lethality. |
|  | H2. Direction of association | Microbiome with high diversity and favorable taxa (e.g., *Faecalibacterium*, Ruminococcaceae) associated with better response and survival. Unfavorable taxa (e.g., Bacteroidales) associated with lack of response. ABT harms response. *Bacteroides fragilis* and *Burkholderia cepacia* enhance anti-CTLA-4 action. *Bifidobacterium* restores anti-PDL1 efficacy. |
|  | H3. Model predictive performance | Not applicable (review). |
|  | H4. Microbial diversity findings | Patients responding to anti-PD-1 had significantly higher microbial diversity in the gut (Gopalakrishnan et al., 2018). Post-HSCT dysbiosis: loss of diversity, reduction of obligate anaerobes and increase of *Enterococcus*, *Streptococcus*, Proteobacteria. |
|  | H5. Microbial functional/metabolic findings | 3-indoxyl sulfate (L-tryptophan metabolite by microbiota) as a marker of dysbiosis. SCFAs (e.g., butyrate) enhance immunity via IgA, histone deacetylase inhibition. |
|  | H6. Preclinical/*in vitro* findings | Murine models (germ-free, with ABT) are widely used to study interactions. Oral gavage of *Bacteroides fragilis* and *Burkholderia cepacia* enhanced anti-CTLA-4 action. Oral *Bifidobacterium* supplementation restored anti-PDL1 efficacy. |
|  | H7. Highlight of Findings: Unique Contribution | This review consolidates evidence that the gut microbiome is a dominant mediator of response and toxicity to oncological immunotherapy, highlighting that interactions extend beyond ICI therapies, also covering chemotherapies and HSCT. The "major discovery" is the recognition that the microbiome not only influences systemic immunity but is also manipulable to enhance therapeutic responses. The article provides a clear framework on how microbiota modulation, through diet, probiotics, or FMT, emerges as a promising adjuvant strategy in the era of immunotherapy, pointing to the need for standardized approaches and rigorous clinical trials. |
|  | H8. Validation status (internal/external) | Not applicable (review). |
| I. Discussion/Article Implications | I1. Authors' interpretation | The gut microbiome modulates host immunity and, consequently, the response and toxicity to various forms of cancer therapy. It is an extrinsic tumor factor that significantly impacts outcomes. |
|  | I2. Proposed biological mechanisms | Local immunity: PAMPs (pathogen-associated molecular patterns) interact with PRRs (pattern recognition receptors) in intestinal epithelial cells. SCFAs enhance immunity via IgA, histone deacetylase inhibition. Adaptive immunity: DCs (dendritic cells) activate CD4+ (Tregs, Th17) and CD8+ T cells in mesenteric lymph nodes. Systemic immunity: TLR signaling generates cytokines and interferons. |
|  | I3. Correlations with other biomarkers | 3-indoxyl sulfate (biomarker of dysbiosis), IgA. |
|  | I4. Impact of interventions (if applicable) | Diet: Dietary changes can modulate microbiome composition and immune response. Probiotics: Can increase beneficial bacteria abundance and modulate immunity. FMT: Direct means of manipulating microbiota, with potential to overcome immunotherapy resistance and mitigate toxicity. |
|  | I5. Consistency/Inconsistency of findings | Inconsistencies in identifying specific taxa may be due to technical and geographical differences among studies. |
|  | I6. Transferability/Generalization | Findings were replicated in various murine models and human cohorts, demonstrating the generalizability of microbiome impact. |
|  | I7. Causality vs. Association | Murine models and FMT in patients provide evidence of causality. |
| J. Clinical Application Potential and Barriers | J1. Potential for implementation in practice | Modulation of the gut microbiome as an adjuvant for anticancer therapies. |
|  | J2. Advantages | Improved responses and reduced toxicity of therapies. |
|  | J3. Barriers to implementation | It is still unclear which microbiome composition is optimal. Variability in probiotic formulations. Need for rigorous clinical trials and standardization. |
|  | J4. Cost-effectiveness | Not discussed. |
|  | J5. Recommendations for future research | Studies to dissect molecular interactions and mechanisms. Evaluation of factors impacting the microbiome (diet, medications, mental health). Development of ideal bacterial consortia. Optimization of preparatory regimens before microbiome modulation. |
| K. Limitations and Bias of the Original Study | K1. Limitations declared by authors | Review, does not develop original study with its own limitations. |
|  | K2. Selection bias | Not applicable (review). |
|  | K3. Confounding bias | Not applicable (review). |
|  | K4. Generalization | Not applicable (review). |
|  | K5. Reproducibility | Not applicable (review). |

Table S19. Article 19: Impact of the gut microbiome on response and toxicity to chemotherapy in advanced esophageal cancer

| Category | Extracted Variables | Detailing |
| --- | --- | --- |
| A. Study Identification | A1. Lead Author (Year) | Li N (2024) |
|  | A2. Title | Impact of the gut microbiome on response and toxicity to chemotherapy in advanced esophageal cancer |
|  | A3. Journal | Heliyon. |
|  | A4. Country/Region | China |
|  | A5. Funding | National High Level Hospital Clinical Research Funding (2022-PUMCH-A-212). Type: Public. |
|  | A6. Conflicts of interest | No financial or personal conflicts of interest declared by the authors. |
| B. Methodological Design | B1. Study type | Prospective cohort study. |
|  | B2. Follow-up duration | Not specified. |
|  | B3. Study center(s) | Unicenter (Peking Union Medical College Hospital (PUMCH)). |
|  | B4. Recruitment period | Between 2018 and 2020. |
|  | B5. Ethical approval | PUMCH Ethics Committee (JS-2745). |
| C. Study Population | C1. Sample size | 31 patients with Esophageal Squamous Cell Carcinoma (ESCC). 45 fecal samples (31 baseline, 14 post-treatment). |
|  | C2. Age | 49–72 years. |
|  | C3. Sex | Male: 27/31, Female: 4/31. |
|  | C4. Cancer type | Advanced Esophageal Squamous Cell Carcinoma (ESCC). |
|  | C5. Tumor stage | Advanced (locally advanced or metastatic) according to American Joint Committee on Cancer (AJCC) 7.0. |
|  | C6. Performance status | Not specified. |
|  | C7. Relevant comorbidities | Excluded patients with intestinal obstruction, infection, inflammatory bowel disease, chronic disease, antibiotic/probiotic use in the last month. |
|  | C8. Previous treatments | Not specified. |
| D. Chemotherapy Protocol | D1. Chemotherapy regimen | TP (paclitaxel 175 mg/m2 on day 1; cisplatin 25 mg/m2 on days 1–3). |
|  | D2. Treatment line | Not specified (patients with advanced or metastatic disease). |
|  | D3. Treatment intent | Systemic chemotherapy. |
|  | D4. Number of cycles | Every three weeks. |
|  | D5. Combination therapies | Not applicable (TP regimen is combination chemotherapy). |
|  | D6. Dose modifications | Grade 3–4 Adverse Events (AEs) were observed, leading to dose modifications (not specified). |
| E. Microbiome Analysis | E1. Sample type | Feces. |
|  | E2. Timing of collection | Baseline (within 3 days before chemotherapy) and after treatment (6 weeks after chemotherapy initiation). |
|  | E3. Sequencing technique | 16S rRNA sequencing (V4 region). |
|  | E4. Sequencing platform | Illumina HiSeq2500. |
|  | E5. Sequencing depth | Total of 7,224,168 tags from 45 samples. |
|  | E6. Bioinformatic pipeline | FLASH software (v1.2.11), USEARCH software (v7.0.1090). |
|  | E7. Reference database | GreenGene Database (V201305). |
|  | E8. Quality control | Removal of low-quality reads. Tags clustered into Operational Taxonomic Units (OTUs) with 97% similarity. |
| F. Predictive Model | F1. Model type | Microbial signature for chemotherapy efficacy and toxicity prediction. Multiparametric model for toxicity. |
|  | F2. Algorithm(s) used | Wilcoxon rank-sum test. ROC (Receiver Operating Characteristic) curve analysis. |
|  | F3. Predictive variables | Microbial taxa (species *Bacteroides plebeius*, *Bacteroides ovatus*, *Bacteroides uniformis*). |
|  | F4. Feature selection method | Wilcoxon rank-sum test for abundance differences. |
|  | F5. Model validation | Not specified. |
|  | F6. Performance metrics | Area Under the Curve (AUC), Sensitivity, Specificity, P-value. |
|  | F7. Training set size | Not applicable. |
|  | F8. Test set size | Not applicable. |
|  | F9. Model interpretability | Not specified (model is based on specific taxa). |
|  | F10. Model availability | Not specified. |
| G. Outcomes Assessed | G1. Primary study outcome | Chemotherapy efficacy (partial response (PR), stable disease (SD), progressive disease (PD)). Toxicity. |
|  | G2. Response criteria | RECIST 1.1 (Response Evaluation Criteria in Solid Tumors) for efficacy. NCI (National Cancer Institute) CTCAE (Common Terminology Criteria for Adverse Events) for toxicity. |
|  | G3. Evaluation time | 6 weeks after chemotherapy initiation for efficacy. |
|  | G4. Secondary outcomes | Progression-Free Survival (PFS), Overall Survival (OS), microbial diversity. |
|  | G5. Associated clinical/laboratory biomarkers | Not specified. |
|  | G6. Associated lifestyle/medication factors | Not specified (patients maintained oral diet). |
|  | G7. Associated host genetic factors | Not specified. |
| H. Key Results and Findings | H1. Key microbial taxa/species | *Bacteroides plebeius*, *Bacteroides ovatus*, *Bacteroides uniformis*. *Akkermansia muciniphila* (*A. muciniphila*), *Pyramidobacter piscolens*. *Clostridium colinum*, *Ruminococcus calidus* (*R. calidus*). |
|  | H2. Direction of association | Efficacy: *Bacteroides plebeius* and *Bacteroides ovatus* associated with better efficacy. *B. plebeius* significantly higher in partial responders (PR) (P = 0.043) and overall responders (R) (P = 0.045). Toxicity: *Bacteroides plebeius* and *Bacteroides uniformis* associated with Grade 3-4 toxicity. Stage: *Bacteroides acidifaciens* significantly higher in patients with distant metastases (P = 0.009). *Clostridium colinum* (P = 0.024) and *R. calidus* (P = 0.008) decreased in patients with metastases. Post-chemotherapy: *A. muciniphila* significantly increased (P = 0.013), *P. piscolens* decreased (P = 0.043). |
|  | H3. Model predictive performance | *B. ovatus* (PR vs. non-PR): Sensitivity = 83.3%, Specificity = 69.6%, AUC = 0.790 (95% CI: 0.602–0.977), P = 0.031. *B. plebeius* (R vs. non-R): AUC = 0.865 (95% CI: 0.723–0.999), P = 0.041 (Sensitivity = 73.1%, Specificity = 100%). *B. plebeius* (Grade 3-4 Toxicity): Sensitivity = 85.7%, Specificity = 63.6%, AUC = 0.750 (95% CI: 0.529–0.971), P = 0.049. *B. uniformis* (Grade 3-4 Toxicity): Sensitivity = 85.7%, Specificity = 72.7%, AUC = 0.779 (95% CI: 0.609–0.949), P = 0.028. Multiparametric model (*B. plebeius* + *B. uniformis*) for Grade 3-4 toxicity: AUC = 0.825 (95% CI: 0.664–0.986), P = 0.011 (Sensitivity = 85.7%, Specificity = 77.3%). |
|  | H4. Microbial diversity findings | No significant difference in alpha diversity (Shannon index) between patients with locally advanced and metastatic disease. No significant difference in alpha diversity or microbial composition between baseline and post-TP chemotherapy. |
|  | H5. Microbial functional/metabolic findings | Not directly assessed. |
|  | H6. Preclinical/*in vitro* findings | Not applicable. |
|  | H7. Highlight of Findings: Unique Contribution | This prospective study identifies specific microbial signatures in the baseline gut microbiome that predict the efficacy and toxicity of TP chemotherapy (paclitaxel and cisplatin) in advanced ESCC patients. The "major discovery" is the identification of *Bacteroides plebeius* and *Bacteroides ovatus* as predictors of good response, and of *Bacteroides plebeius* and *Bacteroides uniformis* as predictors of severe toxicity. The development of a multiparametric predictive model for toxicity demonstrates the microbiome's potential as a crucial non-invasive biomarker for patient stratification and personalized treatment management in ESCC. |
|  | H8. Validation status (internal/external) | Internal validation (ROC curve analysis). |
| I. Discussion/Article Implications | I1. Authors' interpretation | The abundance of specific bacteria in the baseline gut microbiome is associated with TP chemotherapy efficacy and toxicity in ESCC. These findings can establish a microbial signature to predict clinical outcomes. |
|  | I2. Proposed biological mechanisms | *Bacteroides* spp. have been associated with cancer progression. *Clostridia* spp. play a role in activating intracellular signaling pathways. Species of the Ruminococcaceae family have been identified as positive prognostic factors in immunotherapy (involvement in the production and metabolism of secondary bile acids). |
|  | I3. Correlations with other biomarkers | Not explored. |
|  | I4. Impact of interventions (if applicable) | Not discussed. |
|  | I5. Consistency/Inconsistency of findings | Finding of *Bacteroides acidifaciens* increased in distant metastases and *Clostridium colinum* and *Ruminococcus calidus* decreased is consistent with the role of *Bacteroides* in progression and the protective effect of Clostridia and Ruminococcaceae. Failure to detect changes in alpha diversity after chemotherapy is inconsistent with other studies. |
|  | I6. Transferability/Generalization | Findings are specific to ESCC and the TP regimen. |
|  | I7. Causality vs. Association | The study identifies associations. Causality and exact mechanisms need to be verified by further functional research. |
| J. Clinical Application Potential and Barriers | J1. Potential for implementation in practice | Biomarkers to predict chemotherapy efficacy and toxicity in ESCC. |
|  | J2. Advantages | Potential for stratification and personalized treatment management. |
|  | J3. Barriers to implementation | Need for more validation in large cohorts and mechanistic studies. Inconsistency of findings among studies on the esophageal microbiome. |
|  | J4. Cost-effectiveness | Not discussed. |
|  | J5. Recommendations for future research | Studies to identify mechanisms underlying the microbiome's impact on chemotherapy. |
| K. Limitations and Bias of the Original Study | K1. Limitations declared by authors | Small sample size. Heterogeneity and confounding factors (diet, nutritional status, age, physical activity). Lack of animal models to identify mechanisms. |
|  | K2. Selection bias | Not explicitly stated. |
|  | K3. Confounding bias | Control for diet, nutritional status, age, and physical activity not specified. |
|  | K4. Generalization | Findings are specific to ESCC and the TP regimen. |
|  | K5. Reproducibility | Data available online (https://doi.org/10.1016/j.heliyon.2024.e32770). |

Table S20. Article 20: Regorafenib plus toripalimab in patients with metastatic colorectal cancer: a phase Ib/II clinical trial and gut microbiome analysis

| Category | Extracted Variables | Detailing |
| --- | --- | --- |
| A. Study Identification | A1. Lead Author (Year) | Wang F (2021) |
|  | A2. Title | Regorafenib plus toripalimab in patients with metastatic colorectal cancer: a phase Ib/II clinical trial and gut microbiome analysis |
|  | A3. Journal | Cell Reports Medicine. |
|  | A4. Country/Region | China |
|  | A5. Funding | Shanghai Junshi Biosciences (sponsor). National Natural Science Foundation of China (81930065, 81872011, 81903163, 81802438, 31801037), Science and Technology Program of Guangdong (2019B020227002), Science and Technology Program of Guangzhou (201904020046, 201803040019, 201704020228), China Postdoctoral Science Foundation (2019M663306), Sun Yat-sen University Clinical Research 5010 Program (2018014). Type: Industrial and Public. |
|  | A6. Conflicts of interest | No financial or personal conflicts of interest declared by the authors. |
| B. Methodological Design | B1. Study type | Phase Ib/II clinical trial with gut microbiome analysis. |
|  | B2. Follow-up duration | Not specified (data cutoff of July 12, 2020). |
|  | B3. Study center(s) | Unicenter (Sun Yat-sen University Cancer Center). |
|  | B4. Recruitment period | March 2019 to January 2020. |
|  | B5. Ethical approval | Approved by the ethics committee of Sun Yat-sen University Cancer Center (ID: B2019003-05). |
| C. Study Population | C1. Sample size | 42 patients with Metastatic Colorectal Cancer (mCRC) recruited (12 in Phase Ib, 30 in Phase II). 32 patients for microbiome analysis. |
|  | C2. Age | Median 53 years (range 37–69) for 80 mg regorafenib. Median 44 years (range 37–55) for 120 mg regorafenib. |
|  | C3. Sex | Male: 51.3% (80 mg regorafenib), 66.7% (120 mg regorafenib). Female: 48.7% (80 mg), 33.3% (120 mg). |
|  | C4. Cancer type | Metastatic colorectal cancer (mCRC). |
|  | C5. Tumor stage | Metastatic. |
|  | C6. Performance status | ECOG (Eastern Cooperative Oncology Group) PS 0 (7.7%), PS 1 (92.3%). |
|  | C7. Relevant comorbidities | Not specified. |
|  | C8. Previous treatments | Refractory or intolerant to fluorouracil, oxaliplatin, and irinotecan. Median of 2.40 previous treatment lines. |
| D. Chemotherapy Protocol | D1. Chemotherapy regimen | Regorafenib (80 mg, 120 mg or 160 mg, p.o., qd (D1-D21), q4w) and Toripalimab (3 mg/kg, i.v., D1 and D15, q4w). |
|  | D2. Treatment line | Salvage therapy (after 2 or more previous lines). |
|  | D3. Treatment intent | Systemic therapy. |
|  | D4. Number of cycles | Not specified. |
|  | D5. Combination therapies | Regorafenib (kinase inhibitor) + Toripalimab (anti-PD-1). |
|  | D6. Dose modifications | Three DLTs (dose-limiting toxicities) occurred in 3 (100%) patients in the 120 mg regorafenib cohort. One DLT occurred in 9 patients in the 80 mg regorafenib cohort. |
| E. Microbiome Analysis | E1. Sample type | Feces. |
|  | E2. Timing of collection | Baseline (pre-treatment). |
|  | E3. Sequencing technique | 16S rRNA sequencing (V3-V4 regions). |
|  | E4. Sequencing platform | Illumina HiSeq. |
|  | E5. Sequencing depth | Not specified. |
|  | E6. Bioinformatic pipeline | USEARCH software (version 11), R package (v3.6.1), survminer, survival, ggplot2, forestmodel. |
|  | E7. Reference database | Silva rRNA gene database (release 132). |
|  | E8. Quality control | Raw reads analyzed with USEARCH for quality control, OTU (Operational Taxonomic Units) clustering, and taxonomic annotation. |
| F. Predictive Model | F1. Model type | Risk model for PFS. |
|  | F2. Algorithm(s) used | Cox Proportional Hazards Regression (CoxPH) (multivariate and univariate). |
|  | F3. Predictive variables | Microbial taxa (genus *Fusobacterium*, genus *Alistipes*), clinical factors (Body Mass Index (BMI)). |
|  | F4. Feature selection method | Optimal cutoff value (surv_cutpoint()) for *Fusobacterium* abundance. Variable selection with P ≤ 0.2 in univariate analysis and "step" function in R. |
|  | F5. Model validation | Not specified. |
|  | F6. Performance metrics | Hazard Ratio (HR), P-value, AUC (Area Under the Curve) in time-dependent ROC (Receiver Operating Characteristic) curves. |
|  | F7. Training set size | Not specified (32 patients for microbiome analysis). |
|  | F8. Test set size | Not specified. |
|  | F9. Model interpretability | Forest plot for multivariate CoxPH analysis. |
|  | F10. Model availability | Not specified. |
| G. Outcomes Assessed | G1. Primary study outcome | Tolerability and safety (MTD (Maximum Tolerated Dose), DLT). ORR (Objective Response Rate) in Phase II. |
|  | G2. Response criteria | RECIST 1.1 (Response Evaluation Criteria in Solid Tumors). CTCAE V5.0 (Common Terminology Criteria for Adverse Events). |
|  | G3. Evaluation time | Every 8 weeks by computed tomography (CT). |
|  | G4. Secondary outcomes | PFS (Progression-Free Survival), OS (Overall Survival), DoR (Duration of Response), DCR (Disease Control Rate), Treatment-Related Adverse Events (TRAEs), Immune-Related Adverse Events (irAEs). |
|  | G5. Associated clinical/laboratory biomarkers | Mismatch Repair (MMR)/Microsatellite Instability (MSI) status. RAS/BRAF mutations. |
|  | G6. Associated lifestyle/medication factors | Not specified. |
|  | G7. Associated host genetic factors | RAS mutation, BRAF. |
| H. Key Results and Findings | H1. Key microbial taxa/species | *Fusobacterium* (genus), *Alistipes* (genus). Phyla: Fusobacteriota, Proteobacteria, Firmicutes, Bacteroidetes. |
|  | H2. Direction of association | *Fusobacterium*: Relative abundance and detection rate significantly increased in non-responders (NRs) vs. responders (Rs). Patients with high *Fusobacterium* abundance had shorter PFS. *Alistipes*: Elevated *Alistipes* level associated with worse PFS. |
|  | H3. Model predictive performance | Risk model for PFS (BMI, *Fusobacterium*, *Alistipes*): Time-dependent AUCs of 0.77 (3 months) and 0.80 (6 months). HR = 2.68 (95% CI: 1.11–6.48; P = 0.03) for *Fusobacterium*. HR = 2.56 (95% CI: 1.1–5.94; P = 0.03) for *Alistipes*. HR = 0.39 (95% CI: 0.17–0.88; P = 0.02) for BMI. PFS for risk groups: Median PFS of 1.97 months (high-risk group) vs. 4.2 months (low-risk group) (P = 0.005). |
|  | H4. Microbial diversity findings | Alpha-diversity (Shannon index) significantly reduced in responders (P < 0.001). Beta-diversity had no significant difference between R and NR. |
|  | H5. Microbial functional/metabolic findings | Not directly assessed. |
|  | H6. Preclinical/*in vitro* findings | Not applicable. |
|  | H7. Highlight of Findings: Unique Contribution | This Phase Ib/II clinical trial demonstrates the feasibility and preliminary efficacy of the regorafenib and toripalimab (anti-PD-1) combination for refractory mCRC, and, crucially, identifies *Fusobacterium* as a negative predictive biomarker of response and survival. The "major discovery" is evidence that high baseline *Fusobacterium* abundance is associated with shorter PFS, and that a multifactorial risk model (BMI, *Fusobacterium*, *Alistipes*) can predict PFS. This suggests a microbiome-based patient stratification approach to optimize treatment selection. |
|  | H8. Validation status (internal/external) | Not specified. |
| I. Discussion/Article Implications | I1. Authors' interpretation | The regorafenib + toripalimab combination is safe and shows preliminary efficacy in refractory mCRC. The gut microbiome, especially *Fusobacterium*, is a predictive biomarker of response and survival. |
|  | I2. Proposed biological mechanisms | *Fusobacterium* spp. are considered opportunistic pathogens and can promote colorectal cancer progression. |
|  | I3. Correlations with other biomarkers | BMI was a protective factor with HR of 0.39 (95% CI: 0.17–0.88; P = 0.02). |
|  | I4. Impact of interventions (if applicable) | Does not discuss interventions. |
|  | I5. Consistency/Inconsistency of findings | The combination's safety profile is comparable to regorafenib monotherapy. ORR was lower than statistically assumed. |
|  | I6. Transferability/Generalization | Not explicitly discussed. |
|  | I7. Causality vs. Association | The study identifies associations. Causality and exact mechanisms need to be verified by further functional research. |
| J. Clinical Application Potential and Barriers | J1. Potential for implementation in practice | *Fusobacterium* can be a biomarker for patient stratification. Patients with exclusive pulmonary metastasis may benefit more. |
|  | J2. Advantages | Potential to stratify patients and optimize treatment. |
|  | J3. Barriers to implementation | Small sample size for microbiome analysis. Lack of analysis of dynamic microbiome changes. |
|  | J4. Cost-effectiveness | Not discussed. |
|  | J5. Recommendations for future research | Additional investigations in larger cohorts for the combination. |
| K. Limitations and Bias of the Original Study | K1. Limitations declared by authors | Small sample size and patient selection (good ECOG PS). Lack of dynamic microbiome change analysis. |
|  | K2. Selection bias | Not explicitly stated. |
|  | K3. Confounding bias | Not explicitly stated. |
|  | K4. Generalization | The population is Chinese, which may limit generalization. |
|  | K5. Reproducibility | Raw 16S rRNA sequencing data deposited in the NCBI database (PRJNA698295). |
